# Interrogating data-independent acquisition LC–MS/MS for affinity proteomics

**DOI:** 10.1007/s42485-024-00166-4

**Published:** 2024-09-17

**Authors:** David L. Tabb, Mohammed Hanzala Kaniyar, Omar G. Rosas Bringas, Heaji Shin, Luciano Di Stefano, Martin S. Taylor, Shaoshuai Xie, Omer H. Yilmaz, John LaCava

**Affiliations:** 1 European Research Institute for the Biology of Ageing, University Medical Center Groningen, Groningen, The Netherlands; 2 Department of Biology, David H. Koch Institute for Integrative Cancer Research at MIT, MIT, Cambridge, MA USA; 3 Department of Pathology, Beth Israel Deaconess Medical Center and Harvard Medical School, Boston, MA USA; 4 Laboratory of Cellular and Structural Biology, The Rockefeller University, New York, NY USA

**Keywords:** Data-independent acquisition, Co-immunoprecipitation, Affinity enrichment, Label-free quantitation, Bioinformatics

## Abstract

Data-Independent Acquisition (DIA) LC–MS/MS is an attractive partner for co-immunoprecipitation (co-IP) and affinity proteomics in general. Reducing the variability of quantitation by DIA could increase the statistical contrast for detecting specific interactors versus what has been achieved in Data-Dependent Acquisition (DDA). By interrogating affinity proteomes featuring both DDA and DIA experiments, we sought to evaluate the spectral libraries, the missingness of protein quantity tables, and the CV of protein quantities in six studies representing three different instrument manufacturers. We examined four contemporary bioinformatics workflows for DIA: FragPipe, DIA-NN, Spectronaut, and MaxQuant. We determined that (1) identifying spectral libraries directly from DIA experiments works well enough that separate DDA experiments do not produce larger spectral libraries when given equivalent instrument time; (2) experiments involving mock pull-downs or IgG controls may feature such indistinct signals that contemporary software will struggle to quantify them; (3) measured CV values were well controlled by Spectronaut and DIA-NN (and FragPipe, which implements DIA-NN for the quantitation step); and (4) when FragPipe builds spectral libraries and quantifies proteins from DIA experiments rather than performing both operations in DDA experiments, the DIA route results in a larger number of proteins quantified without missing values as well as lower CV for measured protein quantities.

**Supplementary Information:**

The online version contains supplementary material available at 10.1007/s42485-024-00166-4.

## Introduction

In proteomics, Data-Independent Acquisition (DIA) is a method of mass spectrometry (MS) data acquisition that is designed to maximize the number of peptide ions that can be quantified. Effective implementations of DIA achieve more comprehensive coverage and quantitation than the mainstay MS acquisition method, Data-Dependent Acquisition (DDA). Early versions of DIA made their debut more than two decades ago (Purvine et al. 2003; Venable et al. 2004; Gillet et al. 2012), and this approach has become ubiquitous in MS-based proteomics.

DDA, the predecessor method to DIA, selects peptide ions that produce intense signals in MS (typically the “Top N” such peptide ions in a given MS scan), isolates many copies of a particular peptide ion, dissociates them to fragments, and then records the fragment ions in tandem mass spectra (MS/MS) (Tabb et al. 2010). DIA, on the other hand, collects tandem mass spectra according to a design, sampling windows of potential precursor ions (for example, in a window 20 m/z in width) in succession across a defined precursor range (for example 400 m/z to 1200 m/z). When the instrument completes a cycle of these MS/MS scans, the method typically captures a new MS scan and then starts the MS/MS cycle over again (Pino et al. 2020). The tandem mass spectra for DIA experiments differ substantially from those in DDA. Fragment ions are no longer associated with a particular precursor ion in the MS but rather the range of m/z for the DIA window. Each DIA MS/MS contains fragments from a mixture of peptides that have similar precursor m/z values and retention times. The fragment ions for each peptide are generally observed in multiple tandem mass spectra since the next cycle of measurement is intended to arrive well before the peptide has stopped eluting. This alteration of method shifts the experimental intent from “identify every peptide that produces an intense peak in MS” to “quantify every peptide that offers suitable MS or MS/MS chromatograms.”

The typical quantitation route for DDA relies on extracted ion chromatograms (XICs) that visualize the intensity observed for a precursor ion in the succession of MS scans during that peptide’s elution (Bubis et al. 2017; Smith and Tostengard 2020). The XIC may combine information across the isotopes of that peptide, and it may incorporate other charge states. In DDA, building chromatograms from fragment ion intensity is typically infeasible because the instrument control software attempts to avoid repeated MS/MS measurements of a given precursor ion (to broaden the diversity of precursors subjected to MS/MS). The “Match Between Runs” feature in quantitative software is intended to handle scenarios where a peptide is identified and quantified in one LC–MS/MS experiment, but it is not successfully matched to an MS/MS in another (Tyanova et al. 2016). The approach estimates the retention time at which that peptide might be expected to appear in the LC–MS/MS experiment where it was not identified, and the software attempts to find a precursor ion chromatogram from which a quantity can be inferred. Two potential errors can stand in the way of success. First, an interference can result if the chromatogram for peptide A is quantified for peptide B (Gallien et al. 2013); as peptide diversity and dynamic range grows for a sample, the chance of interference rises, contributing to a false quantitation rate (Lim et al. 2019). Second, a missing value can result if the software seeks a precursor ion chromatogram but is unable to find one in an LC–MS/MS (Webb-Robertson et al. 2015). DIA quantitation can reduce the chance of interference by estimating intensity on the basis of both precursor ion chromatograms and fragment ion chromatograms since the latter correspond to transitions from targeted quantitation (Lambert et al. 2013; Vidova and Spacil 2017). With DIA, interference at the fragment level can only happen if different peptides not only share precursor m/z and retention time but also produce overlapping sets of fragment ions. DIA quantitation can also improve on missingness in quantitation because for some peptides, fragment chromatograms are reliable when precursor chromatograms are not.

DIA may be most frequently featured in highly complex cellular extracts, but the technology also has value for affinity-enriched samples containing hundreds of proteins. In 2013, J-P Lambert and collaborators combined affinity purification with SWATH (an early implementation of DIA for SCIEX TripleTOF instruments) (Lambert et al. 2013). Pairing affinity purification with DIA revisits challenges of dynamic range, limiting sample quantities, and complex sample handling that have been observed in other applications of this technology. Mc Ardle et al. specialized their DIA method to quantify proteins in plasma or serum, accommodating the high dynamic range of these samples (Mc Ardle et al. 2022). Quantifying proteins available in vanishingly small quantities is quite typical of the single-cell proteomics application, and DIA has been successfully adapted for cells separated through fluorescence-activated cell sorting (Brunner et al. 2022). Because the complexity of sample handling creates many opportunities for variability to enter experiments, Lawrence et al. used spike-in peptides to evaluate this property in their DIA analysis of phosphorylation via IMAC column enrichment (Lawrence et al. 2016). Using DIA for affinity experiments has been made more tractable through lessons learned in other challenging environments.

Since affinity purification comes with the expectation of variability, it makes sense to try to limit variability in LC–MS/MS measurement and data analysis as much as possible (LaCava et al. 2015). In the early days of this technology, co-IP researchers might resort to “presence-and-absence” analysis to ask which prey proteins came down with each bait protein. Today it is far more common to compare quantities of a putative prey protein between genuine pull-downs and mock pull-downs. The power of statistical tests used to detect proteins that are more enriched in a genuine pull-down work best when the variability within each cohort is kept to a minimum. DIA-derived quantities have been observed to fluctuate less than those from DDA (Krasny and Huang 2021). Co-IP researchers can avoid compounding high variability inherent to affinity purification by combining co-IP with DIA rather than DDA methods.

This project evaluates the use of DIA with affinity purification, emphasizing experiments where ProteomeXchange contains both DDA and DIA experiments. It incorporates four different bioinformatics workflows for identification and quantitation and tests these systems by providing data from three different instrument manufacturers. FragPipe [https://fragpipe.nesvilab.org/], Spectronaut [https://biognosys.com/software/spectronaut/], DIA-NN [https://github.com/vdemichev/DiaNN], and MaxQuant [https://www.maxquant.org/] have all been equipped with the ability to identify and/or quantify these experiments. Some of the tools have previously been evaluated in the context of highly complex proteomes (Zhang et al. 2023). By comparing them in the context of affinity enrichment, the project will outline the strengths and weaknesses of each workflow in samples of a very different complexity.

## Methods

### Data sets

This bioinformatics evaluation incorporates both new experiments and previously published co-IP proteomes that include both DDA and DIA experiments for the same (or highly similar) samples. To seek more generalizable findings, the study encompassed data from instruments of three different vendors: Thermo Scientific, Bruker, and SCIEX (see Table 1 for an overview).

**Table 1:** Data sets from instruments of three different manufacturers examined different types of affinity enrichment

IP INPUTS	PXD054265: *M. musculus* gut extract		
Thermo Scientific Exploris 480			
Cys+46	Set A	Set C	Set G
DDA	21	24	27
HRMS1	21	24	26

SPION	PXD023278: *M. musculus* tissue extract and lysosomal enrichment		
Thermo Scientific Fusion Lumos			
Cys+71	LEF	LTL	MCWL
DDA	1	1	1
DIA	9	12	9

LINE-1	PXD054173: *H. sapiens* co-IP of LINE-1 ORF1p			
Thermo Scientific Exploris 480				
Cys+46	HEK293-IgG	HEK293-LINE1	N2102-IgG	N2102-LINE1
DDA	4	4	4	4
HRMS1	4	4	4	4
VV	4	4	4	4

RACK1	PXD002965: *D. melanogaster* co-IP of RACK1		
SCIEX TripleTOF 5600			
Cys+57	Control	RACK-cTerm	RACK-nTerm
DDA	6	6	6
SWATH	*5	6	6

ID4	PXD017517: *M. musculus* co-IP of ID4			
SCIEX TripleTOF 6600				
Cys+57	IgG-batch1	ID4-batch1	IgG-batch2	ID4-batch2
DDA	0	0	4	4
SWATH	3	3	3	3

DUBs	PXD031848: *H. sapiens* activity-based profiling of deubiquitylating enzymes					
Bruker timsTOF						
Cys+0	NoProbe	Titration	FT827	HBX108	P22078	PR619
dda-PASEF	4	5	4	6	6	12
dia-PASEF	4	5	4	6	6	12

HRMS1, VV, SWATH, and dia-PASEF are all types of DIA methods. The numbers in cells report the number of LC–MS/MS experiments in each cohort

*In RACK1, one Control SWATH experiment WIFF file was corrupted

### IP Inputs: Thermo Scientific Orbitrap Exploris 480

An initial Thermo data set represents the complex mixtures in which immunoprecipitation is typically performed. These tissue extracts are from the small intestine and colon in different feeding conditions. Of note, these gut extracts include epithelium, muscularis, and immune cells in the intestine as the starting material was intact tissue. To this end, dissected tissues were cut into small pieces, then Dounced in Phosphate buffered saline (PBS) including protease inhibitors (Roche 11,836,170,001) and phosphatase inhibitors (Roche 4,906,845,001). The soluble fraction was collected for further analysis. Three independent datasets were generated: Dataset ‘A’ includes 21 DDA experiments and 21 DIA experiments, Dataset ‘C’ includes 24 DDA and 24 DIA experiments, and ‘G’ includes 27 DDA RAWs and 26 DIA RAWs (one of the ‘G’ DDA experiments was run twice). Datasets ‘A’ and ‘G’ were intended to compare the whole intestine proteome in different feeding conditions, namely *ad libitum* feeding, 24 h fasted, and 24 h fasted, then re-fed for 4 h; Experiment ‘C’ was to compare the whole intestine proteome between germ-free condition and specific pathogen-free (SPF) states. In each case, these RAWs represent the input starting material for further immunoprecipitation analysis that will be described elsewhere. Sample preparation was performed using the S-TRAP method (Zougman et al. 2014), with MMTS (methyl methanethiosulfonate) alkalyting free Cys side chains. DDA experiments collected spectra for all 120 min of a two-hour run, while DIA experiments using the high-resolution MS1 (HRMS1) technique (Xuan et al. 2020) collected scans at two different FAIMS compensation voltages for 75 min during each 105 min run, with scan events ending when the “B” solvent of the gradient reached 45%. Supplementary Table 1 supplies the file names for each subset of each data set. The sequence database for the IP Inputs set contained the UniProt reference proteome for mouse including isoforms (UP000000589: 63,191 sequences) as well as the human sequence for TMEM192 (Q8IY95). These data are available as PXD054265.

### SPION: Thermo Scientific Orbitrap Fusion Lumos

Peter Mosen et al. compared the performance of parallel reaction monitoring (PRM) and DIA methods for quantifying lysosomal components in *Mus musculus* (Mosen et al. 2021). Their enrichment employed superparamagnetic iron oxide nanoparticles (SPIONs) rather than immunoprecipitation. The set is also distinctive by incorporating multiple gradient lengths: 60, 120, and 240 min (120, 180, and 300 min total time for scan collection, respectively). Three cohorts comprise the study, a lysosome-enriched fraction (LEF), mouse whole cell lysate (MWCL), and a liver tissue lysate (LTL). A few experiments spiked LEF into LTL; we included these among the LTL experiments. Each was subjected to DDA (a single 240-min gradient), DIA (triplicates of each of the three gradients), and PRM (triplicates of two 60-min gradients and triplicates of 120-min gradients). For this study, acrylamide propionamidylated the cysteines (+ 71 Da). The sequence database for the SPION set contained the UniProt reference proteome for mouse including isoforms (UP000000589: 63,191 sequences), the human sequence for TMEM192 (Q8IY95), and the Biognosys iRT sequence. These data are available as PXD023278.

### LINE-1: Thermo Scientific Orbitrap Exploris 480

In the course of this project, the authors developed a variable window instrument method (called “Variabele Vensters” or “VV”) to sample MS/MS fragment chromatograms far more frequently than was possible in the “HRMS1” technique used in the IP Inputs experiments. Using an anti-ORF1 monoclonal (clone 4H1; Millipore), four co-IPs to enrich for LINE-1 ORF1p and its interactors from either HEK-293T_LD_ (as described in (García Pérez 2016)), using plasmid-based forced ectopic expression, or N2102Ep cells (embryonal carcinoma cells that exhibit steady-state endogenous expression of LINE-1; as described in (Di Stefano et al. 2023)). Elutions from the IPs were then analyzed in DDA, HRMS1, and VV modes. Mouse polyclonal IgG were used as mock IPs as nonspecific binding controls. As in the Lyso-IP Inputs, the S-TRAP protocol was employed, with MMTS alkylating the cysteines. DDA and VV experiments collected spectra for 60 min of the one-hour run time. The HRMS1 experiments collected spectra for 90 min of a two-hour run time. The UP000005640 reference proteome for *Homo sapiens*, including unreviewed sequences and reviewed isoforms, was used as a search space (104,558 sequences), with the Biognosys iRT sequence added (for use in a set below). These data are available as PXD054173.

### RACK1: SCIEX TripleTOF 5600

Lauriane Kuhn et al. investigated the protein–protein interactions of the Receptor for Activated Protein C Kinase 1 (RACK1) in *Drosophila melanogaster* S2 cells in their 2017 paper (Kuhn et al. 2017). Their study is split into three cohorts. The Control set used an anti-FLAG antibody on cells in which the transfected RACK1 incorporated a hemagglutinin tag (six DDA and six DIA experiments, with one DIA file garbled in ProteomeXchange). The N-terminal cohort positioned the FLAG tag at the N-terminus of the transfected RACK1 (six DDA and six DIA experiments), and the C-terminal cohort positioned the FLAG tag at the C-terminus of the transfected RACK1 (six DDA and six DIA experiments). For this study, iodoacetamide carbamidomethylated the cysteines (+ 57 Da). The sequence database for the RACK1 set contained the UniProt reference proteome for *D. melanogaster* including isoforms (UP000000803: 23,543 sequences) plus three viral sequences (O36967, P13418, and Q9IJX4). These data are available as PXD002965.

### ID4: SCIEX TripleTOF 6600

Holly Holliday et al. characterized proteins interacting with Inhibitor of Differentiation 4 (ID4) in mammary basal stem cells of *Mus musculus* (Holliday et al. 2021). We employed the ID4 and IgG control pull-down data, combining differentiated and undifferentiated data into six cohorts: DDA for ID4, DDA for IgG, DIA for ID4 (two distinct batches), and DIA for IgG (two distinct batches). The DDA sets contained four LC–MS/MS experiments while the DIA sets each contained triplicates. The second batches for the DIA experiments were collected six months later than the first batches, but all employed SWATH methods spanning 80 min. For this study, iodoacetamide carbamidomethylated the cysteines (+ 57 Da). The sequence database employed was the same as for the “IP Inputs” set above. These data are available as PXD017517.

### DUBs: Bruker timsTOF

Hannah B. L. Jones et al. employed activity-based profiling to find human deubiquitylating enzymes (DUBs) that could be precipitated by a set of DUB enzyme inhibitors (Jones et al. 2022). Their study incorporates cohorts for four different inhibitors: FT827 (four DDA and four DIA experiments), HBX108 (six DDA and six DIA experiments). P22078 (six DDA and six DIA experiments), and PR619 (12 DDA and 12 DIA experiments). It also includes two technical sets: “titration” (five DDA and five DIA experiments) and “no probe” (four DDA and four DIA experiments). For this study, no alkylation of cysteines took place. The human sequence database described above for the LINE-1 set was re-used for the DUBs data. These data are available as PXD031848; we have reposted the data as MSV000095426 at massive.ucsd.edu to ease the download of individual experiments.

### Bioinformatics for spectral library creation and DIA quantitation

**Fig. 1:**
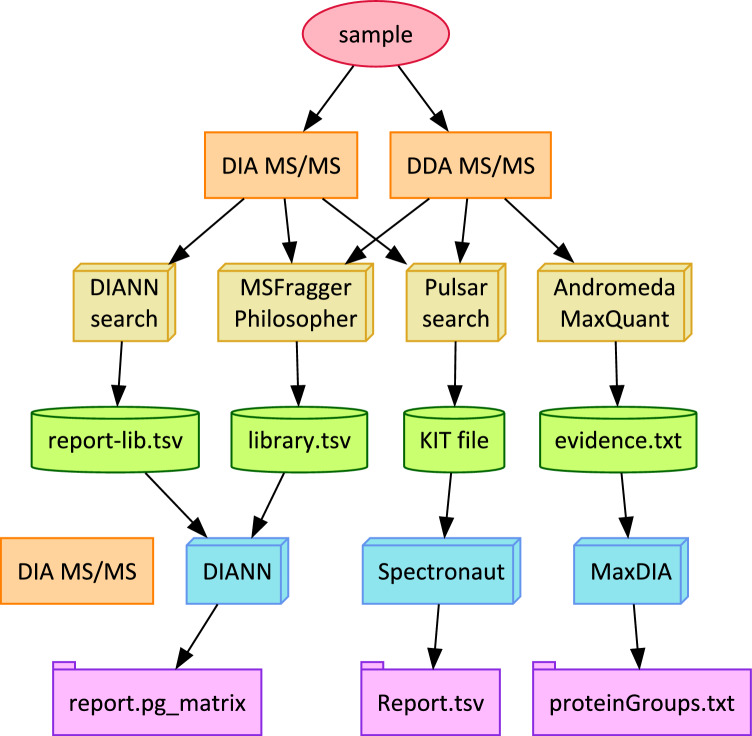
Four different DIA identification and quantitation workflows were evaluated: DIA-NN, FragPipe, Spectronaut, and MaxQuant (from left to right). Each produced a spectral library in a different format (green cylinders). Each reported protein quantity matrices to text tables (violet folders), with FragPipe using the DIA-NN quantitation engine. Whether spectral libraries were derived from DDA or DIA experiments, these libraries were used to quantify the corresponding DIA experiments

Four software frameworks were evaluated for their ability to produce spectral libraries from DDA or DIA experiments and then apply them for quantification in DIA experiments: FragPipe 21.1, DIA-NN 1.8.2 beta 8, Biognosys Spectronaut 18.4, and MaxQuant 2.5.0.0 (see Fig. 1).

### FragPipe 21.1 with MSFragger and DIA-NN

The FragPipe workflow manager coordinates the use of the MSFragger search engine 4.0 (which is capable of identifying either DDA or DIA MS/MS directly) (Yu et al. 2023), the Philosopher 5.1 suite of peptide-spectrum match validation and protein inference tools, and the DIA-NN 1.8.2 quantification engine. The FragPipe implementation differs from using DIA-NN alone in that its spectral library is compiled from MSFragger identifications rather than those of the DIA-NN search engine. Use of FragPipe centered on its “DIA_SpecLib_Quant” workflow, though the generation of these libraries was often separated from their use in quantitation; during spectral library creation, the MSFragger, Validation, and “Spec Lib” tabs were included in execution, and then during quantitation only the “Quant (DIA)” tab was executed. The connection between identification and quantitation experiments was the “library.tsv” file that contains the spectral library from an identification experiment and which governs which fragment ions should be sought to quantify each precursor ion (i.e. the transitions). Philosopher added common contaminant sequences to the FASTA and added each FASTA sequence in a reversed orientation to estimate FDRs empirically.

Generally, default values were used for processing. Data set-specific configurations included the following:

- The IP Inputs, LINE-1, SPION, and DUBs data sets required customization for the static modification of Cys (UniMod 39: + 46 for IP Inputs / LINE-1, UniMod 24: + 71 for SPION, and + 0 for DUBs).
- FragPipe cannot read SCIEX WIFFs directly. For the DDA experiments, it was provided with mzMLs created in SCIEX MS Data Converter 1.3.1 using “ProteinPilot” peaklisting. For the DIA experiments, the mzMLs were created in ProteoWizard msConvert.
- As of version 21.1, FragPipe is unable to identify Bruker diaPASEF MS/MS scans directly; as a result, the only spectral libraries produced for the DUBs data set in FragPipe were created from DDA experiments.
- For DIA-NN quantitation, the IP Inputs, LINE-1, and SPION data required this string in the GUI-configured command line options: “–mod UniMod:39,45.987721” (or “–mod UniMod:24,71.037114” for SPION) to recognize alkylation methods other than iodoacetamide in the spectral library.

### Standalone DIA-NN 1.8.2 *beta* 8

The DIA-NN (Demichev et al. 2020) included in FragPipe 21.1 can also be used independently of FragPipe via its convenient GUI. The software was able to read Thermo RAW files via MSFileReader and Bruker.d; by copying libraries distributed with ProteoWizard, it can also read WIFF files. In all cases, however, the DIA-NN search engine is intended only for DIA data, so it was not applied to build libraries from DDA experiments in any of the four experimental data sets. The software’s near-constant use of all available CPU threads was distinctive among the software workflows employed here, requiring hours to complete analysis of Bruker DUBs sets even on a 32-core AMD 5975WX CPU. In distinction to the FragPipe searches, DIA-NN was supplied with the UniProt FASTA database with manual additions but without contaminant sequences or appended decoys (the software uses internal methods for controlling FDR). For the first run on a data set, the FASTA digester was enabled with “Deep learning-based spectra.” Oxidation of Met and N-terminal acetylation was added to provide a comparable search space with other search engines. Subsequent runs on a data set used the spectral library produced in the initial FASTA digestion run to save computing time. The spectral libraries were reported to report-lib.tsv files, containing similar information to those produced by FragPipe, though with different column headings.

DIA-NN identification and quantitation in the IP Inputs and LINE-1 data required these strings under “Additional options” to incorporate the MMTS modifications of Cys (the SPION set changed these lines to reflect UniMod accession 24 instead):

- fixed-mod UniMod:39,45.987721,C
- mod UniMod:39,45.987721.
- strip-unknown-mods.

### Spectronaut 18.4 and the Pulsar search engine

The commercial software Spectronaut (Bruderer et al. 2015) has been a popular choice for DIA proteomics, owing to its versatile options and user-friendly features. Creating a spectral library from raw data and a FASTA database began with the “Library” tab in the GUI, specifically the “Generate Library from Pulsar / Search Archives” option. Modifications to Cys were specified in Pulsar Search Settings under Modifications, leaving the default PTMs of Met oxidation and protein N-terminal acetylation in place. Producing an “AllPeptides.tsv” file from a KIT library was available via the “Export Peptide List” option from the context menu. Quantifying DIA experiments with these KIT libraries employed the “Set up a DIA Analysis from File” option on the “Analysis” tab in the GUI. The “Assign Spectral Library” option selects the KIT to be applied during quantitation.

- The IP Inputs, LINE-1, SPION, and DUBs data sets required customization for the static modification of Cys (UniMod 39: + 46 for IP Inputs / LINE-1, UniMod 24: + 71 for SPION, and + 0 for DUBs). The UniMod 24 mass shift for Cys required editing of the available modifications in the “Databases” tab of the GUI.
- The IP Inputs and LINE-1 HRMS1 methods emphasize MS1 acquisition over repeated measurements of fragments, so for Spectronaut the “Quantification” setting “Quantity MS Level” was set to “MS1” for these experiments.

### MaxQuant 2.5.0.0 and the MaxDIA quantitation engine

The widely-used MaxQuant search engine incorporates the MaxDIA quantitation engine (Cox and Mann 2008; Sinitcyn et al. 2021), enabling it to build spectral libraries from DDA experiments that are then quantified in DIA experiments. Where possible, the engine was used in conjunction with a RAM Drive created by the ImDisk Toolkit (https://sourceforge.net/projects/imdisk-toolkit/) or an SSD to reduce delays related to reading and writing temporary files. In all cases, MaxQuant worked from raw data formats for identification. MaxQuant is only able to identify MS/MS in DDA mode; when used to identify DIA MS/MS, its libraries are incomparably smaller. Consequently only its DDA-derived libraries are included here. The output files evidence.txt and msms.txt define the spectral library to be used by MaxDIA, and the search FASTA must also be provided for quantitation. MaxQuant was supplied with versions of the sequence databases that contained only the reference proteomes and manually added proteins; contaminants and decoy sequences were added by MaxQuant automatically.

- The IP Inputs and LINE-1 experiments required the addition of MMTS methylthio Cys alkylation (UniMod 39) to the modifications.xml. The same was necessary for UniMod 24 in SPION.
- In Thermo experiments, MaxQuant Group-Specific Parameters Type was set to “MaxDIA.” In Bruker DUBs experiments, the Type parameter was set to “TIMS-MaxDIA.”
- For the SCIEX TripleTOF sets, MaxDIA was unable to quantify peptides due to an error in the Feature Detector using either the WIFF SWATH data or mzML files produced from those WIFFs by ProteoWizard.

### Overlap and reproducibility analysis

Scripts for the R Statistical Environment (Gatto and Christoforou 2014) were created for the examination of spectral libraries and of per-protein quantity tables reported by each software pipeline. A “Read” function for each workflow parsed the exported text versions of spectral libraries: report-lib.tsv for DIA-NN, evidence.txt for MaxQuant, library.tsv for FragPipe, and AllPeptides.txt for Spectronaut. A “Quant” function for each workflow parsed the protein quantity tables: report.pg_matrix for DIA-NN and FragPipe, proteinGroups.txt for MaxQuant, and Report.tsv for Spectronaut. The DiversityStats function evaluated spectral libraries for numbers of distinct genes, proteins, peptides, modified peptides, and “MPZs” (numbers of peptides with a given modification set at a particular charge state). The UpSetR library (Conway et al. 2017) visualized intersection analysis for peptides appearing in spectral libraries. CVbyRow and Quantity Stats functions evaluated the variability of protein quantities as well as the extent of missingness in these tables. The script also includes “fpMBRIntensity” and “fpMBRMaxLFQIntensity” functions for reading quantity tables from FragPipe IonQuant assessments of DDA experiments. It includes a section of code to derive variable window boundaries to create DIA methods based on the IP Inputs A experiment. The scripts, their inputs, and their outputs can be found in Supplementary File 1; the script is described in Supplementary Text 1.

## Results

Evaluating the value proposition of DIA to replace DDA for co-IP requires data sets where both types of experiments were performed. At the time of writing, the ProteomeXchange repository (Deutsch et al. 2023) offers a host of co-IP data sets for Thermo instruments, but a majority of these PXD entries contain DDA experiments only. Seeking ProteomeXchange Bruker and SCIEX QqTOF co-IP experiments where both DDA and DIA were included produced even fewer options. The sets included here will begin with two complex samples: mouse digestive tissue lysates intended as an input to immunoprecipitation and nanoparticle-enriched lysosomal proteins (“Thermo SPION”). It will move from there to a variety of immunoprecipitations: co-IP experiments in human cell lines (“Thermo LINE-1”), fruit fly Schneider 2 cells (“SCIEX RACK1”), murine basal cells (“SCIEX ID4”), and human MCF-7 mammary epithelium cells (“Bruker DUBs”). Having both DDA and DIA data for these experiments enables a direct comparison of spectral libraries derived from DDA database search (via FragPipe, MaxQuant, or Spectronaut) or DIA database search (via FragPipe, DIA-NN, or Spectronaut). Quantifying the peptides of the spectral libraries can then be carried out in the DIA experiments (via all of these algorithms).

### Spectrum library diversity from DDA or DIA database search

We framed two hypotheses before examining the spectral libraries derived from these experiments:

(A) Subsets of peptides are most likely to be identified by a particular identification algorithm.
(B) Subsets of peptides are more likely to be identified in DDA than in DIA, or vice versa.

A given peptide might have a greater chance of identification by one search engine rather than another because the software embeds a fragmentation model that better predicts the fragments to be seen in an MS/MS of the peptide (C. Silva et al. 2019). If the same fragmentation model is in operation whether that search engine is operating on DIA or DDA experiments, the advantage in identifying this peptide would apply in both cases. This phenomenon can be evaluated in FragPipe since MSFragger can manage either DDA or DIA identification, and in Spectronaut, where the Pulsar search engine can manage either experiment type. Only these two software workflows of the four we examined are designed to identify directly both DIA and DDA experiments.

Since the creation of DIA instrument methods, many in the proteomics community have assumed that the best spectral library for a sample type must be created from DDA experiments. Because DDA produces an MS/MS of fragments from an isolated peptide, recognition of that peptide by a search engine is more likely. This assumption, however, ignores the advantages of DIA for identification. First, DIA produces redundant fragment measurements for each peptide; multiple MS/MS scans enumerate fragments for each peptide, increasing the information upon which identification may be based. Second, DIA provides fragment information from a greater diversity of peptides by multiplexing the MS/MS process. In DIA, a peptide is no longer dependent on producing an intense MS signal for its fragments to be measured. In the last several years, search engines designed to take advantage of these features have greatly improved the identification yield from DIA experiments (Pino et al. 2020).

### Spectral libraries from IP inputs and SPION lysosome enrichment

The IP Inputs data set represented mouse gut extracts without antibody enrichment, and each subset experiment represented many replicates (21 Thermo RAWs for “A”, 24 for “C”, and 26 for “G”). The DDA sets held the advantage of LC–MS/MS experiments collecting spectra for 120 min, while the DIA experiments each collected spectra for 75 min (Quality metrics for all raw data appear in “Basic Quality Metrics,” Supplementary File 1). All six of the searches (DIANN-DIA, FragPipe-DDA, FragPipe-DIA, MaxQuant-DDA, Spectronaut-DDA, and Spectronaut-DIA) yielded substantial collections of peptides in their spectral libraries, from a low of 20,108 distinct peptide sequences in MaxQuant on the “C” experiments to a high of 57,677 distinct peptide sequences in Spectronaut using DIA data from the “G” experiments (see Supplementary Table 2 for a comparison of all spectral libraries for all algorithms in each experiment). We used the number of distinct peptide sequences in a library as its “diversity” because these values could be compared directly from the different search engine spectral library formats (represented as green cylinders in Fig. 1).

The peptide diversities of DIA-derived libraries mount a challenge to the assumption that DDA experiments are necessary to create spectral libraries for the quantitation of DIA experiments. FragPipe produced more diverse spectral libraries for DDA than DIA in IP Inputs experiments “A” and “C”, but its DIA library was more diverse than its DDA library in an experiment “G”. For Spectronaut, the DIA experiments led to more diverse libraries in “C” and “G”, but the DDA experiments for “A” gave more diverse libraries in Spectronaut. This seems like an equivocal result until instrument time is factored into the assessment. The DDA experiments required 60% more instrument acquisition time per LC–MS/MS run than did the DIA experiments, but they did not yield more diverse spectral libraries.

**Fig. 2:**
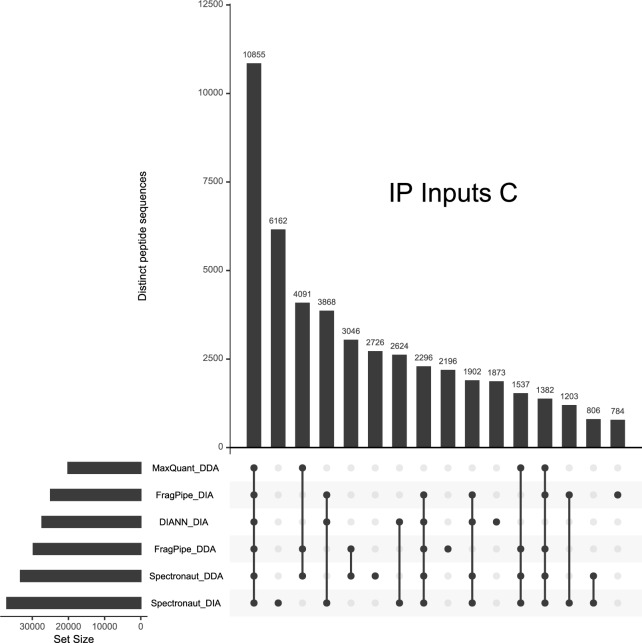
This UpSet plot reveals how the six spectral libraries derived from IP Inputs “C” overlap in the peptide sequences they represent. The bars at the lower left represent the numbers of distinct peptide sequences in each library. The dots on the lines below the main graph specify which spectral libraries contain the peptides for a particular intersection of libraries. The single dot under the second bar (“6162”) indicates that these peptides were identified only by the Spectronaut search against DIA data. The sizes of the bars in the main graph represent the number of peptide sequences in each intersection

The intersections of these spectral libraries indicate which peptide sequences were shared among different combinations of these six spectral libraries for each of the three IP Input experiments. As shown in Fig. 2 for experiment “C”, the largest intersection set of peptides among these spectral libraries (10,855 peptide sequences) was universally identified among all six searches. Figure 2 illustrates a common trend among the experiments in that peptide sequences frequently are associated with only DDA libraries (4091 peptide sequences) or only DIA libraries (3868 peptide sequences). This common feature among the UpSet plots reinforces hypothesis B from the head of this section: some sets of peptide sequences have a propensity to being identified in either DIA or DDA experiments. Although the project did not investigate peptide-level intensities, it is likely that consistently identified peptides are also among the more intense signals in these experiments (Tabb et al. 2010). The UpSet plots for all experiments can be found in Supplementary Fig. 1.

The SPION experiments enriched lysosomes from mouse tissue using nanoparticles; as a result, the enriched samples contain far greater peptide diversity than would be typical of a co-IP. The DIA methods employed in SPION varied in duration from 120 to 300 min, and they emphasized MS/MS data for quantitation rather than relying on MS scans as does the “High-Resolution MS1” method employed for the IP Inputs. The long SPION LC gradients acquired nearly 200,000 MS/MS scans per RAW. DDA was only lightly used in SPION, collecting only a single 240-min RAW for each of the three sub-experiments: Lysosome-Enriched Fraction, Liver Tissue Lysates, and Mouse Whole Cell Lysates.

The IP Inputs and SPION sets both show DIA-NN and Spectronaut producing very diverse spectral libraries from DIA experiments. Spectronaut libraries were more diverse than DIA-NN libraries in the IP Inputs, while SPION showed DIA-NN having the advantage over Spectronaut. Despite coming from a nano-particle enrichment rather than a lysate, the SPION experiments produced more diverse libraries than the IP Inputs, reaching a high of 135,775 distinct peptide sequences (DIA-NN in the SPION Mouse Whole Cell Lysates). Comparing the sizes of these libraries between different algorithms may ignore subtly different search spaces and different strategies for downstream peptide-spectrum match filtering. At the early stages of this project, the very large DIA libraries produced by Spectronaut were viewed with some doubt by the authors, but subsequent confirmation of many of these additional peptides by use of DIA-NN alleviated much of this skepticism. The peptide diversities of spectral libraries for IP Inputs and SPION from MaxQuant were consistently lower than for other algorithms, even considering only those produced from DDA data.

### Spectral libraries from co-immunoprecipitation

After the highly diverse spectral libraries of IP Inputs and SPION, the antibody-enriched spectral libraries seem very compact, with only the Bruker timsTOF DUBs set yielding any libraries above 10,000 distinct peptide sequences. This reduction of scale brought with it larger variability and greater prominence to contaminant proteins.

Handling of contaminant proteins is uneven among these four search engines (Frankenfield et al. 2022). For FragPipe (whether operated in DDA or DIA data), the FASTA database provided should already include contaminants, and each target sequence should be matched by a decoy. The FragPipe “Add decoys” button handled both tasks for this project. MaxQuant also adds contaminants and decoys, but these actions take place internally based on an uncontaminated target-only database provided by the user; its contaminants overlap with those of FragPipe, but many sequences are specific to each contaminant set. Spectronaut and DIA-NN do not appear to add contaminants, and they handle FDR filtering internally without explicit decoys.

The low peptide mass resulting from co-IP may cause bait and prey proteins to be supported by less peptide evidence than mass spectrometry-friendly contaminant proteins (here “contaminant” implies the protein is likely to have resulted from sample handling rather than to have non-specifically interacted with the antibody (Mellacheruvu et al. 2013)). For the SCIEX RACK1 *D. melanogaster* data set, for example, the protein with the largest number of distinct peptides in all FragPipe and MaxQuant libraries was P04264 (human keratin, type II cytoskeletal 1). Because Spectronaut and DIA-NN lacked contaminant sequences, they typically reported C7LA75 / P11147 (heat shock 70 kDa protein cognate 4) or P08736 (elongation factor 1-alpha 1) from *D. melanogaster* as the top hit instead because the *D. melanogaster* reference proteome does not contain human keratins. As in the DDA database search, if the FASTA does not contain a protein sequence, DIA will not be able to identify or quantify it.

The RACK1 set was not alone in having prominent contaminant peptides. In the SCIEX ID4 *M. musculus* set, the first batch of DIA experiments delivered lower sensitivity than the second batch; as a result, P13645 (human keratin, type I cytoskeletal 10) accounted for the largest number of peptides in the ID4 pull-down spectral libraries for this batch. The top hits of the Bruker DUBs *H. sapiens* set appear to be the deubiquitylating enzymes targeted by their experiments. The co-IP data for LINE-1 ORF1p in *H. sapiens* cell lines introduced with this project, however, identified a range of keratins in both the mock co-IP controls and genuine co-IPs of the target protein: P04264, P05783, P05787, P13645, P35527, and P35908. The contaminants accompany rather than mask the abundant proteins of interest that interact with the LINE-1 ORF1p target. If researchers perform searches of non-human databases, they will want to ensure that the sequence database provided to Spectronaut and DIA-NN contains common human contaminants, but the tools do not also require decoy sequences to be added.

**Fig. 3:**
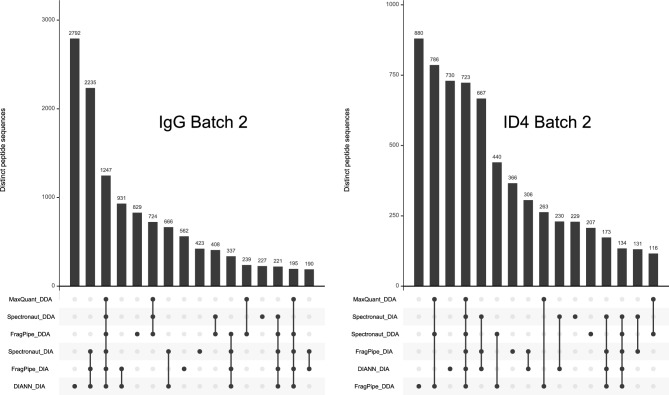
Batch 2 of the ID4 and IgG control pull-downs on the SCIEX 6600 produced spectral libraries that were far more diverse than in batch 1. For WIFFs representing the ID4 co-IP (right), the DDA experiments identified more distinct peptides than the DIA experiments. The IgG control (left), however, yielded better peptide diversity for the DIA experiments than for the DDAs

The RACK1 and ID4 experiments represent the performance of SCIEX “TripleTOF” instruments for co-IP. RACK1 (collected on a TripleTOF 5600) started with a positive outcome for MaxQuant, producing diverse spectral libraries for the “C-term” and “Ctrl” subsets, but attempts to use those libraries to quantify the DIA WIFF files in MaxDIA resulted in errors and no output tables. MaxDIA produced similar errors from the ID4 experiment, which employed the newer TripleTOF 6600. Because WIFF files are not supported natively in FragPipe, the DDA WIFFs were changed to mzMLs with the AB Sciex MS Data Converter [http://www.absciex.com/downloads/software-downloads] while SWATH WIFFs were converted to mzMLs in ProteoWizard msConvert. The SCIEX ID4 set is notable for including DIA experiments collected in two distinct batches, about three months apart, leading to a prominent batch effect (Čuklina et al. 2021). The second set of experiments produced far more diverse spectral libraries than did the first, with inventories growing by a factor of 3.8 × to 7.1× (Fig. 3 shows overlaps within the second batch). This identification impact may reflect the variability of immunoprecipitation and/or instrument performance.

While most experiments appeared to offer similar sensitivity of identification between DIA and DDA experiments, the Bruker timsTOF DUBs experiments were a substantial exception. In all four inhibitors as well as the negative control and titration experiments, the DIA-derived spectral libraries were far more diverse in peptides than were the DDA-derived libraries (see Supplementary Table 2F and Supplementary Fig. 1F). FragPipe 21.1, used for this project, does not yet support direct identification of diaPASEF experiments (Meier et al. 2020), and so only DIA-NN and Spectronaut were able to take advantage of the high-quality DIA experiments for much larger spectral libraries. The set demonstrates that being able to identify DIA spectra directly can offer a considerable advantage in the set of peptides available for quantitation.

### Sensitivity, missingness, and reproducibility for quantity tables

It is certainly possible that software could identify a peptide from MS/MS data without being able to quantify it. Most DIA software workflows distinguish between no viable chromatogram (resulting in a missing value or a reported 0 for a given precursor ion) and detected chromatograms (positive intensity is recorded). The tables that are most valued from DIA searches typically are those that report quantities for proteins, which requires some type of summarization for the precursor ion chromatograms that often take into account which peptide sequences are specific to a particular protein.

This project evaluated the protein quantity tables on a variety of bases. First, we compared the number of proteins identified from a set of experiments to the number of proteins with any quantity reported. Second, we compared the number of proteins quantified in any experiment to the number of proteins identified in every experiment (no missing values). Because the intensity recorded for a protein bears a strong relationship to the variability of measurement (Oberg and Mahoney 2012), we separated the proteins with no missing values into five categories based on summed intensity. We could then compare the coefficient of variations for high-intensity, middle-intensity, and low-intensity proteins. With this analysis in place, it becomes possible to compare the missingness and reproducibility of quantitation between DIA and DDA experiments for selected datasets.

**Fig. 4:**
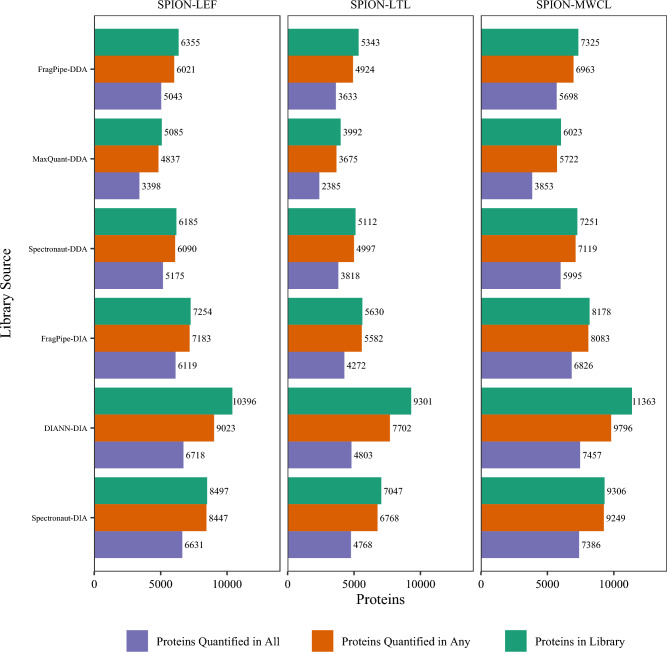
The nanoparticle-enriched experiments for lysosomal proteomics illustrate the distinction among the numbers of proteins identified in the spectral library, the numbers of proteins that have at least one quantitative value reported, and the numbers of proteins that have all quantitative values reported / no missing values. All these quantitative statistics reflect the application of spectral libraries to quantify DIA experiments, whether the “Library Source” was DDA or DIA

The nanoparticle-enriched (SPION) lysosome data illustrate the disparity between identified and quantified proteins (Fig. 4). DIA-NN identified a spectral library of 10,396 distinct proteins among the nine DIA RAWs for the LEF (lysosome-enriched fraction) cohort. The software reported quantities for 9023 of these proteins (87% of identified proteins). Of the 9023 quantified proteins, only 6718 (74%) had quantities reported for all nine experiments. This fraction is driven downward by the fact that three of the RAWs spanned 300 min, three spanned 180 min, and three spanned 120 min (labeled 240, 120, and 60 in filenames to represent the shallow gradient duration). As expected, the number of missing values for the shortest duration experiments (mean of 1615) is higher than for the middle duration experiments (mean of 568), and the middle duration experiments have more missing values than the longest duration experiments (mean of 168).

Co-IP for protein–protein interactions typically yields hundreds of proteins rather than thousands, and these diversities dip lower for negative controls. Mock immunoprecipitations and other types of negative controls may vary substantially from positive pull-downs in the sets of proteins they sample (Moresco et al. 2010) (see Supplementary Fig. 4). These sparse LC–MS/MS data create quite a challenge for these software workflows. In LINE-1 experiments, FragPipe failed to quantify any proteins in its output tables for the IgG control HRMS1 data, perhaps due to the low peptide concentration of these controls. DIANN occasionally warned that its machine learning models for retention times had too few peptides for proper training. Spectronaut frequently warned of too few peptides when quantifying in negative control samples. MaxDIA depends heavily upon MS2 chromatograms, damaging its quantitation performance in the HRMS1 experiments of the IP Inputs and of the LINE-1 co-IPs (see Supplementary Table 3 and Supplementary Text 2). The MaxQuant pipeline was unable to quantify the RACK1 and ID4 experiments because MaxDIA produced no output when presented with WIFF files (or their mzML equivalents). Co-IP experiments often rely upon comparing the intensities of proteins in a positive pull-down to their intensities in a negative pull-down, but the latter category of sample is exactly where quantification software struggles most.

Coefficients of Variation (CVs) are computed by dividing the standard deviation of expression by the mean of expression. The R scripts created for this project winnowed out sets of “unanimous” proteins for which no missing values were reported among all experiments of a cohort. These sets were then separated into quintiles based on the sum of intensity reported for each protein. We expected that the quintile of highest-intensity proteins would have a smaller median CV of quantity than the mid-intensity proteins and that the mid-intensity proteins would have a smaller median CV of quantity than the lowest-intensity proteins.

The protein quantity tables created by MaxDIA contained substantially higher CVs than the tables reported by other quantitation engines (see Supplementary Table 3 and Supplementary Fig. 3), so they were omitted from consideration of the intensity-CV relationship. Each quantitation of each cohort in each data set “voted” a TRUE if the CV values conformed to the hypothesis that highest-intensity proteins would have a lower CV than mid-intensity proteins and mid-intensity proteins had lower CVs than lowest-intensity proteins, or a FALSE if both these conditions were not met. If the CV values had no relationship to intensity, we would expect one in six data sets to randomly vote “TRUE.” The three Thermo data sets (IP Inputs, SPION, and LINE-1) produced 45 cases where the CVs were ranked as expected and 12 cases where they did not. The two SCIEX data sets (RACK1 and ID4) were also generally in agreement, giving 25 cases where the CVs were ranked as expected and 10 cases where they did not. Finally, the Bruker data set (DUBs) produced 15 cases where the CVs were ranked as expected and 9 where they were not. The IP Inputs and SPION data sets were considerably more diverse proteomes than the others, and only one quantitation effort of 27 (FragPipe using a DIA library on the “C” cohort of the IP Inputs) did not yield CVs in the rank order expected. It is possible that the greatly decreased proteomic diversity of co-IP experiments can also disrupt expected relationships between intensity and variance.

Spectronaut software offers a “library-free” quantification method named “directDIA.” In the IP Inputs G set, we tested the directDIA method from Spectronaut 19 versus the DIA-derived spectral library method in Spectronaut 18. The directDIA method quantified 6% more proteins in IP Inputs G, and the number of proteins quantified unanimously across all RAW files climbed by 4%. The CV values were slightly lower for directDIA high-intensity, mid-intensity, and low-intensity proteins. These minor differences may have resulted from the use of directDIA rather than a DIA-derived spectral library, or they may have resulted from changes in the more recent version of Spectronaut. In either case, it appears that the characterization of DIA-derived spectral libraries in Spectronaut can approximate expected directDIA performance.

### Adjusting lab practices in response

For the past few years, most DIA experiments on Thermo instruments at the Interfaculty Mass Spectrometry Center of UMCG have made use of the High-Resolution MS1 technique (Xuan et al. 2020), introduced in Thermo training workshops and evaluated through a variety of tests at UMCG. HRMS1 infers peptide intensities from peptide ion chromatograms rather than fragment ion chromatograms, ensuring a high rate of MS acquisition interspersed among the cycles of MS/MS windows. The specific variant of HRMS1 employed in the IP Inputs set included two FAIMS compensation voltages (CVs) and 88 MS/MS windows, and so software that incorporates fragment ion chromatograms alongside peptide ion chromatograms would find relatively few samplings of the fragments for a given peptide for this data set.

We sought to develop the “Variabele Vensters” (variable windows) method to boost the chromatographic resolution for fragment ions, using the 21 LC–MS/MS experiments of the IP Inputs Set “A” to represent a diverse proteome. We decided to include 30 windows in each cycle based on an estimated chromatographic peak width of 30 s and an MS/MS acquisition rate of 10 Hz. If each cycle of windows is collected in three seconds, a typical fragment chromatogram would be sampled in MS/MS ten times. We computed theoretical window boundaries that separated the peptide precursor m/z values in the IP Inputs Set “A” spectral library to 30 equal parts. Each of these theoretical window boundaries were rounded down (to give the next window start value) or rounded up (to give the previous window stop value) (See Table 2). This rounding gave two advantages: entering the method into the instrument control software was less error-prone, and successive windows overlapped by one m/z.

**Table 2:** The Variable Vensters DIA method attempts to make each window the same number of identifiable peptide ions in width, using IP Inputs A DDA as a guide

Window	Start m/z	Stop m/z	Width
1	374	396	22
2	395	413	18
3	412	428	16
4	427	443	16
5	442	456	14
6	455	470	15
7	469	484	15
8	483	498	15
9	497	512	15
10	511	526	15
11	525	541	16
12	540	555	15
13	554	570	16
14	569	585	16
15	584	601	17
16	600	617	17
17	616	634	18
18	633	652	19
19	651	670	19
20	669	689	20
21	688	710	22
22	709	731	22
23	730	755	25
24	754	780	26
25	779	806	27
26	805	834	29
27	833	864	31
28	863	901	38
29	900	945	45
30	944	1000	56

After 1000 m/z, the number of identifiable peptides grows progressively less dense

**Fig. 5:**
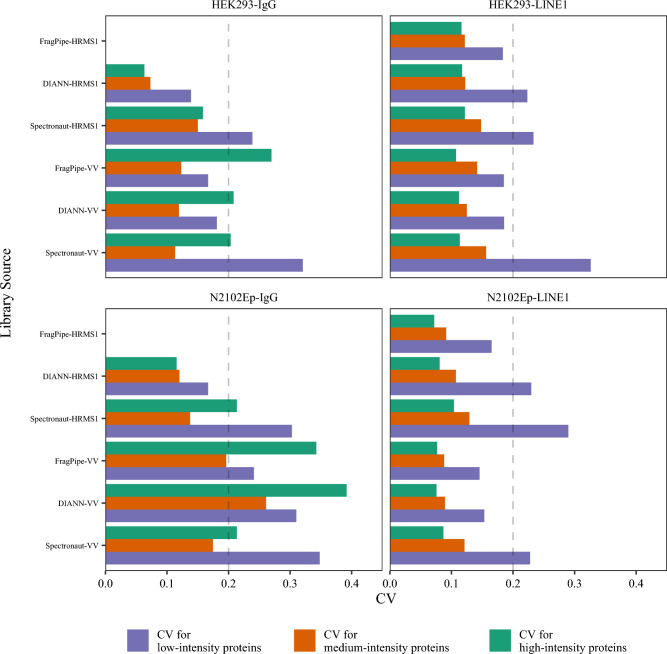
The LINE-1 data provide a direct comparison of two DIA techniques on the same samples in the same instrument: High-Resolution MS1 and “Variabele Vensters.” The proteins quantified in all samples for each cohort were separated into quintiles based on the sum of reported intensities, and these images compare the coefficients of variation for the top, middle, and bottom quintiles. The IgG samples were particularly challenging due to their low peptide diversity; surprisingly, FragPipe did not output quantities for any proteins in the IgG HRMS1 cohorts even though it could identify hundreds of peptides (552 and 669 for HEK293T and N2102Ep cell lines, respectively)

The LINE-1 data enabled the comparison of spectral library diversity and CVs directly between DDA, HRMS1, and “VV” experiments. As Fig. 5 shows, the co-IPs for LINE-1 in both HEK293T and N2102Ep cell lines provided the lowest CVs for high-intensity proteins and the highest CVs for low-intensity proteins, whether HRMS1 or VV methods were used (in this figure, spectral libraries were always derived from the data where they were applied for quantitation). Evaluating the missingness of HRMS1 and VV for the positive LINE-1 co-IPs reveals no clear “winner:” Spectronaut and FragPipe identified more proteins with no missing values in VV experiments for both cell lines, while DIA-NN identified more proteins without missing values in HRMS1 experiments for both cell lines. For the N2102Ep cell experiments, the VV experiments produced lower median CV values, while the HEK293T experiments were a mixed bag between VV and HRMS1 methods. The VV experiments were configured to use the same 60 min of instrument time as the DDA experiments, giving it a throughput advantage. VV and HRMS1 come from very different DIA design paradigms, and yet both seem to pair well with co-IP.

The availability of DDA, HRMS1 (MS1-based DIA), and VV (MS2-based DIA) for the same samples of the LINE-1 experiment make it possible to return to the question that animated this study: does switching from DDA to DIA measurement improve the information yield from co-IP experiments? FragPipe was able to perform a Match Between Runs analysis in IonQuant for the DDA experiments in its “LFQ-MBR” workflow and performing both identification and quantitation in the HRMS1 and VV experiments in its “DIA-SpecLib-Quant” workflow. The combined_protein.tsv file reported protein intensities in both “Intensity” and “MaxLFQ Intensity” columns (Yu et al. 2021). Because the MaxLFQ Intensity columns contained more missing values, we employed Intensity columns instead.

The DDA experiments quantified fewer proteins without missing values and produced higher CVs than the DIA experiments. The DDA experiments for LINE-1 pull-downs quantified 260 proteins with no missing values in HEK293T cells and quantified 293 proteins with no missing values in N2102Ep cells. The HRMS1 values were 348 and 378, respectively, while the VV experiments quantified 356 and 398 proteins unanimously. For the most intense quintile of proteins in the LINE-1 pull-downs, the CV values for all three types of instrument methods were excellent, ranging from 0.077 to 0.126. For middle intensity proteins the CVs ranged from 0.088 to 0.187. For the least intense quintile of proteins in the LINE-1 pull-downs, the CV values were consistently higher, ranging from 0.146 to 0.218. In every case, the highest CV value came from the DDA experiment for the LINE-1 pull-down in HEK293T, with the DDA experiment for N2102Ep cells being the next highest. The negative control IgG experiments in these two cell types are where the problematic performance takes place. Median CV values as high as 0.714 were produced from DDA experiments in HEK293T with the IgG pull-down. FragPipe DIA analysis failed in the negative control HRMS1 experiments for both cell lines, producing tables containing zero proteins. The low signal-to-noise environment of negative control IPs is a challenging one for both DDA and DIA quantitation, and research teams should gain familiarity with at least two different software workflows to have a fallback when one algorithm fails on a set.

## Conclusion

When used to measure proteins in affinity experiments, DIA data produced a more complete matrix of values with less variability than was observed with DDA “Match Between Runs” analysis. Therefore, researchers who aim to quantify differences rather than expecting presence-and-absence changes can certainly benefit from DIA. The first key recommendation to come from this study is that performing separate DDA experiments to produce a spectral library for co-IP is unnecessary. DIA-NN, MSFragger-DIA, and Spectronaut Pulsar can all derive spectral libraries directly from DIA experiments that are of at least equivalent sensitivity to those from DDA experiments. This capability comes with a couple footnotes, however; presently, Bruker diaPASEF MS/MS scans can only be identified by DIA-NN and Spectronaut; and FragPipe requires mzML conversion for identification from SCIEX WIFF format. The choice of available software for interpreting DIA experiments is broadening with time. The FragPipe combination of MSFragger-DIA for identification and DIA-NN for quantitation can return protein quantity tables in minutes for computers with multiple CPU cores and 16 GB + of RAM. DIA-NN spectral libraries often proved to be the largest from a given DIA experiment, though analyzing large experiments quickly in this software requires a server-class CPU. DIA-NN will be of particular interest to researchers who want to identify Bruker diaPASEF directly. Spectronaut has a long tenure in the space of DIA analysis, and its identification rate is quite similar to that of DIA-NN. Moreover, Spectronaut’s warning messages are a useful guide to knowing when a sample’s low peptide diversity is compromising quantitative accuracy, and its DIA Acquisition Method Overview was very useful to check for errors in our DIA method development. Spectronaut’s support of DIA quality control is a compelling feature. MaxDIA, the DIA quantification engine in MaxQuant, identified consistently fewer peptides and produced CV values that were substantially higher than for other algorithms, and its run-time was compromised by continually reading and writing thousands of temporary files. Hopefully, continuing research into this popular software will drive improvements in its performance to make it comparable to the other three workflows we explored.

Early comparisons of database search engines for low-resolution MS/MS DDA data frequently showed only modest overlaps in identified peptides between search engines (Kapp et al. 2005). As proteomics shifted to high-resolution MS/MS, the search engines identified far more consistent peptide lists (Tu et al. 2015). The peptide overlaps in spectral libraries for this project suggest that DIA identification has already reached a state of great consistency among search engines that can identify DIA MS/MS directly. The largest differences seen between these spectral libraries came from comparing the sets of peptides from DDA experiments to those from DIA experiments. During development, this project examined dozens of DIA data sets in ProteomeXchange, and they revealed little consistency among DIA methods. PXD030383, for example, used a DIA method of 150 windows, each 4 m/z wide, ranging from 400 to 1000 m/z (Barbier-Torres et al. 2022). Meanwhile, the SPION set (PXD023278) employed 24 fixed windows, each 36 m/z wide, covering the region between 350 and 1200 m/z. The HRMS1 method employed in the IP Inputs and LINE-1 experiments follows the DIA paradigm by its regularly scheduled sampling of windows of precursors (each 9 m/z wide, ranging from 400 to 1200 m/z), but it departs the DIA mainstream by anticipating that quantitation will build chromatograms from MS scans rather than MS/MS scans. The use of variable window designs and the incorporation of ion mobility times adds additional dimensions for method diversity. Whatever design a lab selects, it is apparent that DIA is here to stay, and biologists carrying out affinity proteomics experiments can benefit from it just as much as any other proteomics community. The low peptide diversity and small sample volumes, especially of negative controls, will pose a challenge to existing software frameworks, but it appears that when one software workflow fails on a data set, another likely exists to complement its results. Increased sensitivity of identification paired with lower CVs of quantitation is a winning combination for taking co-IP analysis to the next level.

## Supplementary Information

Below is the link to the electronic supplementary material.

Supplementary file1 (DOCX 5766 KB)

Supplementary file2 (ZIP 700861 KB)

## Data Availability

All DDA and DIA raw data are available from ProteomeXchange (see below for PXD accessions). See also MSV000095426 at massive.ucsd.edu for a repackaging of the Bruker DUBs set as well as TIMSCONVERT mzMLs. Spectral libraries, protein quantitation tables, R scripts for analysis, tables of quality metrics, and Microsoft Excel versions of Tables 1 and 2 are available in Supplementary File 1. PXD054265: M. musculus gut extract PXD023278: M. musculus tissue extract and lysosomal enrichment PXD054173: H. sapiens co-IP of LINE-1 ORF1p PXD002965: D. melanogaster co-IP of RACK1 PXD017517: M. musculus co-IP of ID4 PXD031848: H. sapiens activity-based profiling of deubiquitylating enzymes.
