# Supplementary material for: Interrogating data-independent acquisition LC–MS/MS for affinity proteomics
Source: J Proteins Proteom. 2024 Sep 17;15(3):281–98. doi: 10.1007/s42485-024-00166-4 (PMC11452513; doi:10.1007/s42485-024-00166-4)
Supplement: Supplementary file 1 — Supplementary file1 (DOCX 5766 KB) [file 42485_2024_166_MOESM1_ESM.docx]

Supplementary Information for

Interrogating Data-Independent Acquisition LC-MS/MS for affinity proteomics

Contents

[Table 1: Cohort definitions for each data set 2](#_heading=h.gjdgxs)

[Table 1A: Thermo IP Inputs 2](#_heading=h.30j0zll)

[Table 1B: Thermo SPION 3](#_heading=h.1fob9te)

[Table 1C: Thermo LINE-1 4](#_heading=h.3znysh7)

[Table 1D: SCIEX RACK1 4](#_heading=h.2et92p0)

[Table 1E: SCIEX ID4 4](#_heading=h.tyjcwt)

[Table 1F: Bruker DUBs 5](#_heading=h.3dy6vkm)

[Table 2: Spectral library diversity metrics 6](#_heading=h.1t3h5sf)

[Table 2A: Thermo IP Inputs 6](#_heading=h.4d34og8)

[Table 2B: Thermo SPION 7](#_heading=h.2s8eyo1)

[Table 2C: Thermo LINE-1 8](#_heading=h.17dp8vu)

[Table 2D: SCIEX RACK1 9](#_heading=h.3rdcrjn)

[Table 2E: SCIEX ID4 9](#_heading=h.26in1rg)

[Table 2F: Bruker DUBs 10](#_heading=h.lnxbz9)

[Table 3: Protein quantity table protein diversity and CV values 10](#_heading=h.35nkun2)

[Table 3A: Thermo IP Inputs 11](#_heading=h.1ksv4uv)

[Table 3B: Thermo SPION 11](#_heading=h.44sinio)

[Table 3C: Thermo LINE-1 12](#_heading=h.2jxsxqh)

[Table 3D: SCIEX RACK1 13](#_heading=h.z337ya)

[Table 3E: SCIEX ID4 13](#_heading=h.3j2qqm3)

[Table 3F: Bruker DUBs 14](#_heading=h.1y810tw)

[Figure 1: UpSet plots comparing spectral library peptide content 14](#_heading=h.4i7ojhp)

[Figure 1A: Thermo IP Inputs 15](#_heading=h.2xcytpi)

[Figure 1B: Thermo SPION 16](#_heading=h.1ci93xb)

[Figure 1C: Thermo LINE-1 18](#_heading=h.3whwml4)

[Figure 1D: SCIEX RACK1 20](#_heading=h.2bn6wsx)

[Figure 1E: SCIEX ID4 21](#_heading=h.qsh70q)

[Figure 1F: Bruker DUBs 23](#_heading=h.3as4poj)

[Figure 2: Protein diversity for spectral libraries, any quantitation, and unanimous quantitation 26](#_heading=h.1pxezwc)

[Figure 3: Coefficients of Variation for intense, medium intensity, and low-intensity proteins 32](#_heading=h.49x2ik5)

[Text 1: R script for analyzing spectral libraries and protein quantity tables 38](#_heading=h.2p2csry)

[Text 2: Low peptide diversity in mock controls 40](#_heading=h.147n2zr)

[File 1: Zip of compressed spectral libraries and protein quantity tables 43](#_heading=h.3o7alnk)

# Table 1: Cohort definitions for each data set

Each of the six data sets can be subdivided to cohorts that were more uniformly collected and which should have more similar peptide content. These tables report the sets of raw data that correspond to each cohort label within a data set.

## Table 1A: Thermo IP Inputs


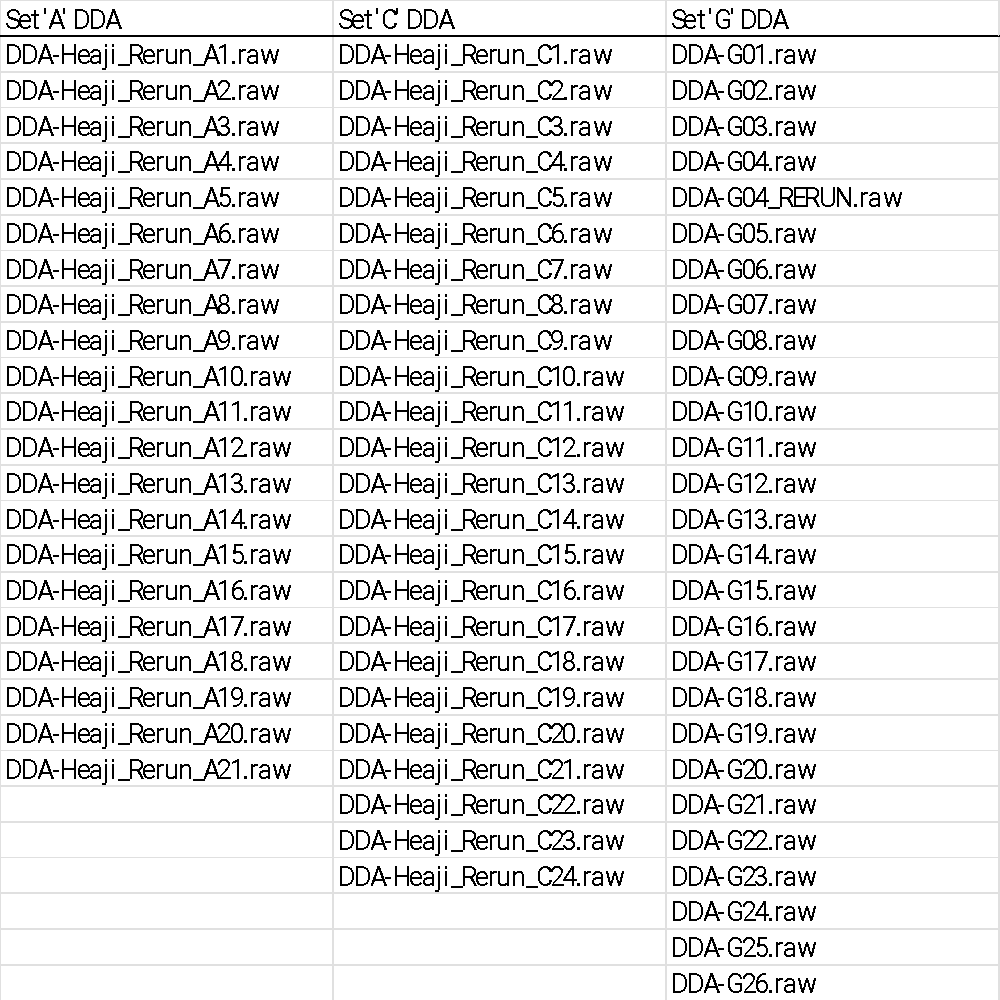


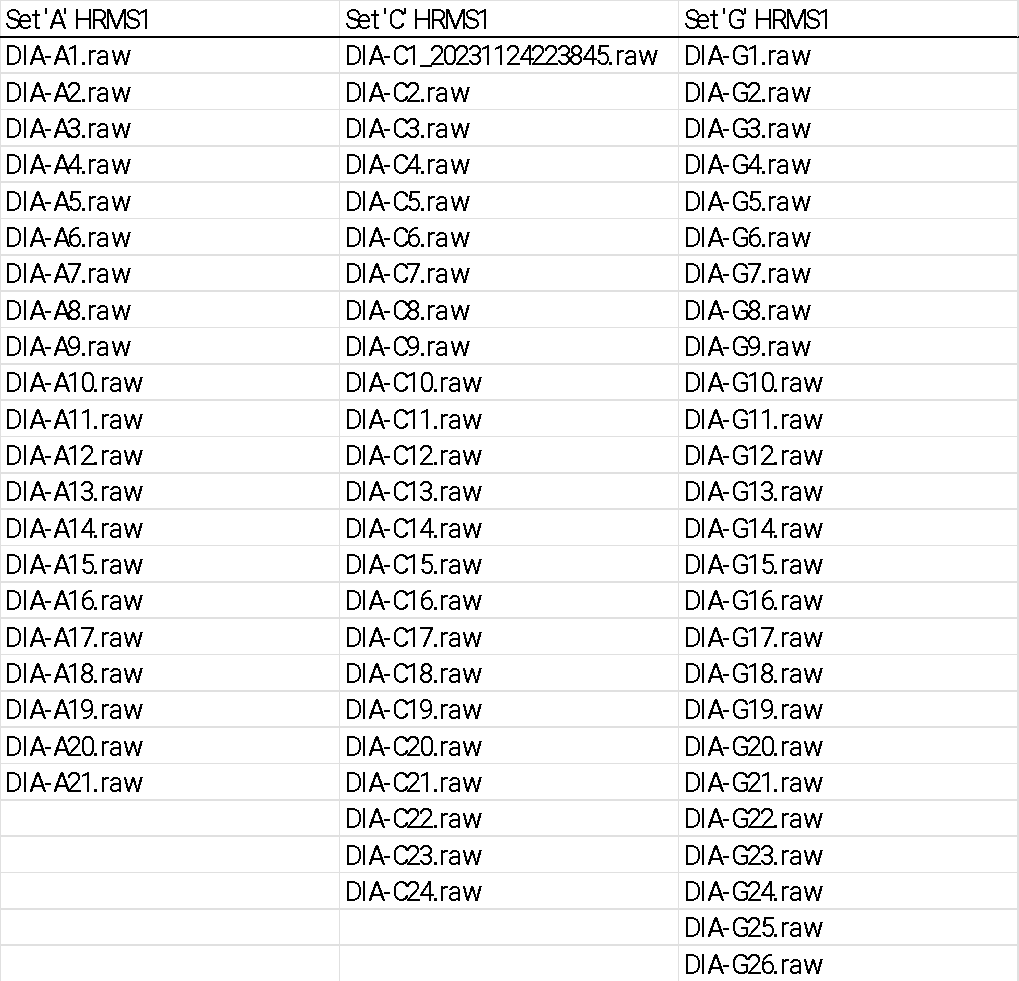


## Table 1B: Thermo SPION


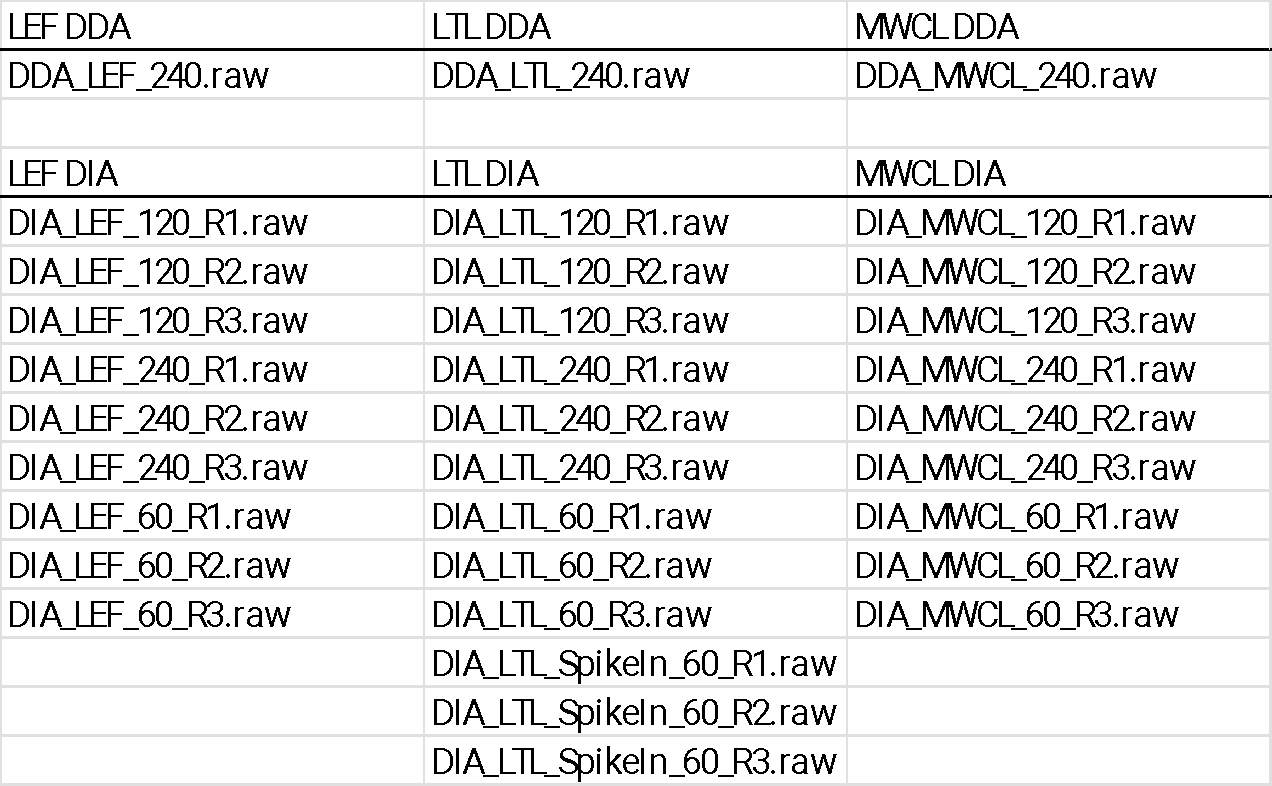


## Table 1C: Thermo LINE-1


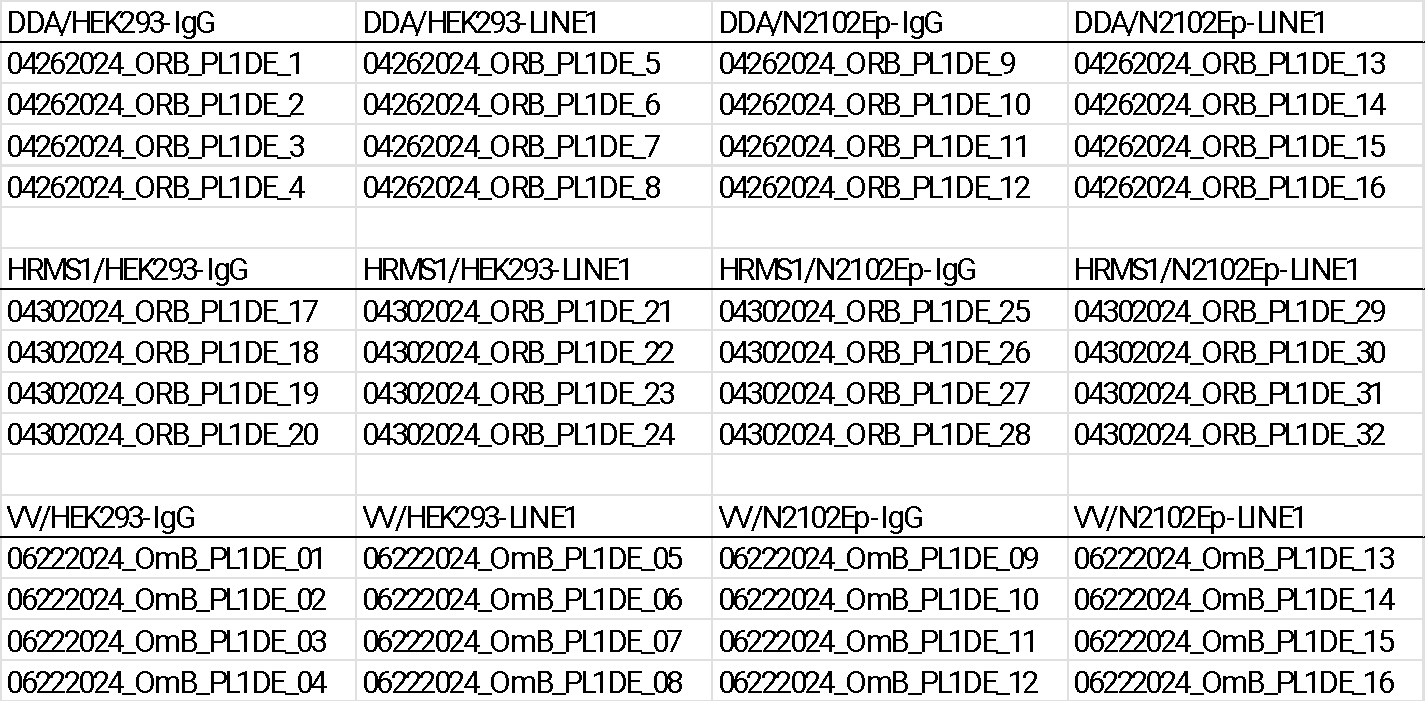


## Table 1D: SCIEX RACK1


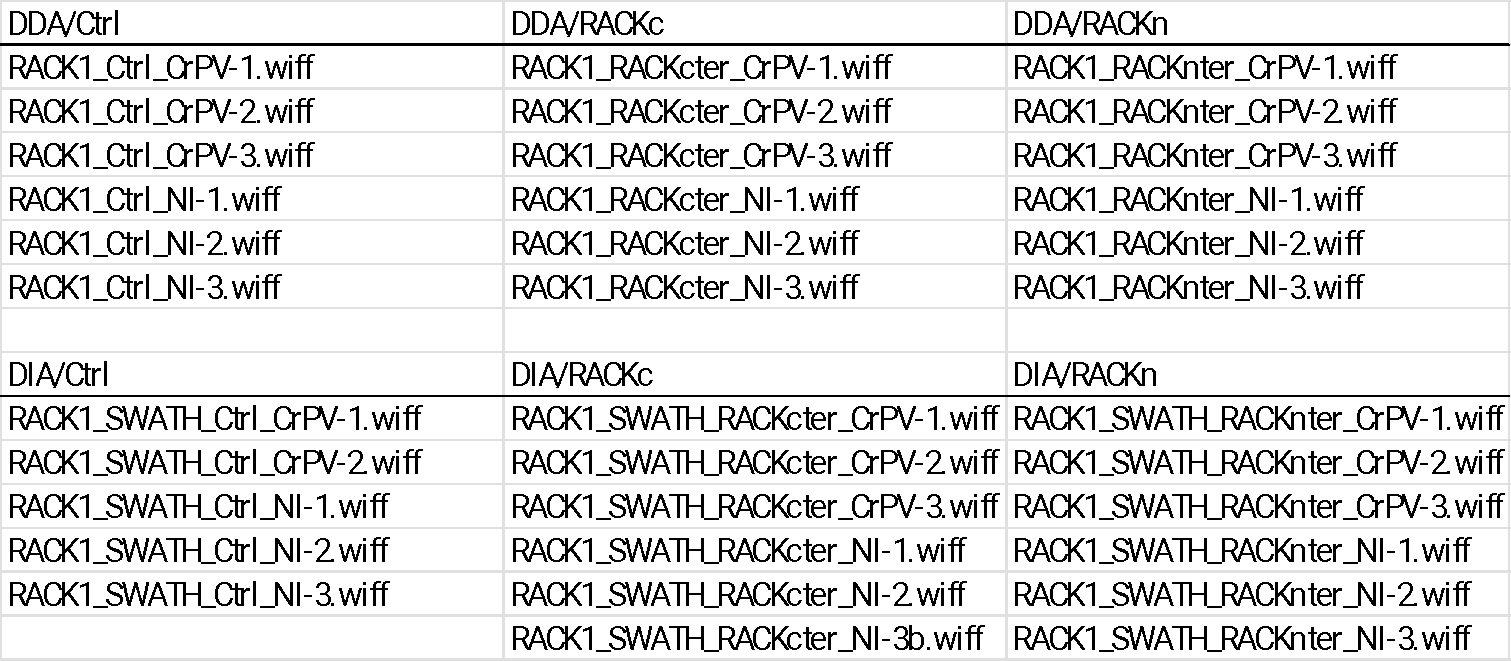


## Table 1E: SCIEX ID4


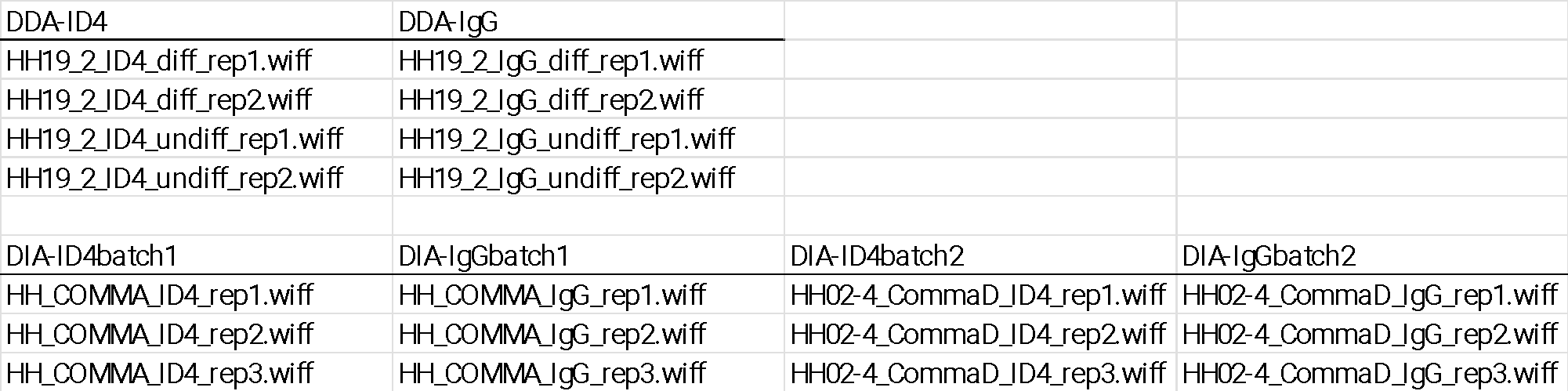


## Table 1F: Bruker DUBs


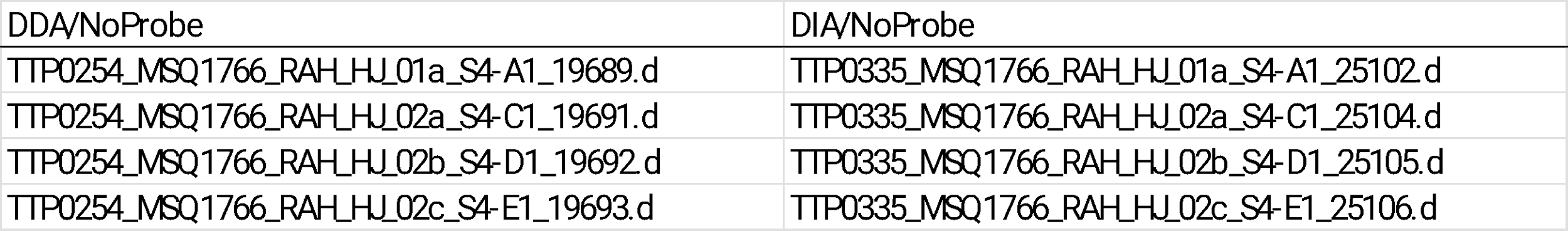


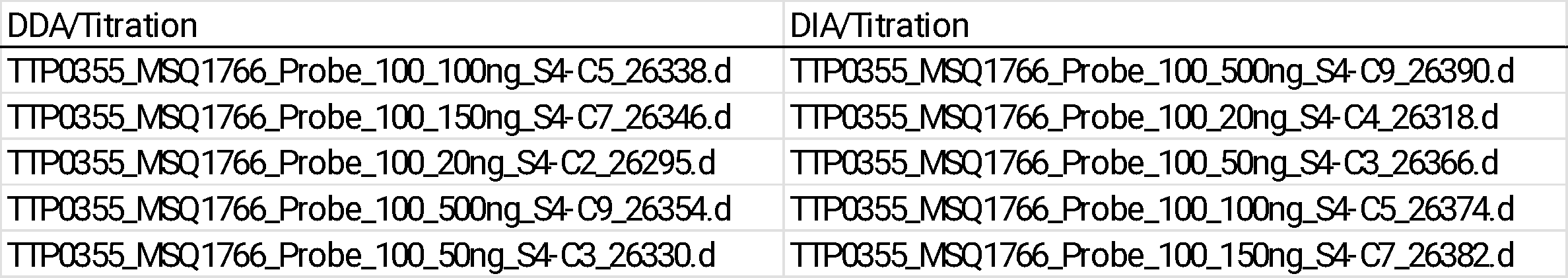


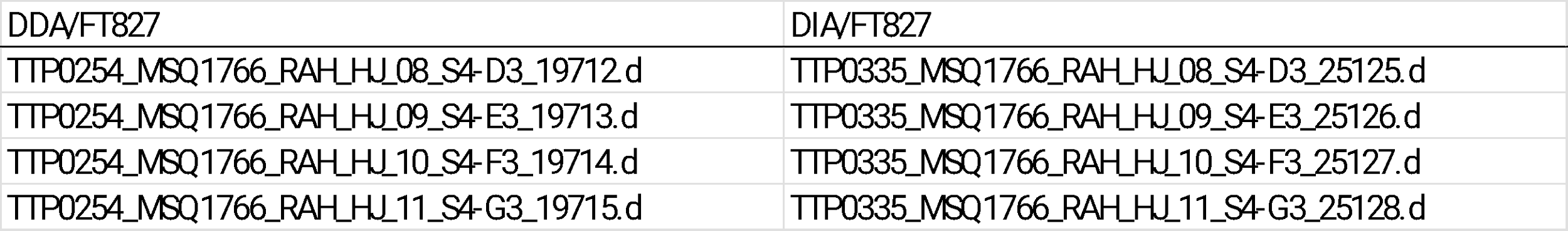


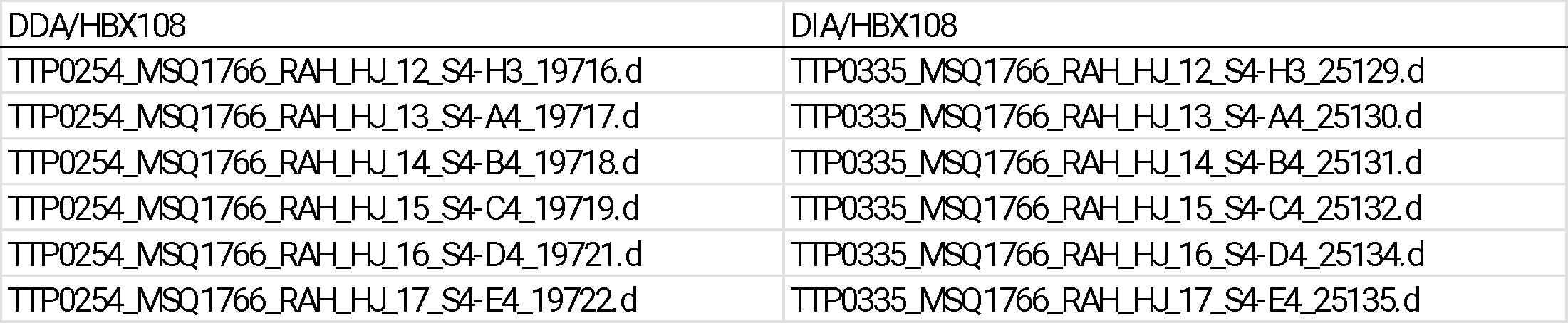


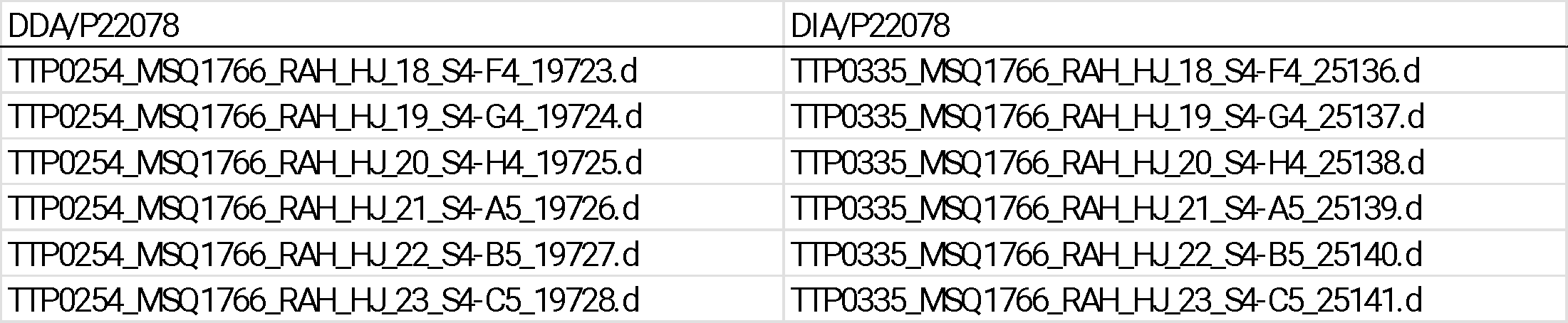


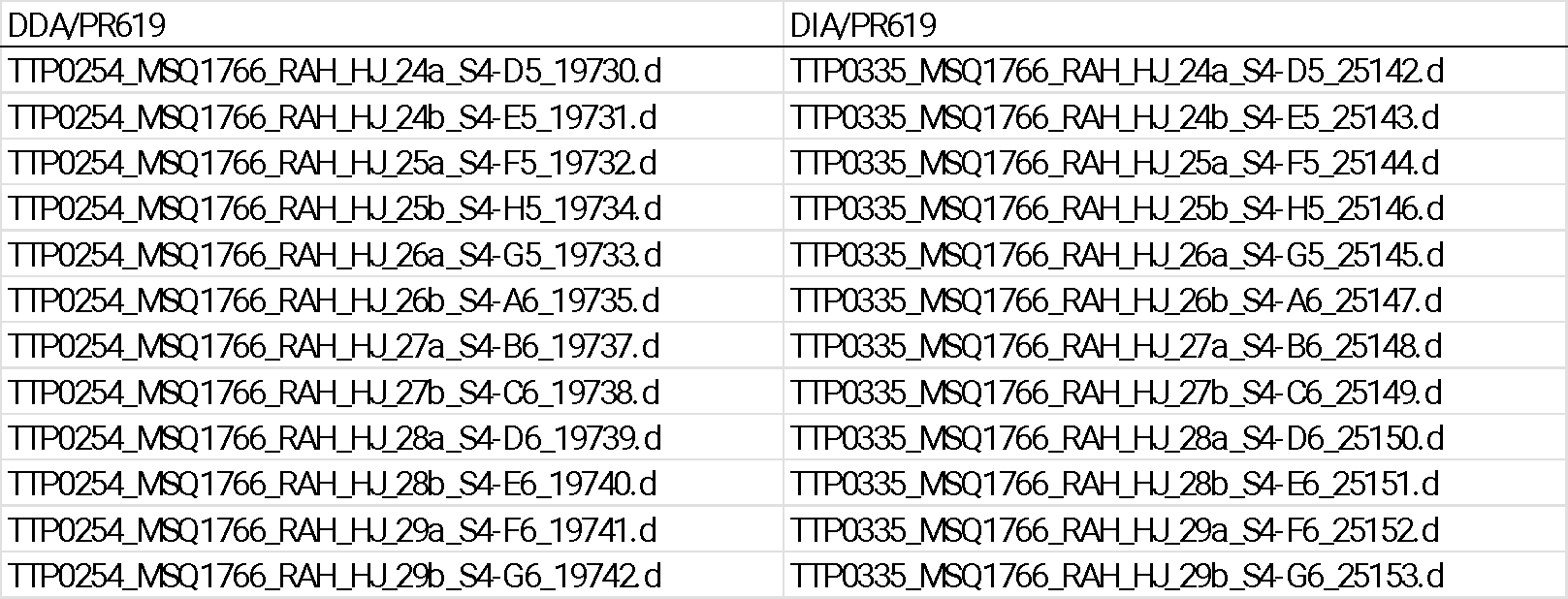


# Table 2: Spectral library diversity metrics

For each cohort in each data set, we reported the diversity of each spectral library in terms of the number of different gene names reported, the number of distinct protein groups reported, the number of distinct peptides reported, the number of distinct peptides in a given modification state reported, and the number of charge state variants for a given modification state of a given peptide (MPZs) reported.

## Table 2A: Thermo IP Inputs


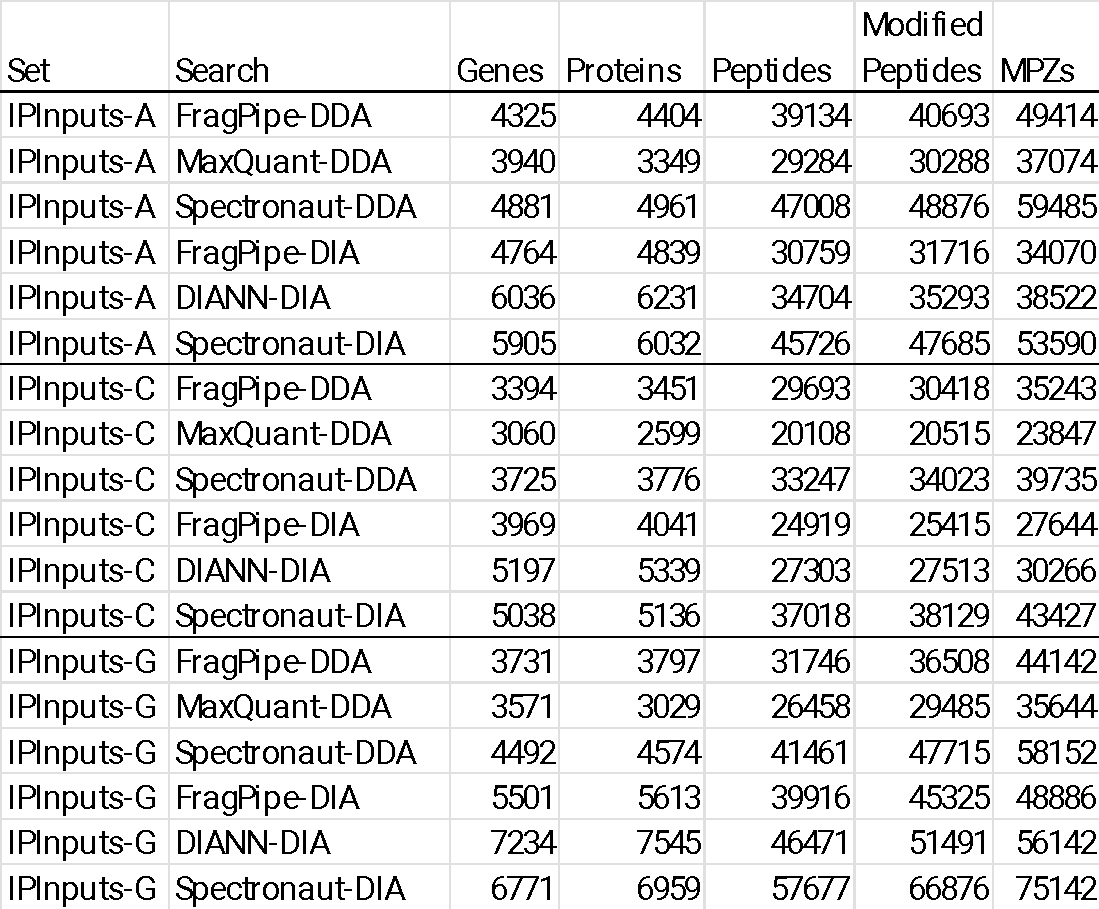


## Table 2B: Thermo SPION


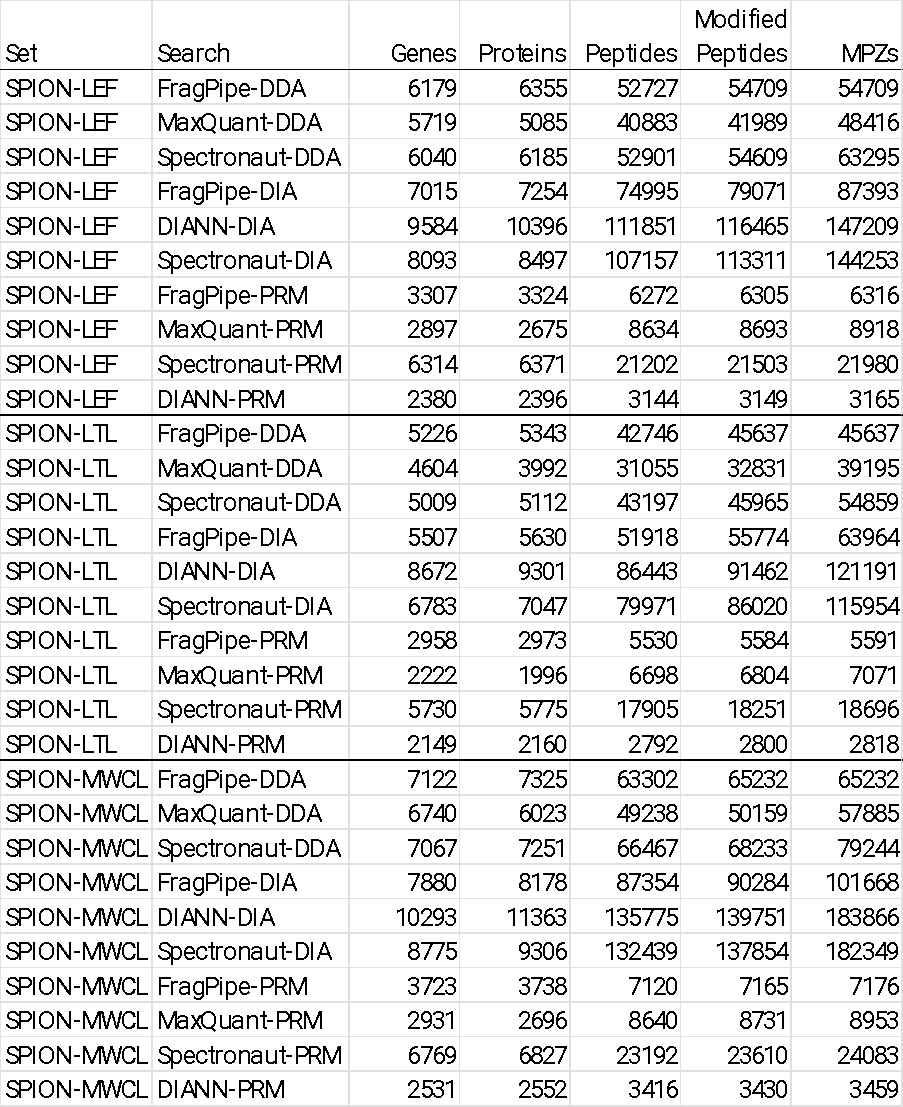


## Table 2C: Thermo LINE-1


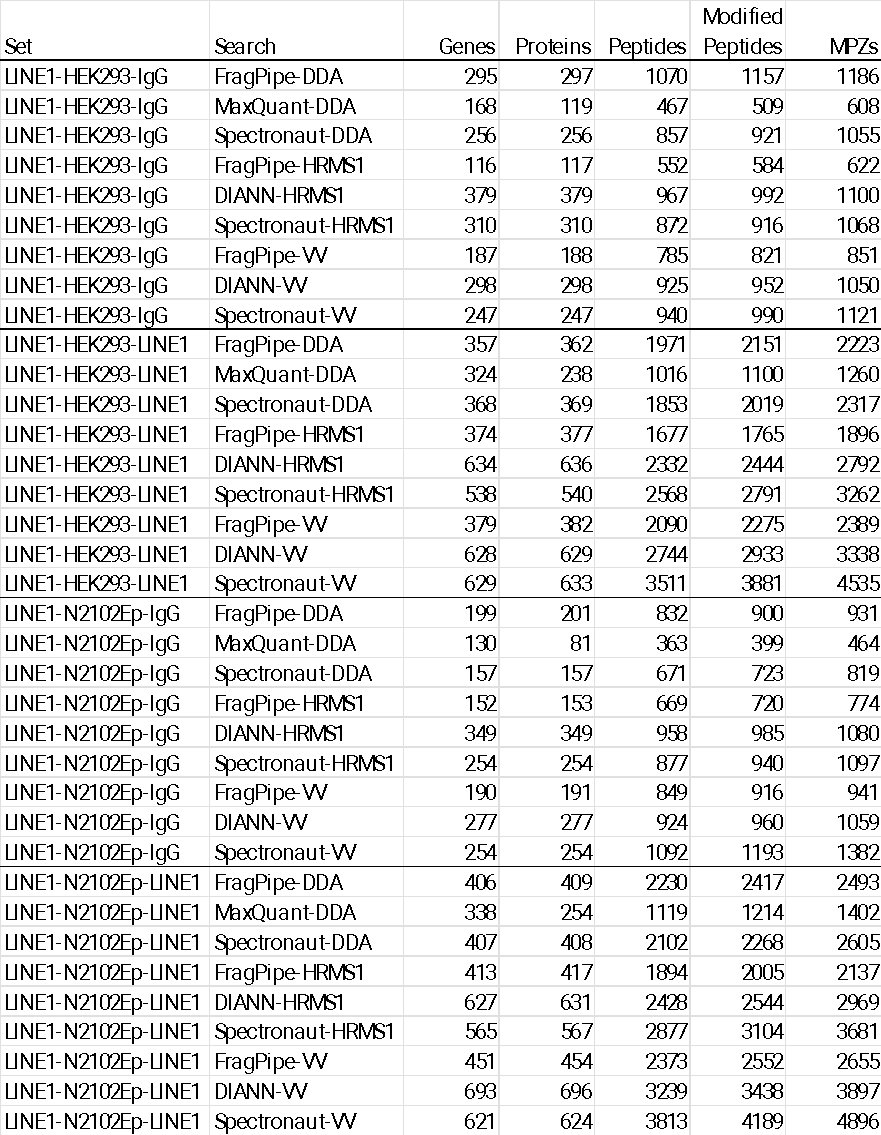


## Table 2D: SCIEX RACK1


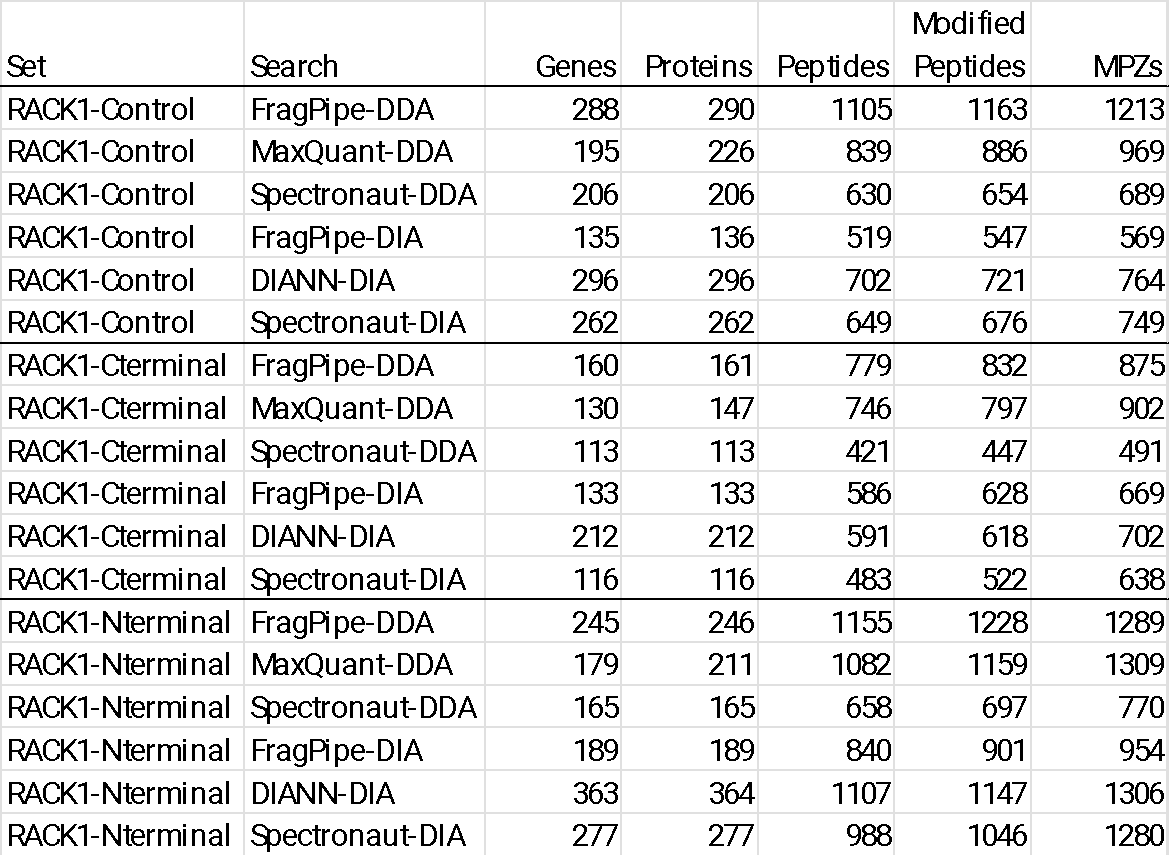


## Table 2E: SCIEX ID4


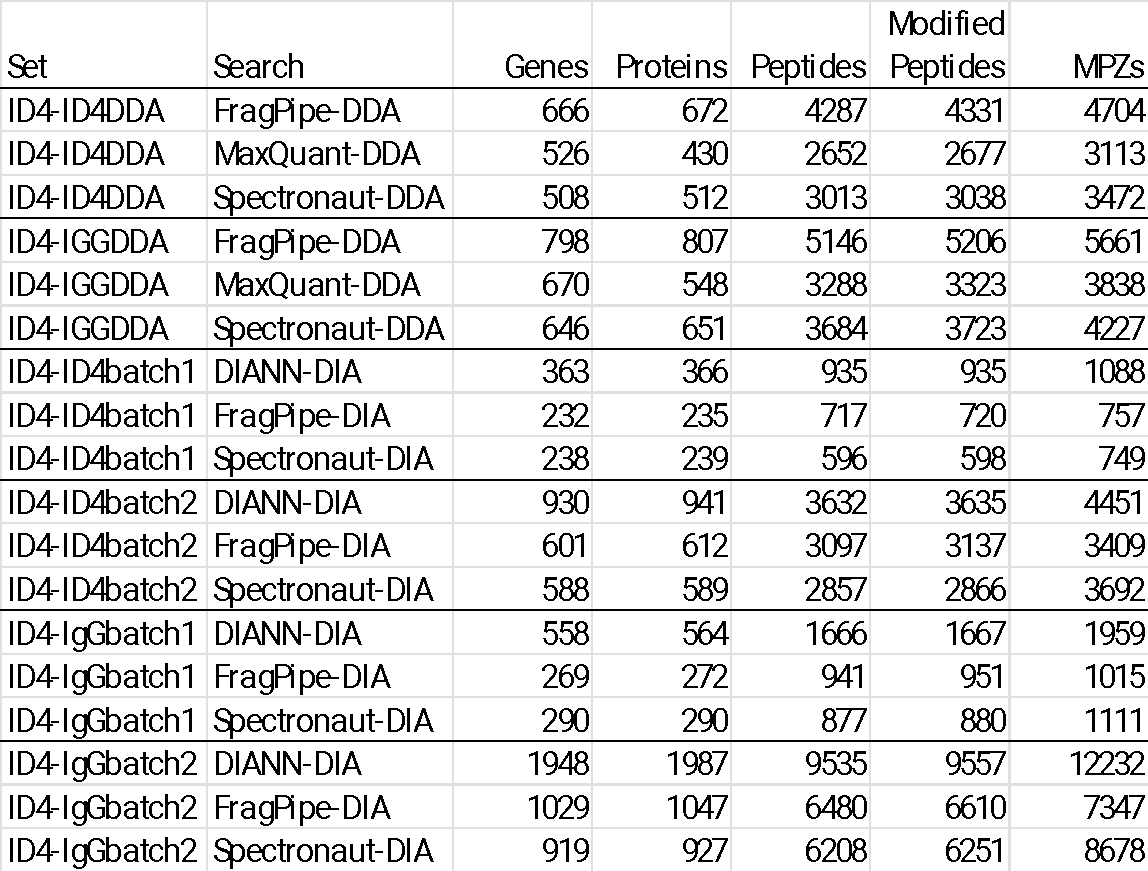


## Table 2F: Bruker DUBs


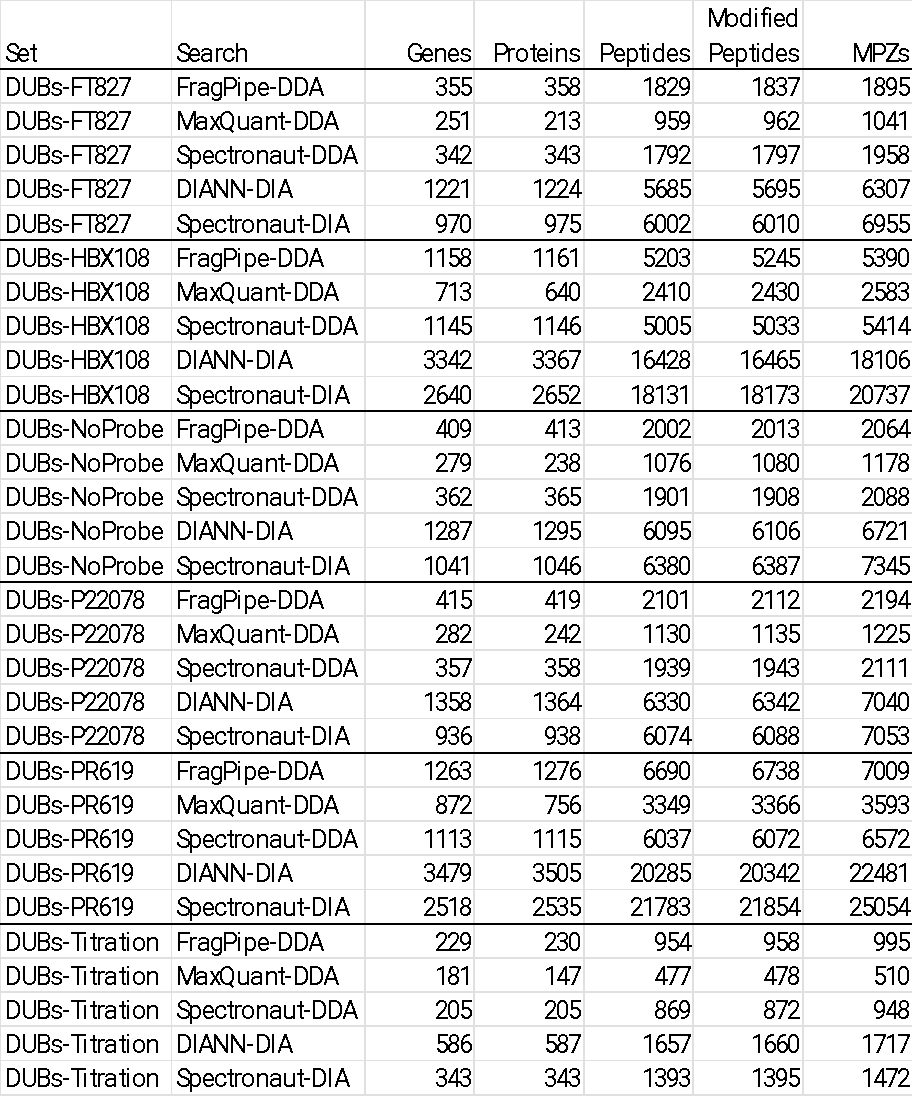


# Table 3: Protein quantity table protein diversity and CV values

To evaluate protein quantity tables, we first reported the number of proteins in which any quantity was reported and the number of proteins for which all quantities were reported (“unanimous proteins”). We separated unanimous proteins to quintiles based on the sum of intensity values. Then we computed the CV (standard deviation divided by mean intensity) for each protein and reported the median CV within each quintile.

## Table 3A: Thermo IP Inputs


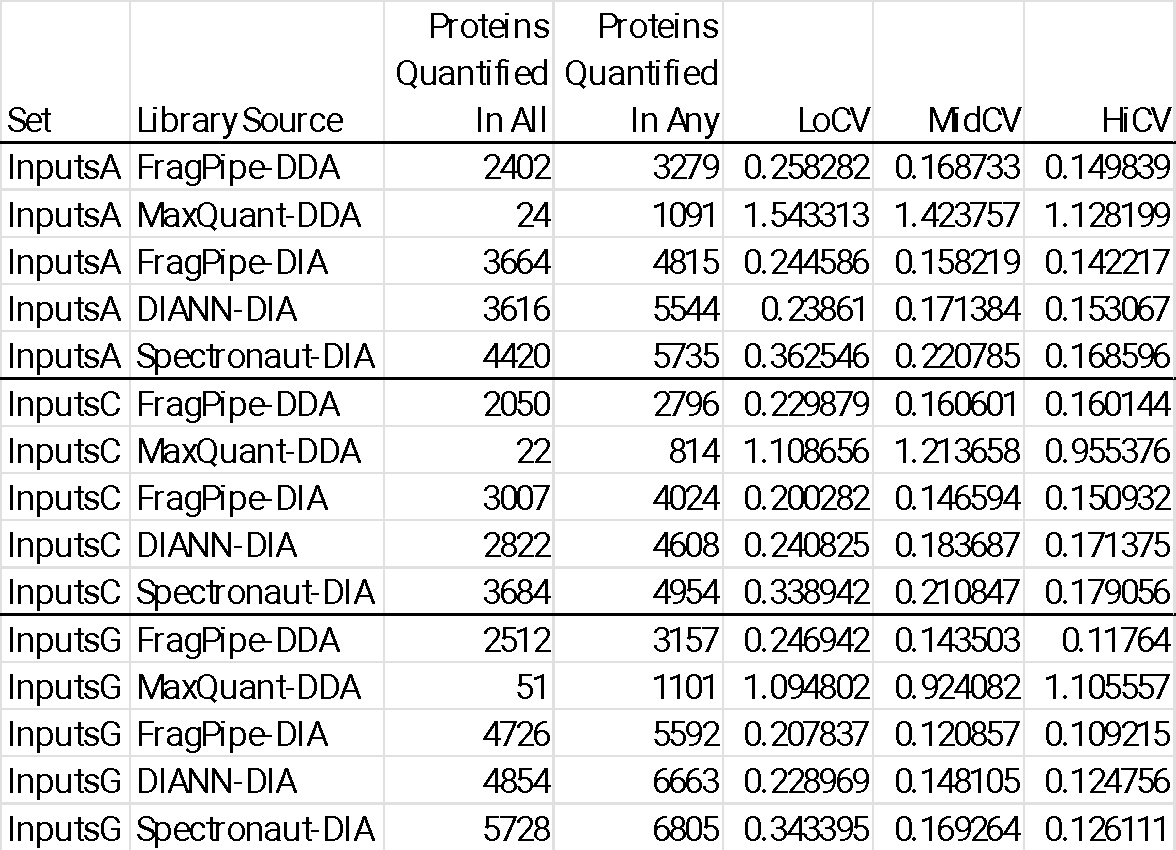


## Table 3B: Thermo SPION


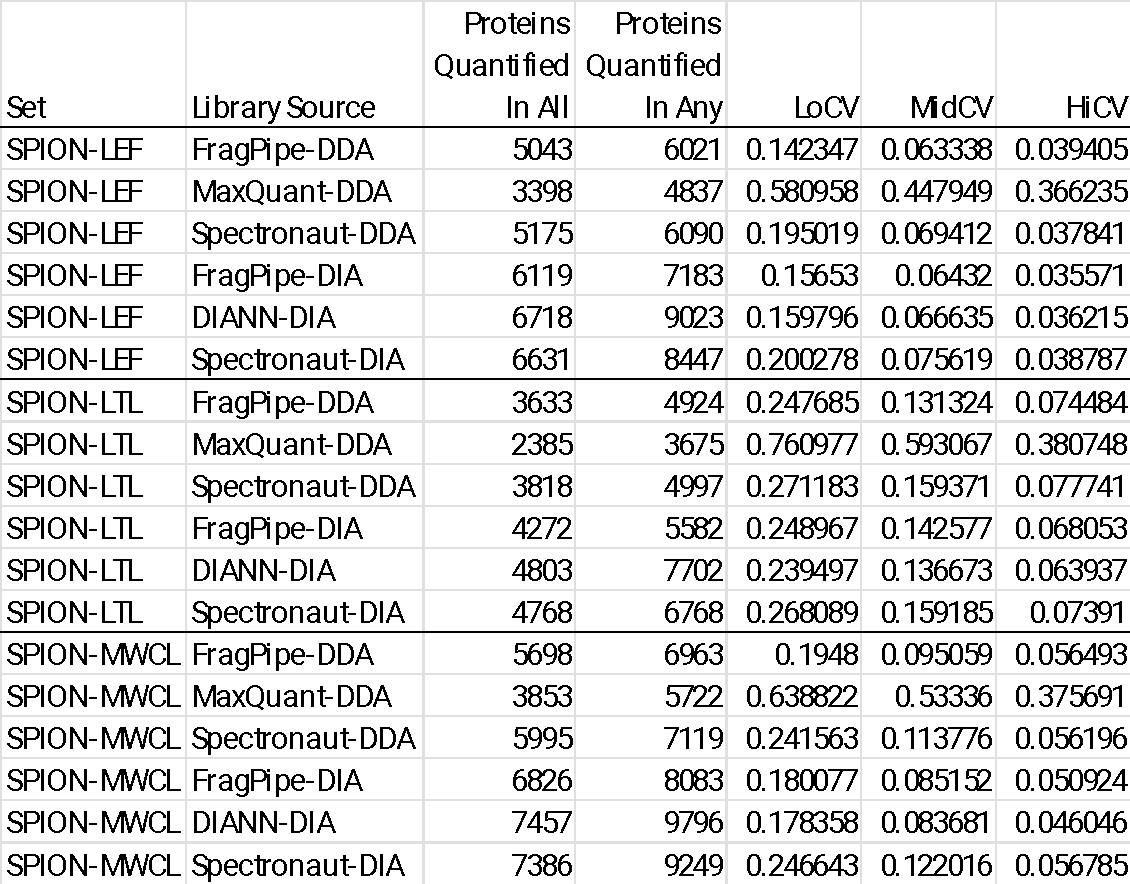


## Table 3C: Thermo LINE-1


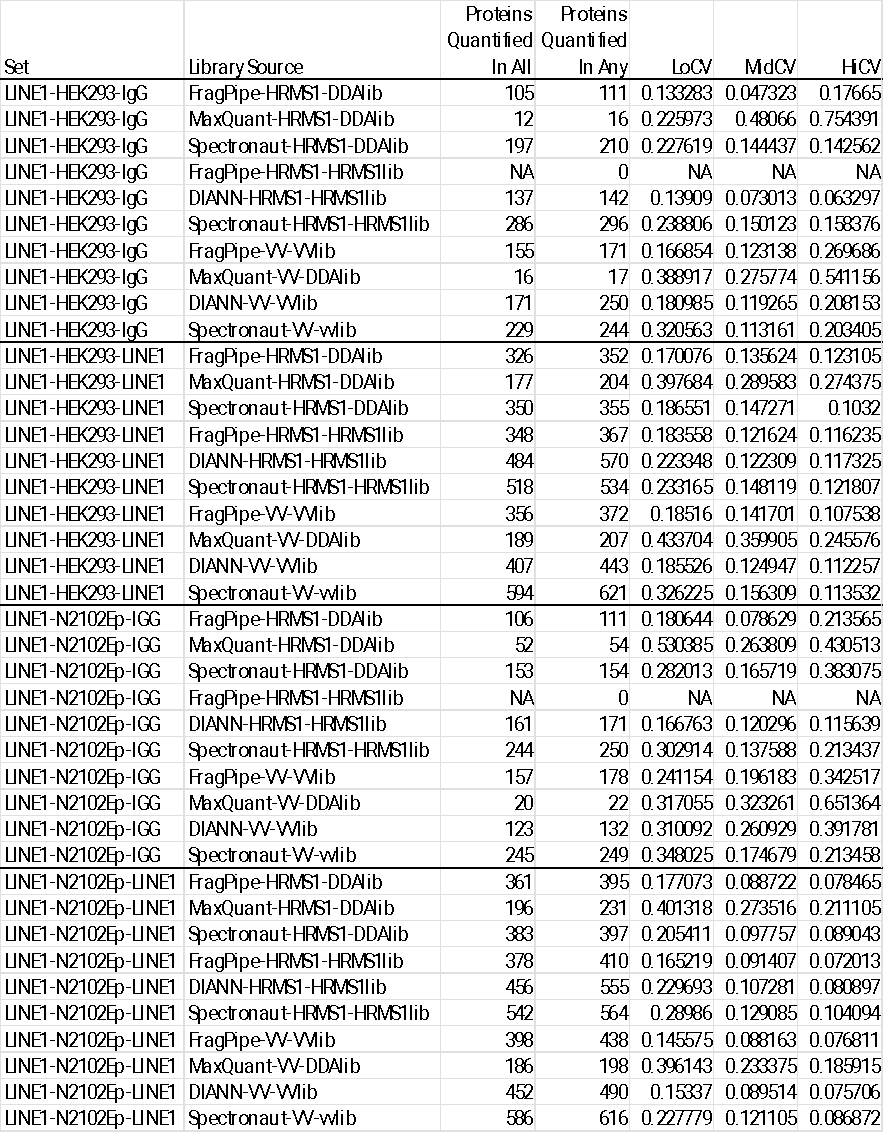


## Table 3D: SCIEX RACK1


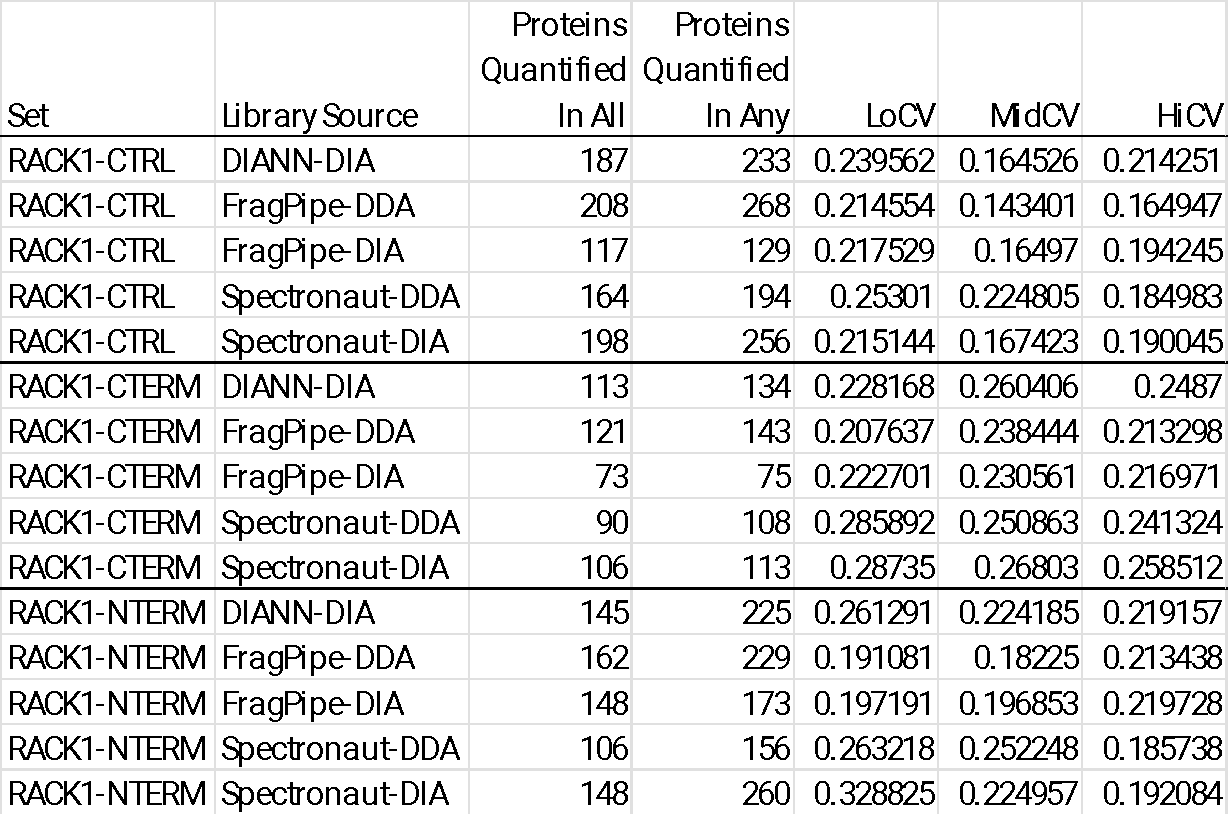


## Table 3E: SCIEX ID4


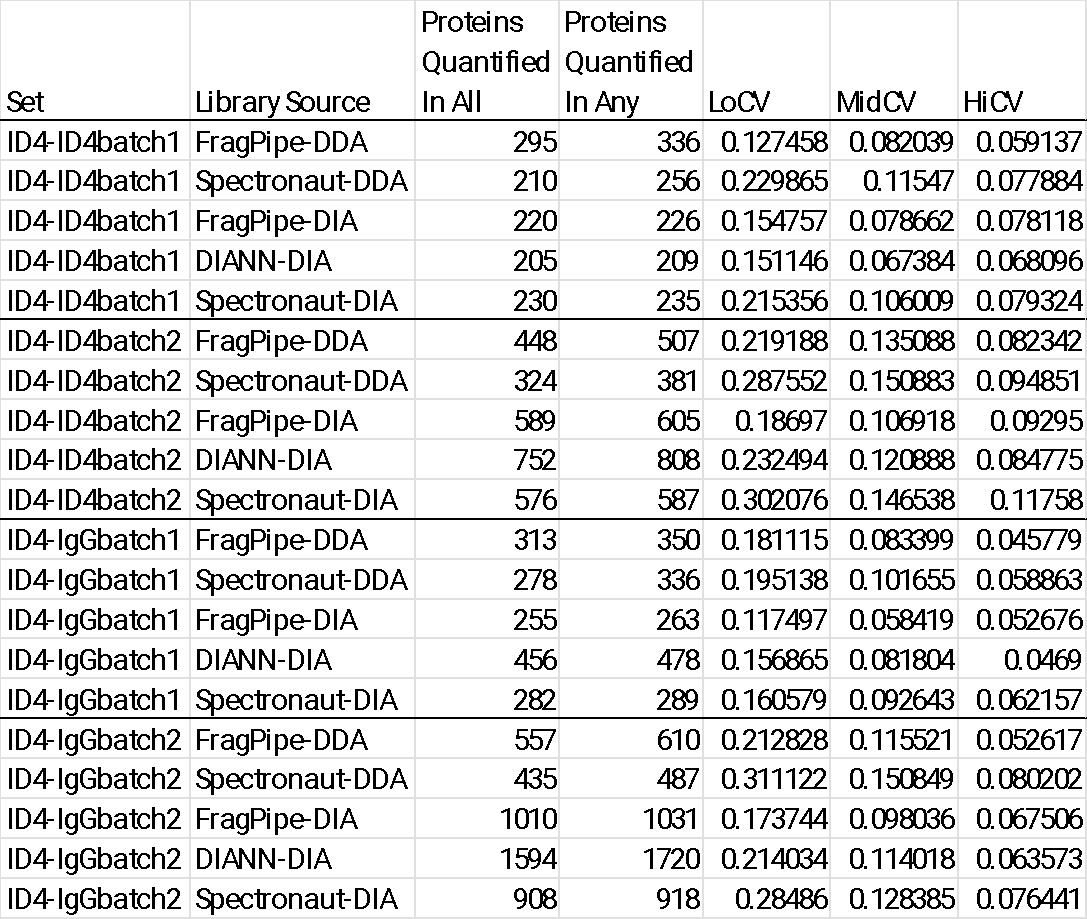


## Table 3F: Bruker DUBs


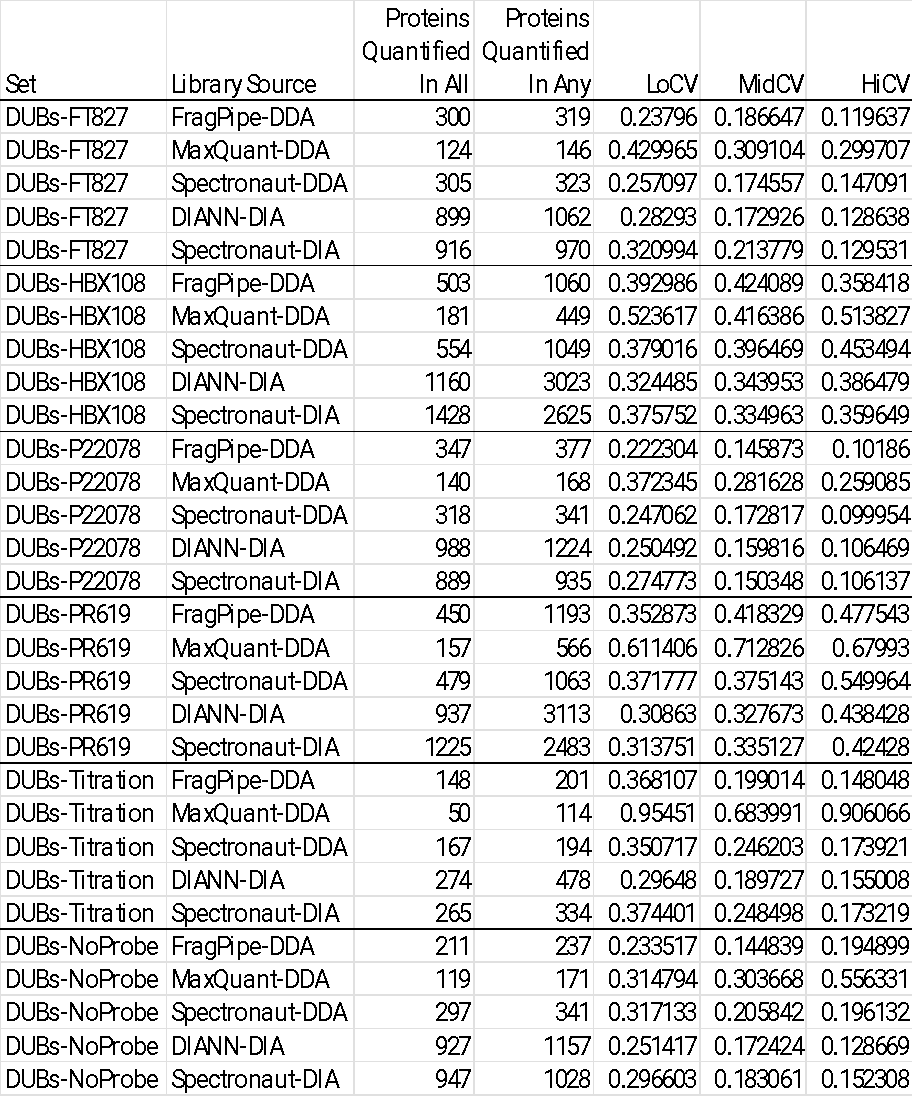


# Figure 1: UpSet plots comparing spectral library peptide content

UpSet plots are an updated way to present overlap information compared to their Venn Diagram cousins. They are useful for visualizing the sizes of sets (by the bar graphs at the lower left) and the major intersections of those sets (the sizes of which are given by bars in descending order). We used them to visualize the overlaps among peptide spectral libraries created by searching either DDA or DIA data representing related samples.

## Figure 1A: Thermo IP Inputs


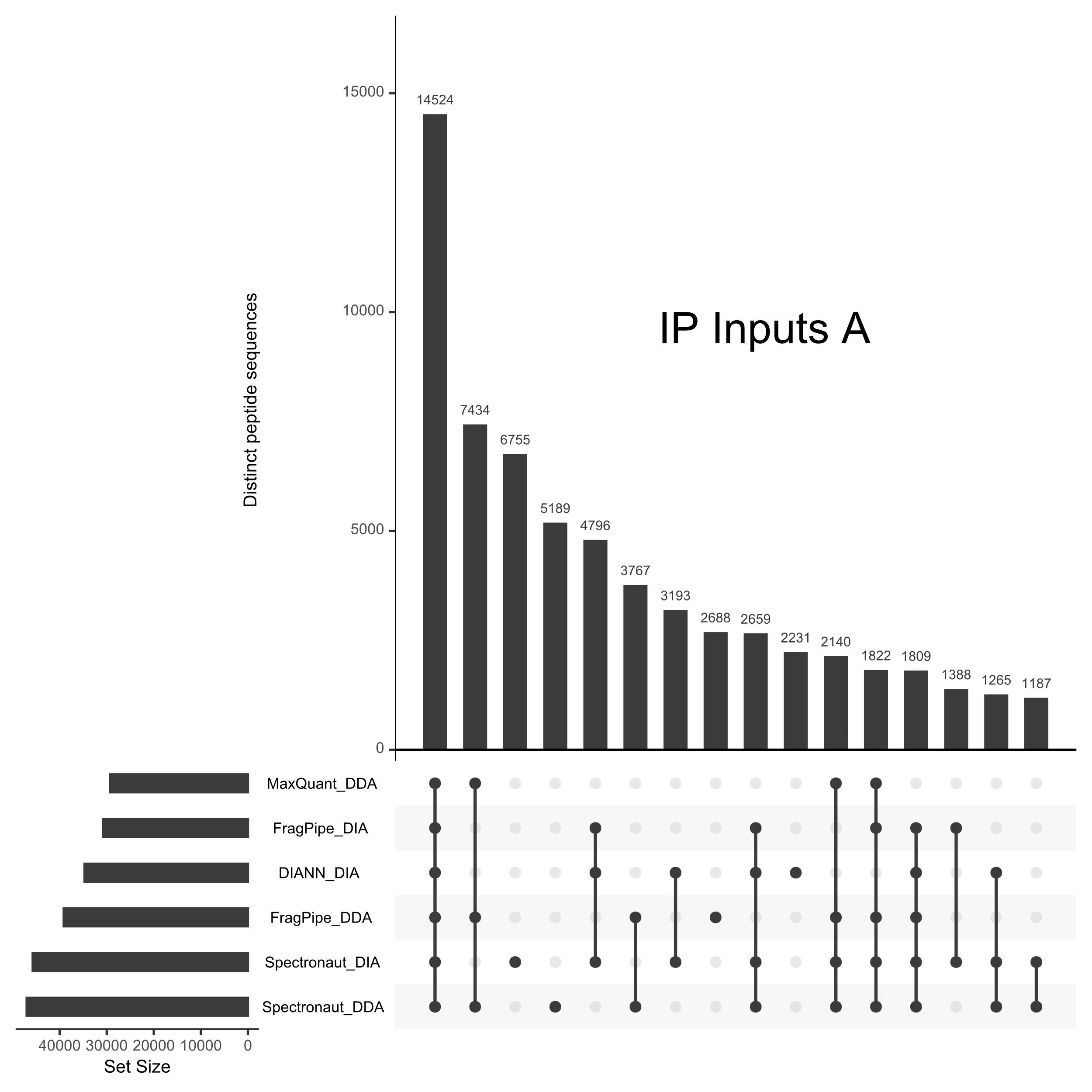


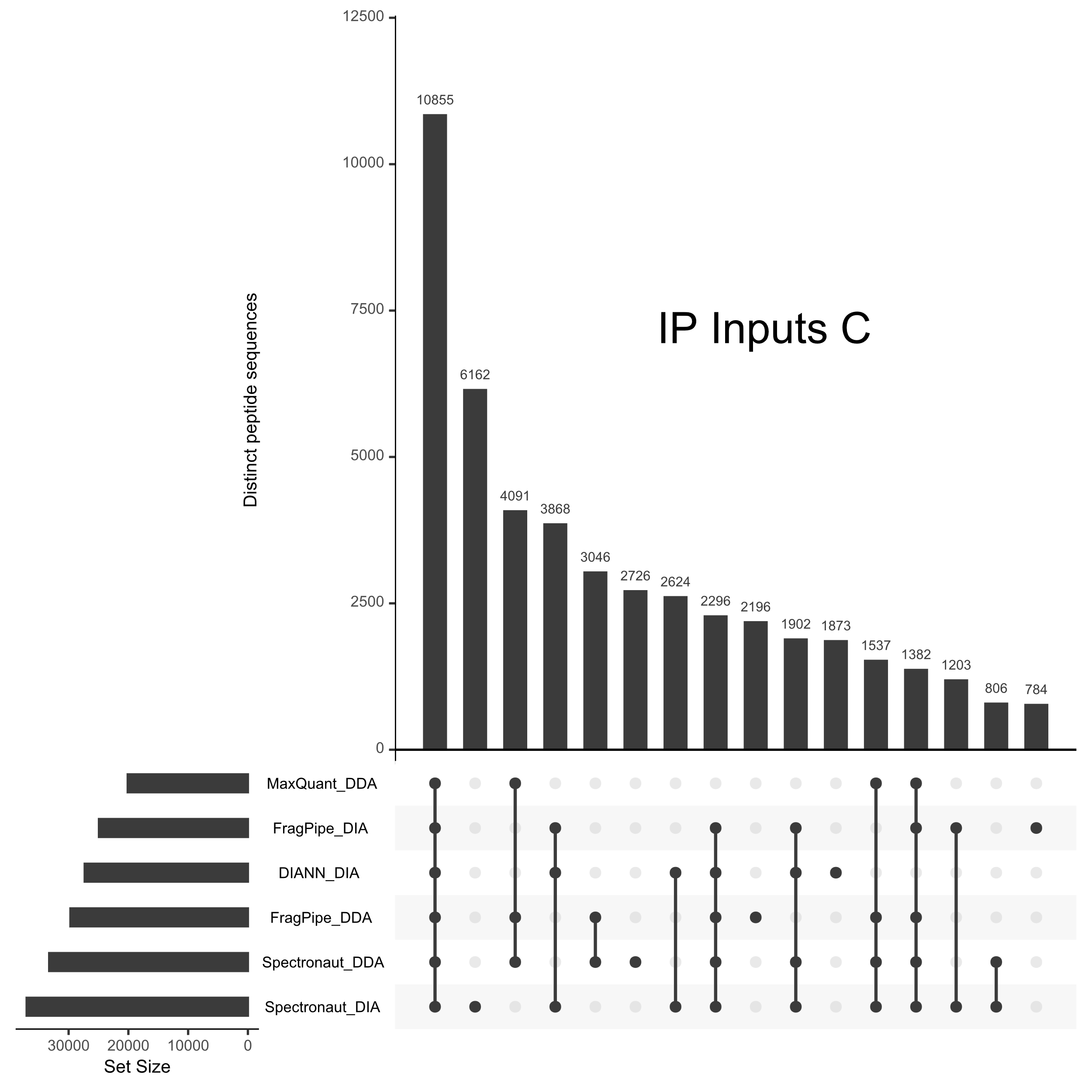


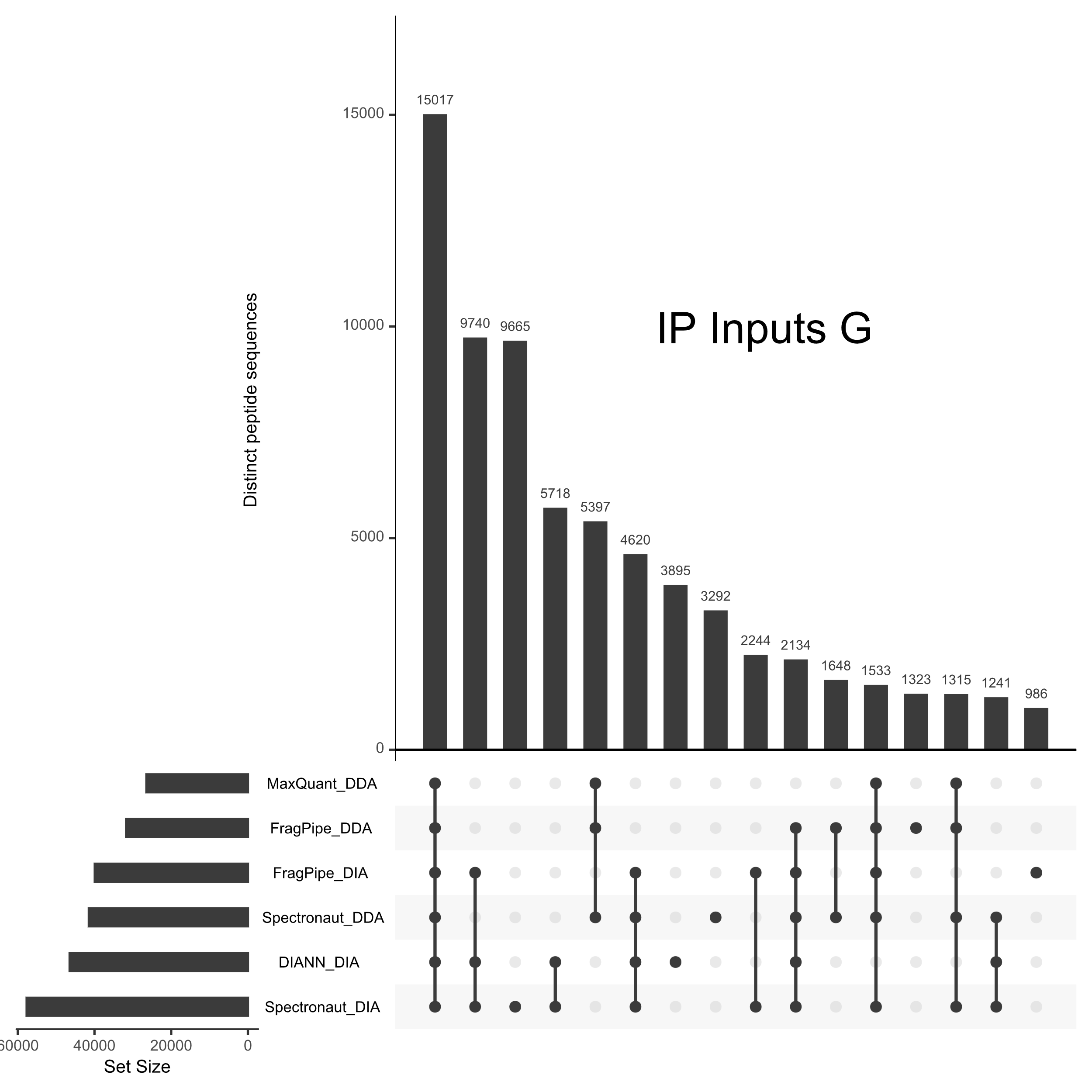


## Figure 1B: Thermo SPION


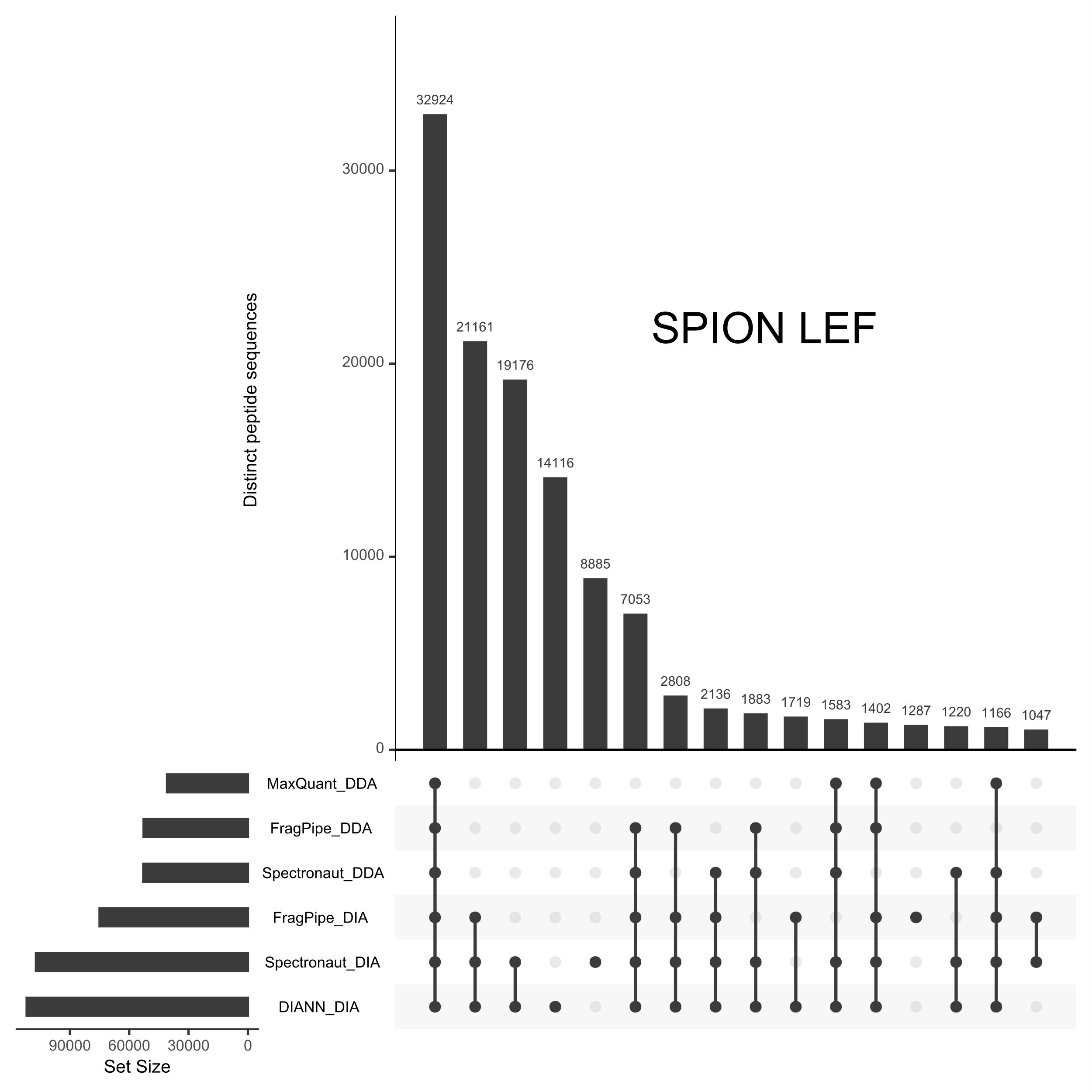


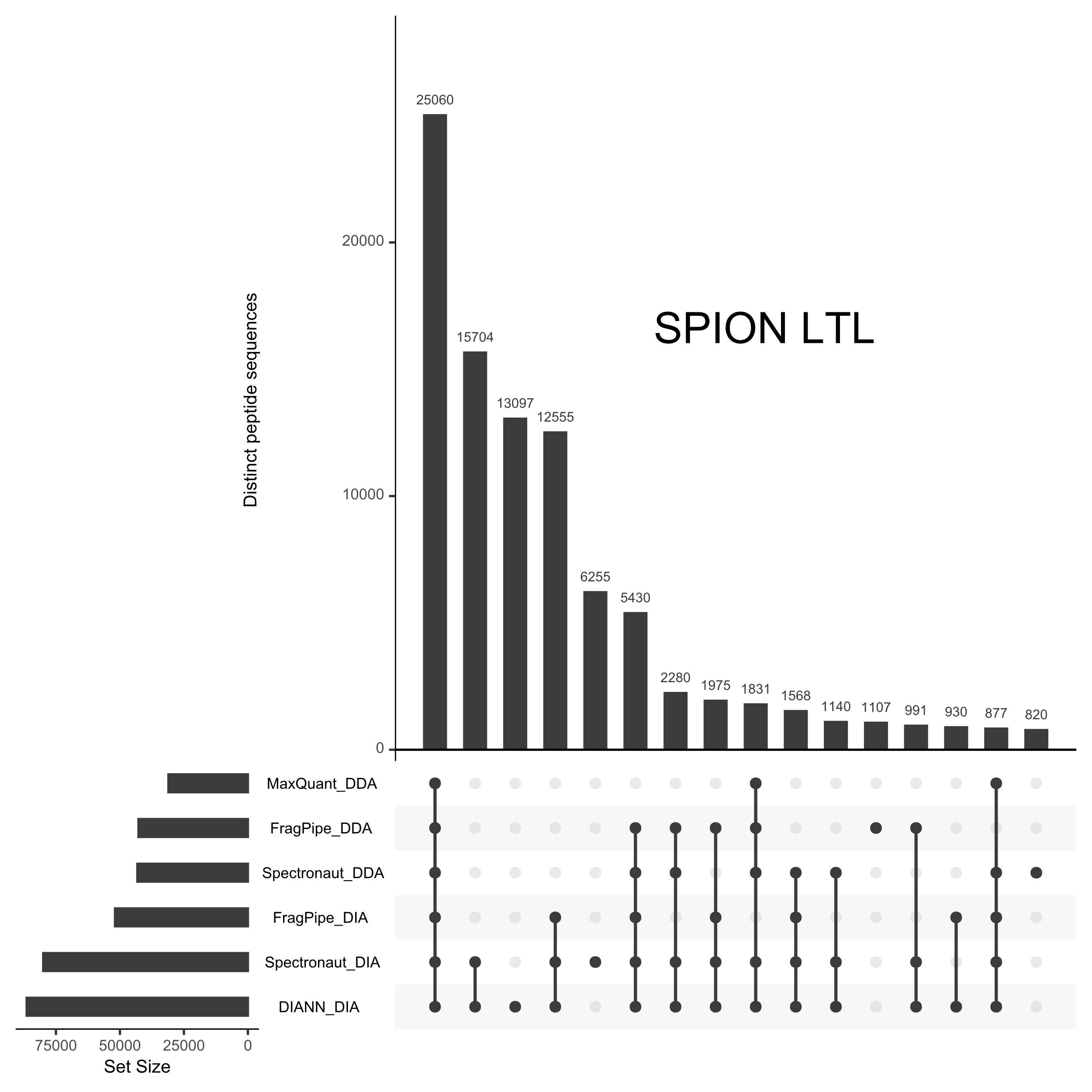


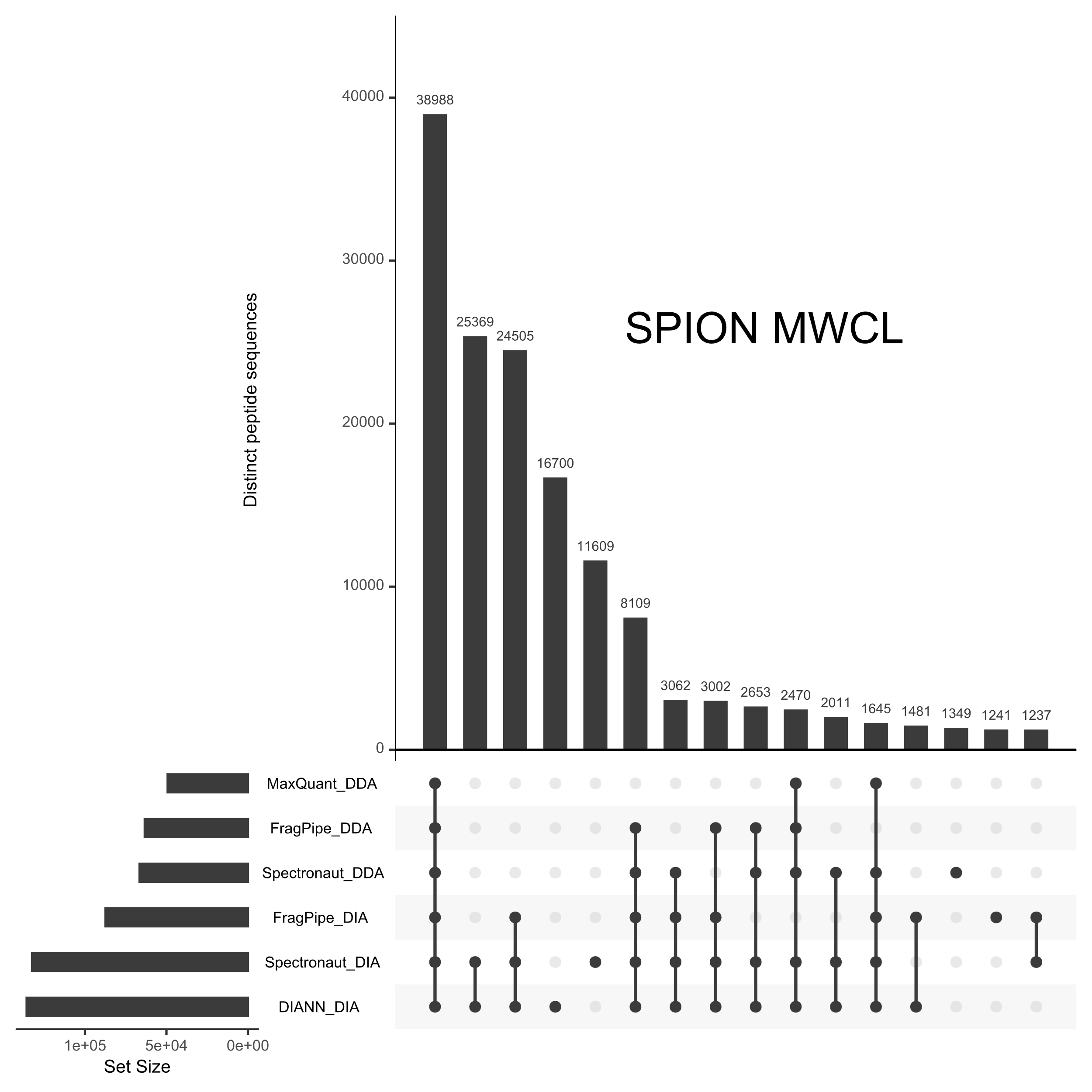


## Figure 1C: Thermo LINE-1


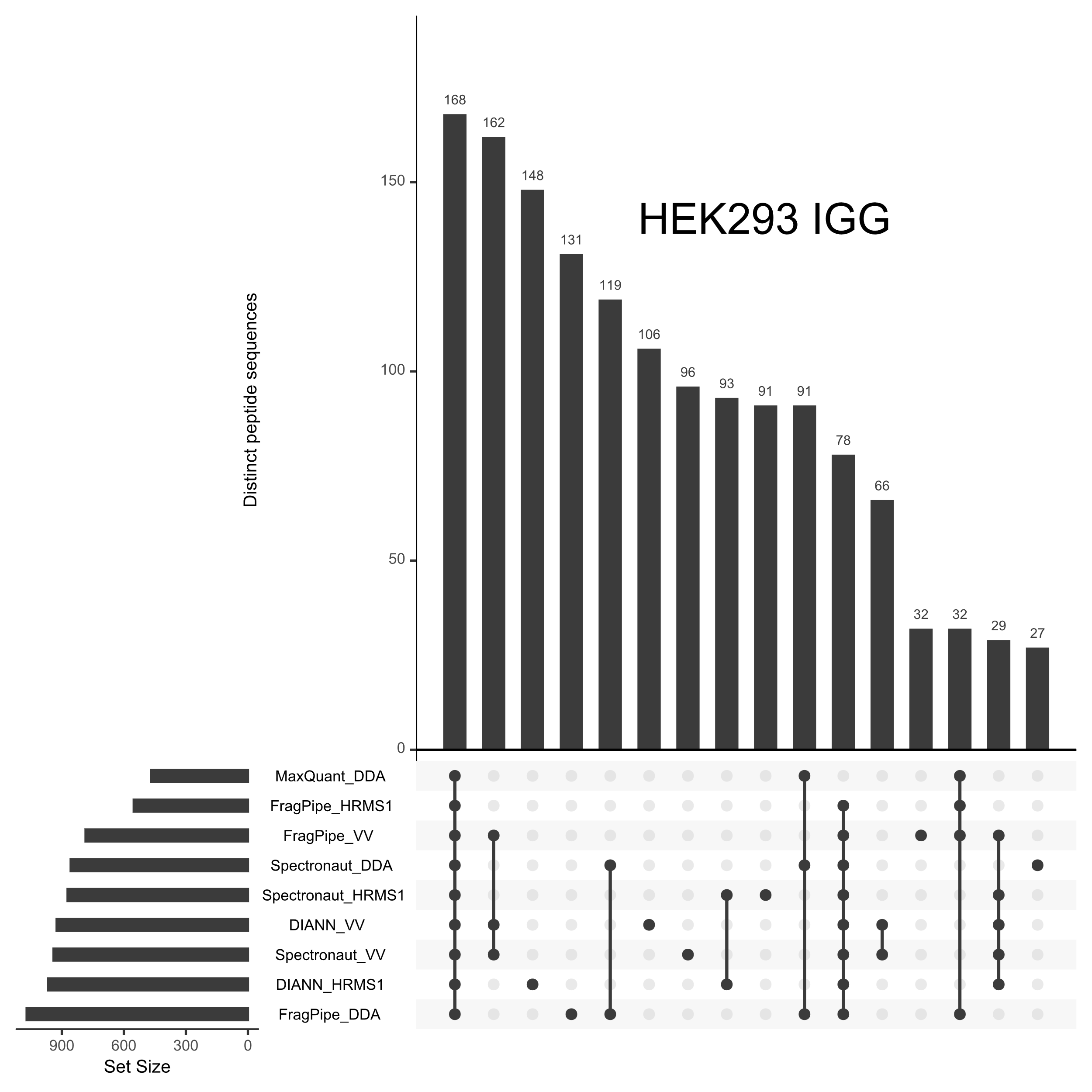


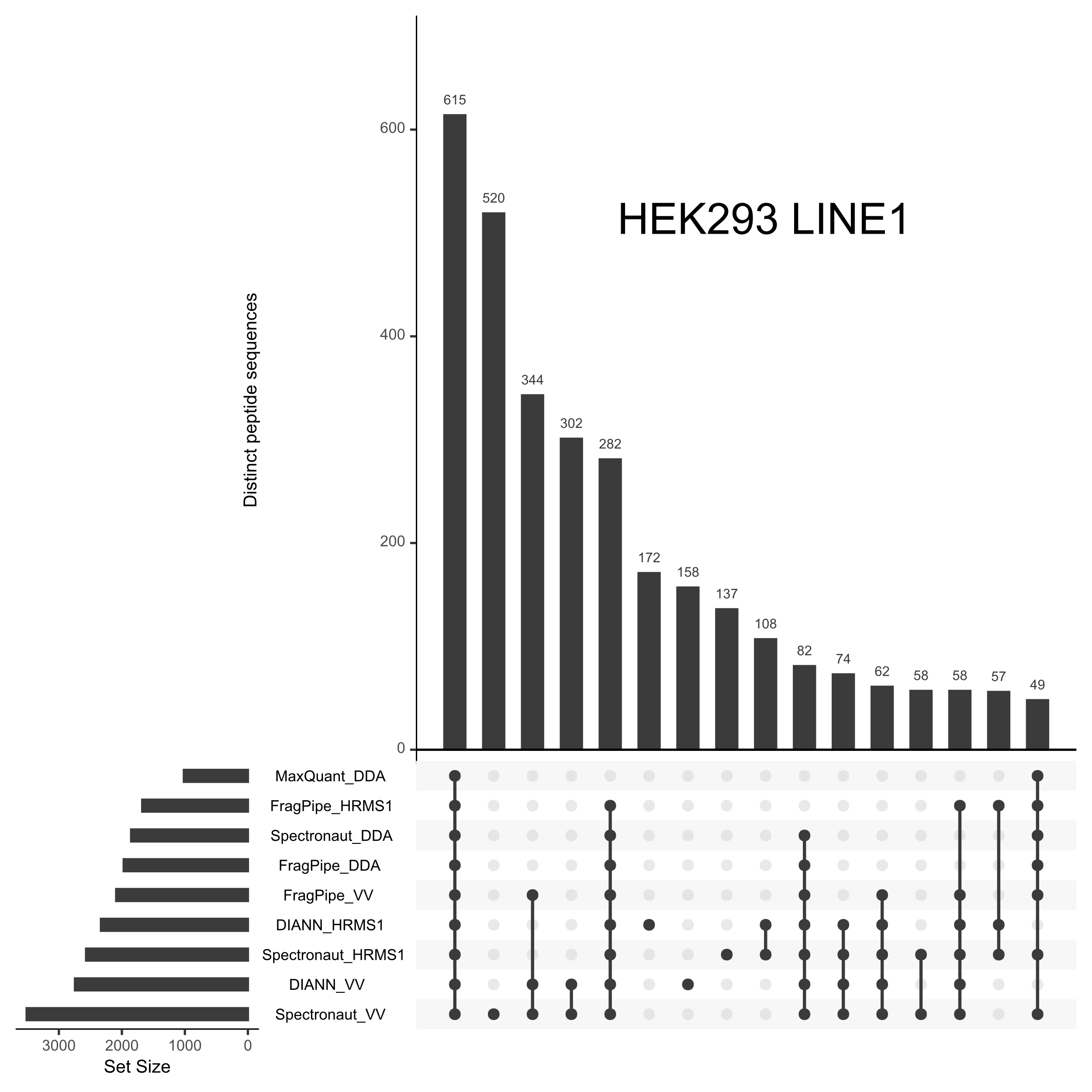


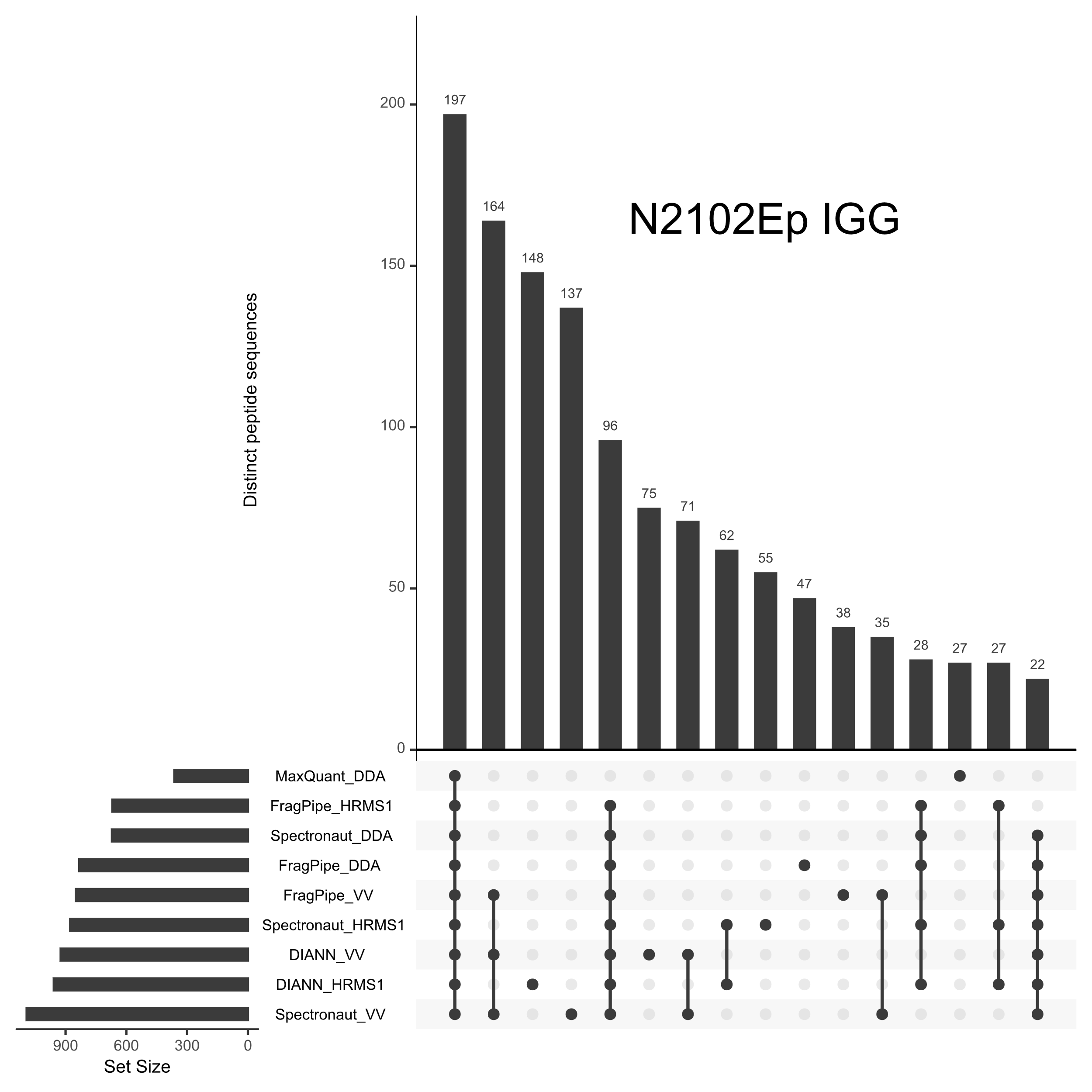


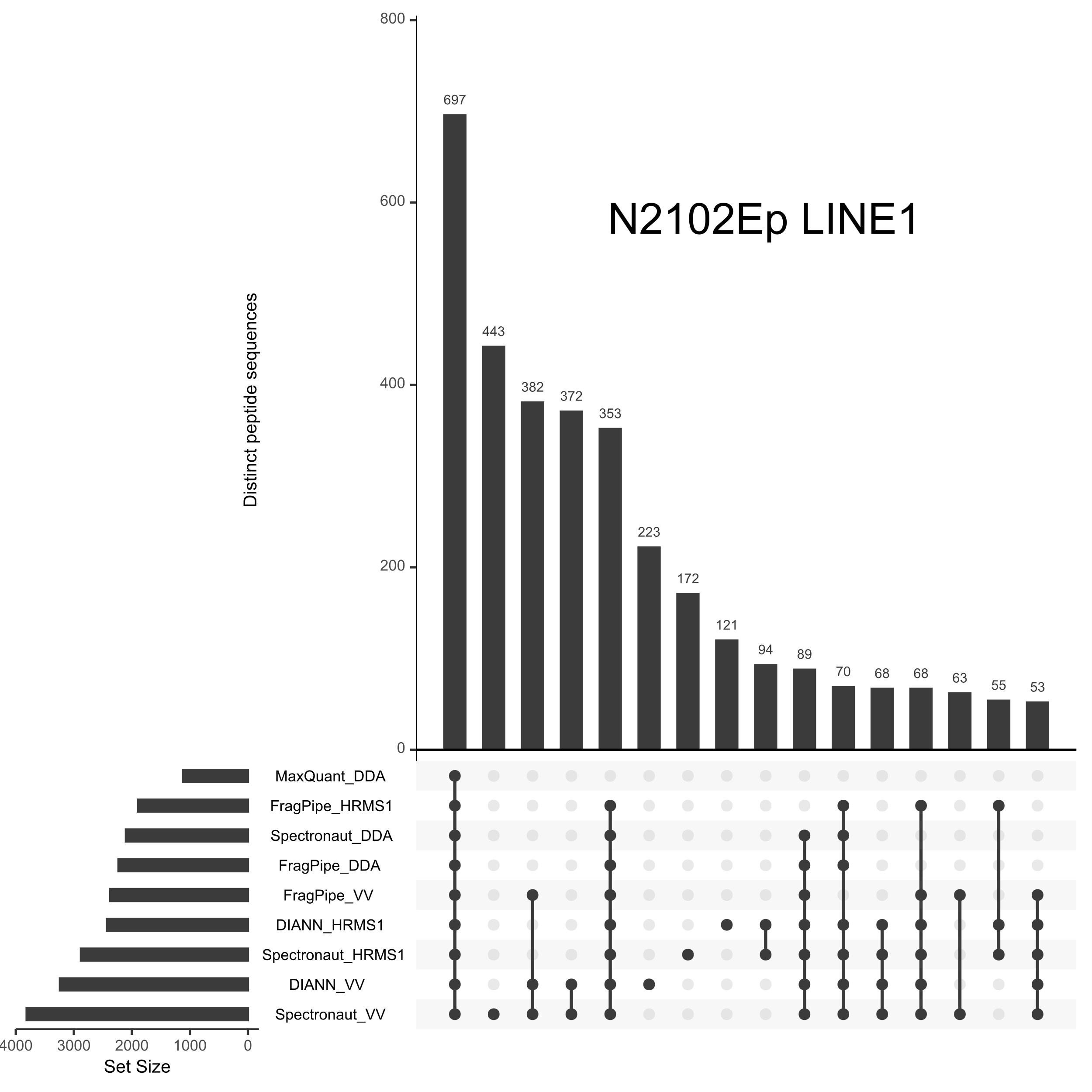


## Figure 1D: SCIEX RACK1


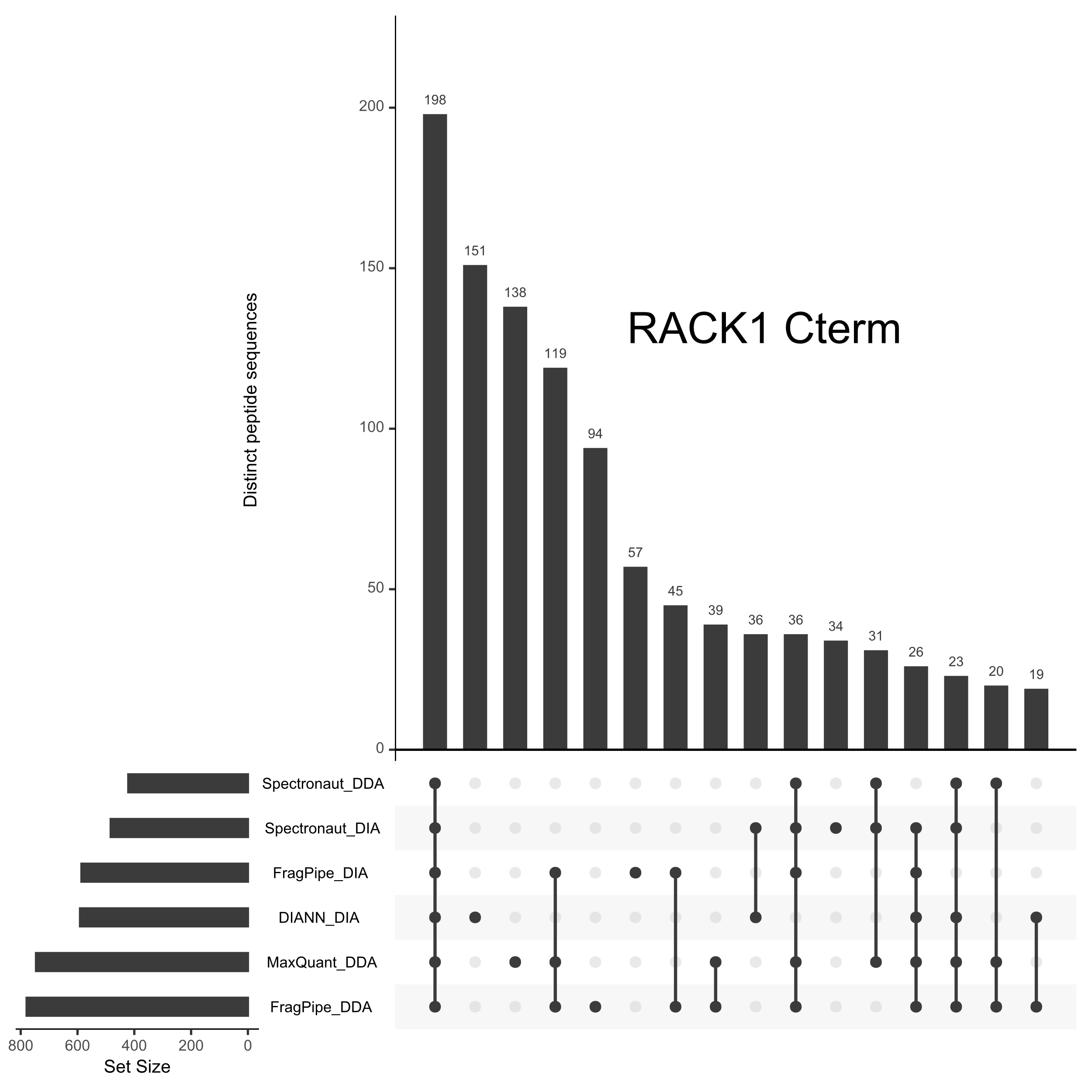


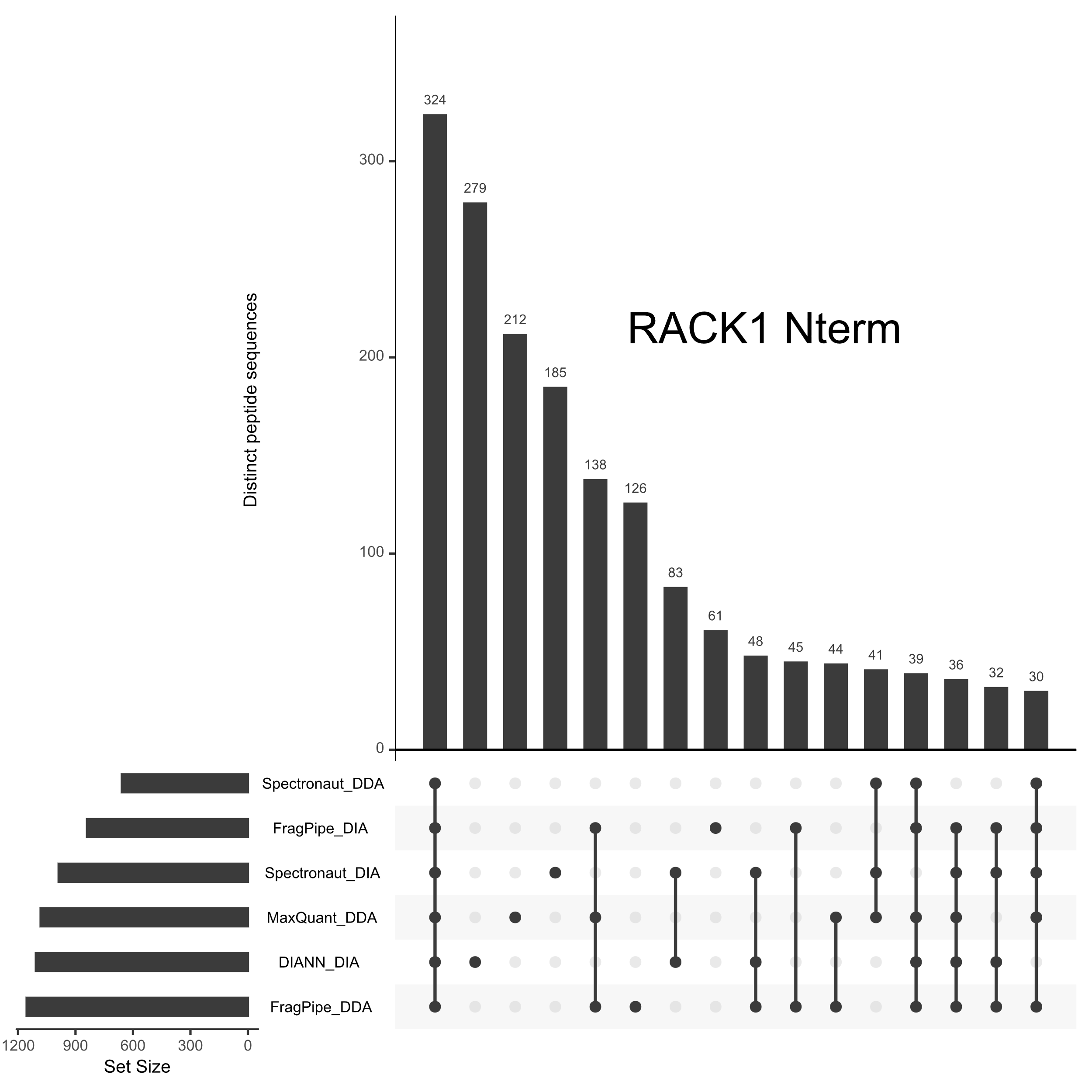


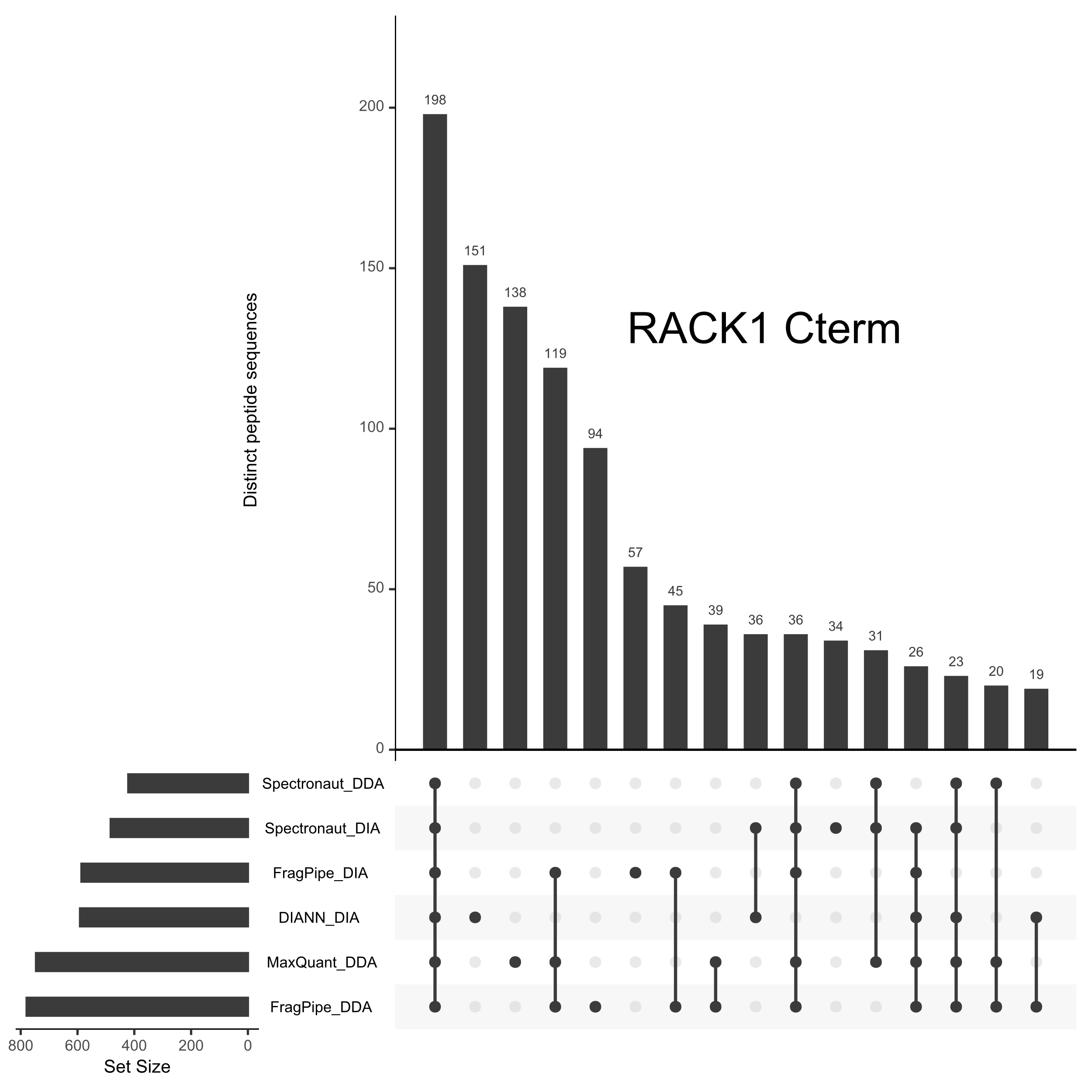


## Figure 1E: SCIEX ID4


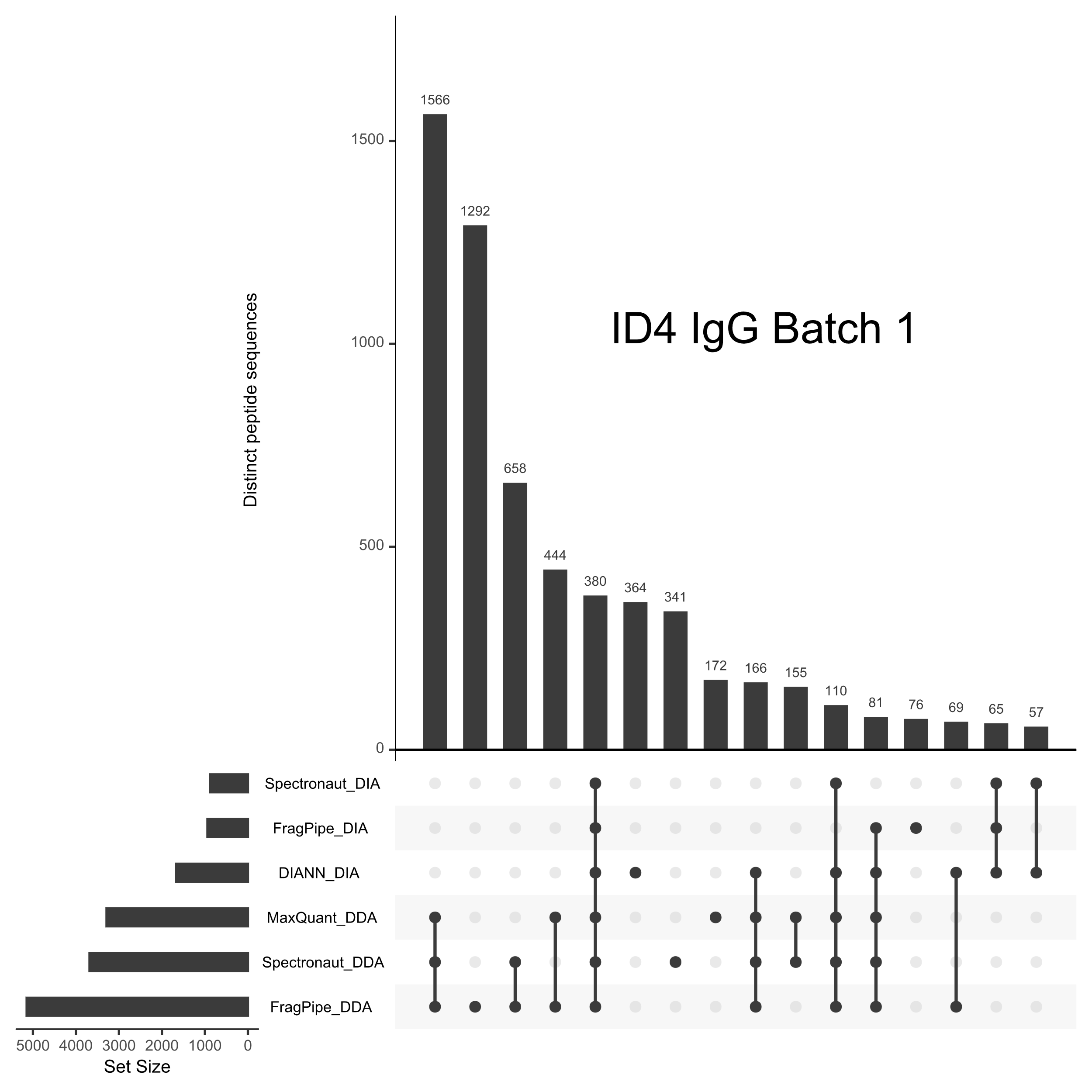


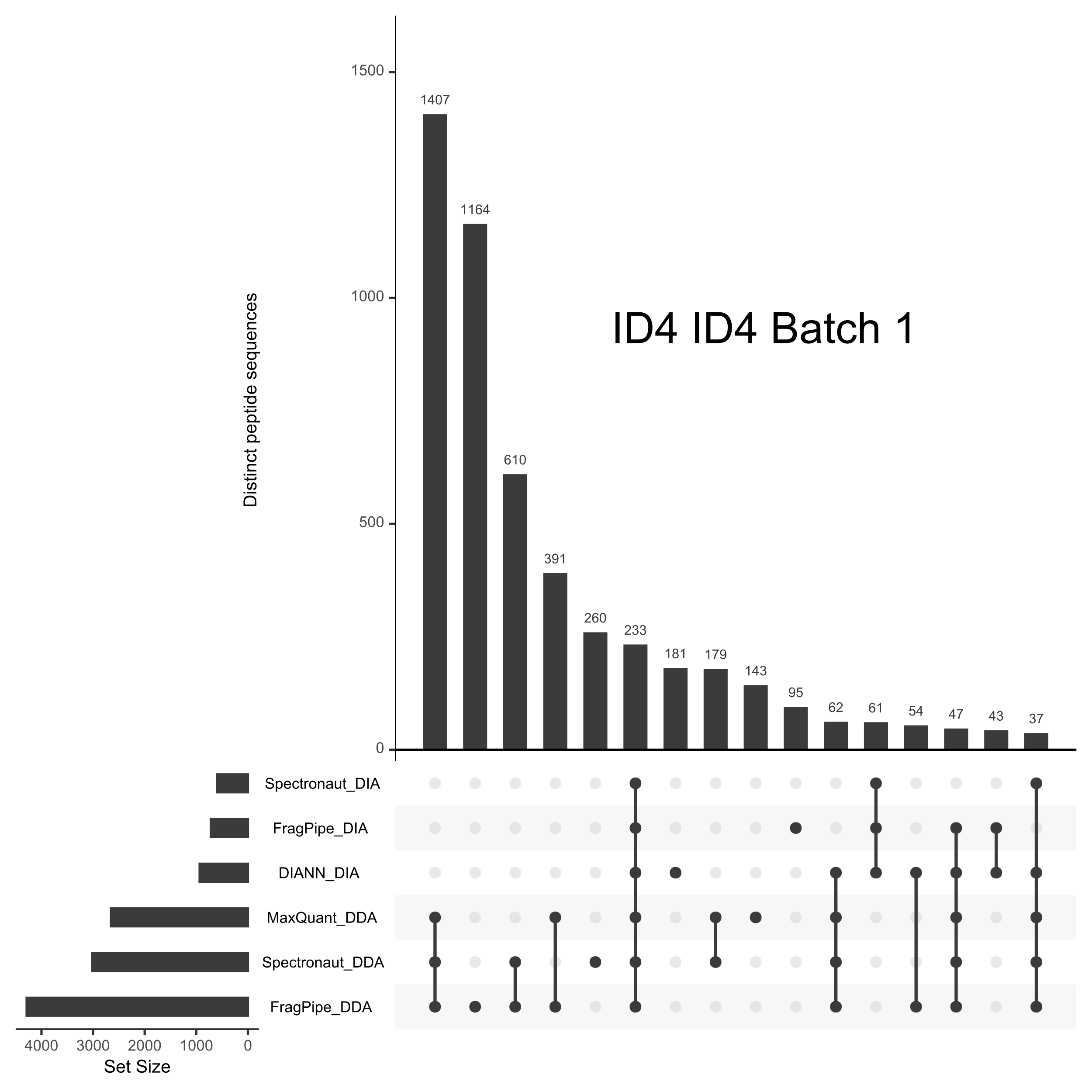


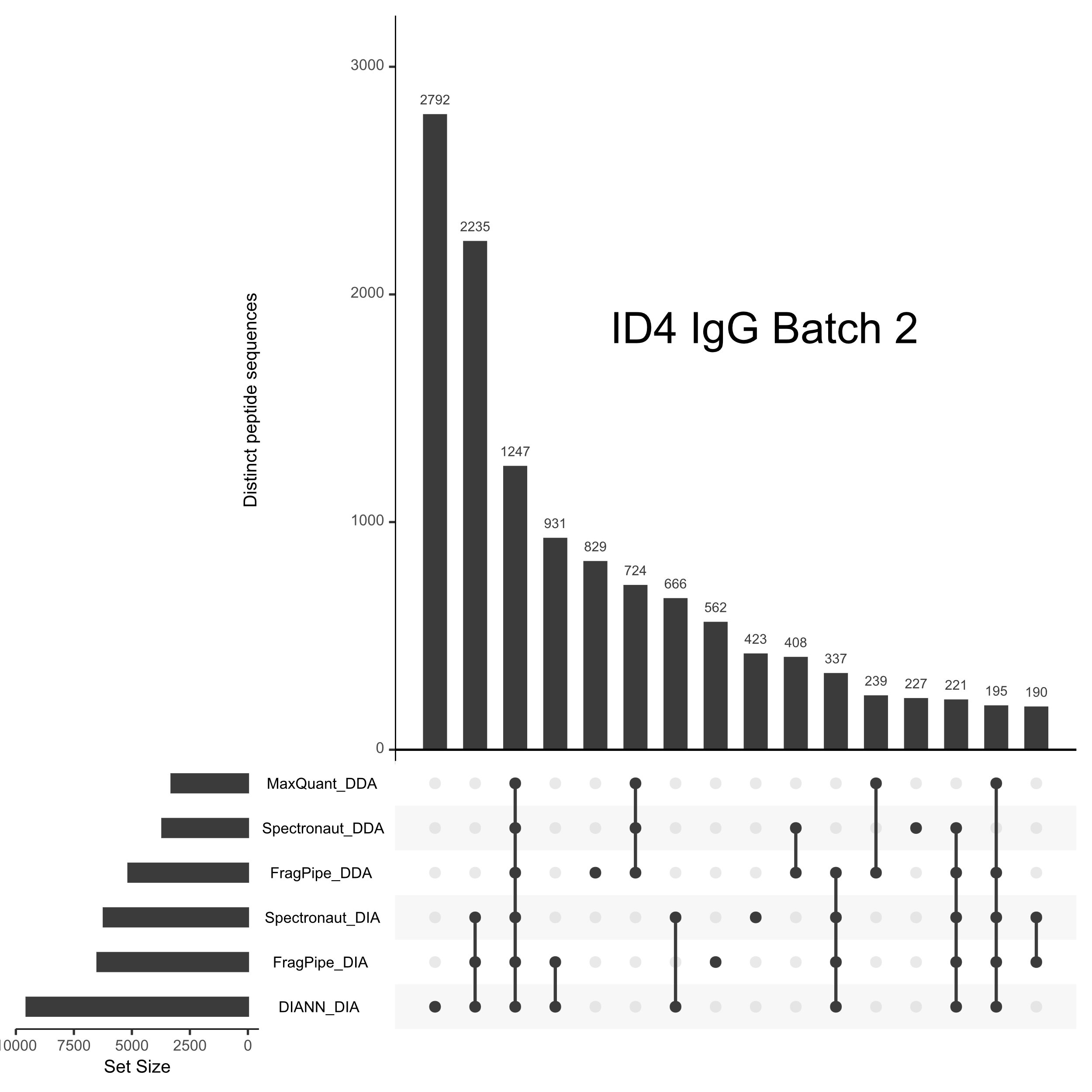


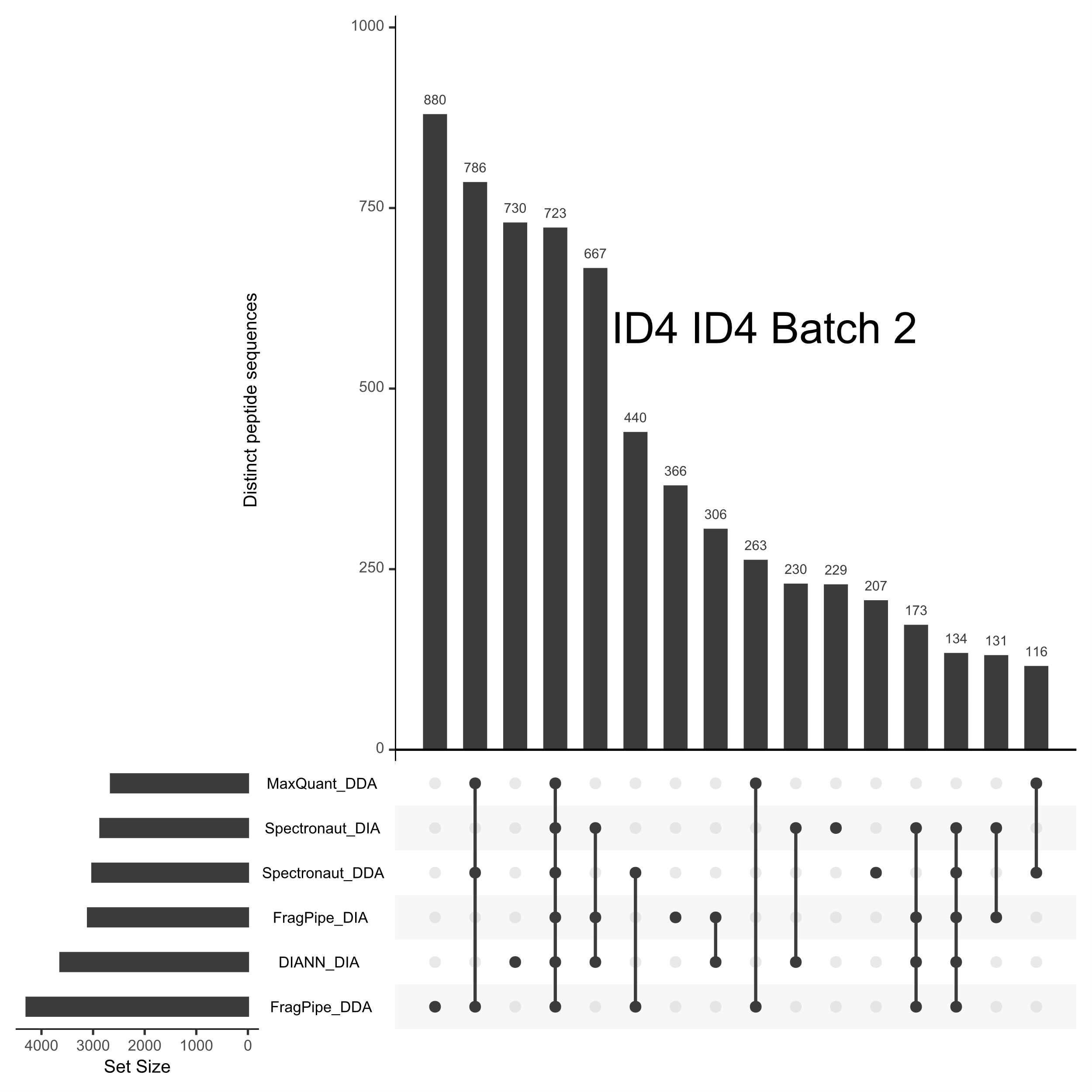


## Figure 1F: Bruker DUBs


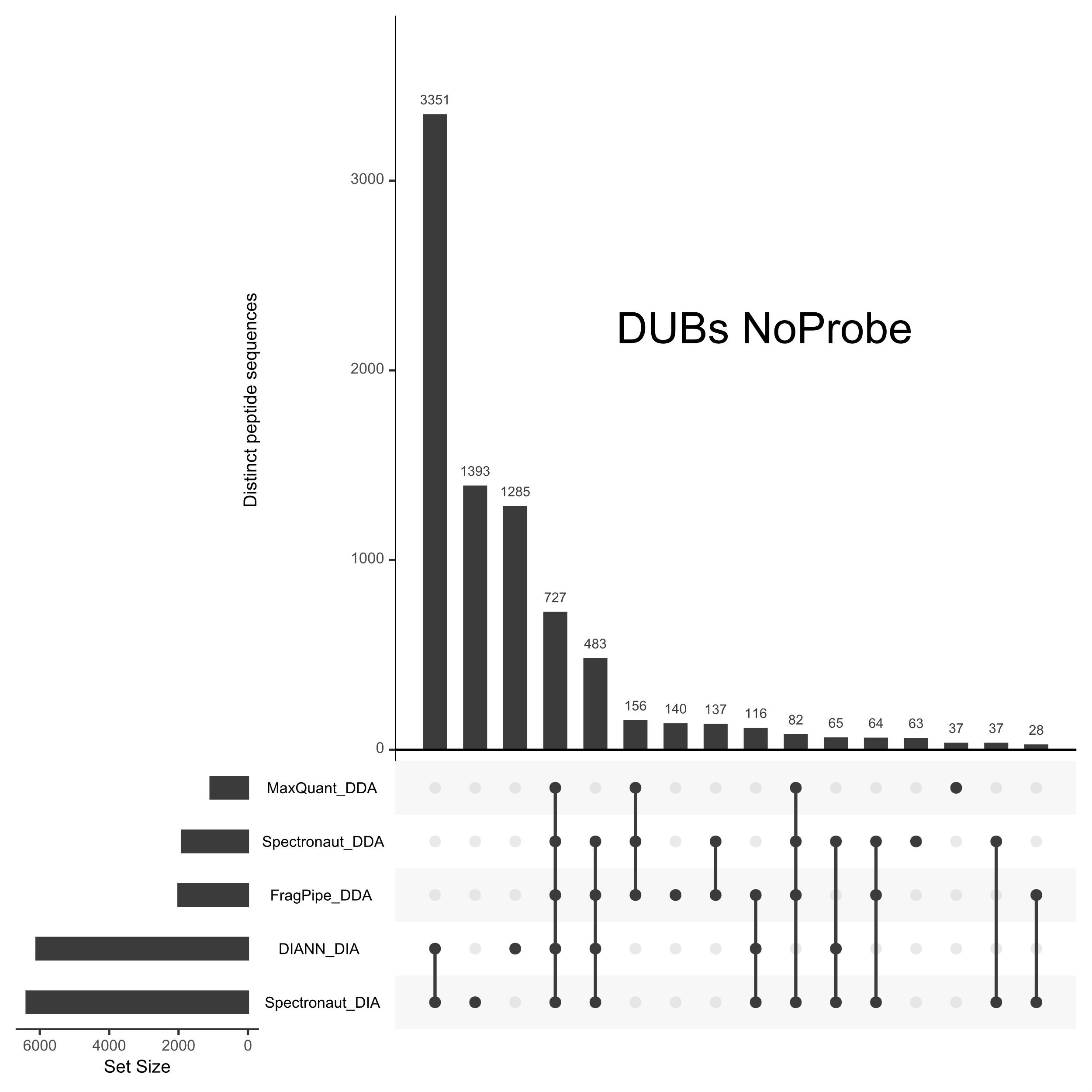


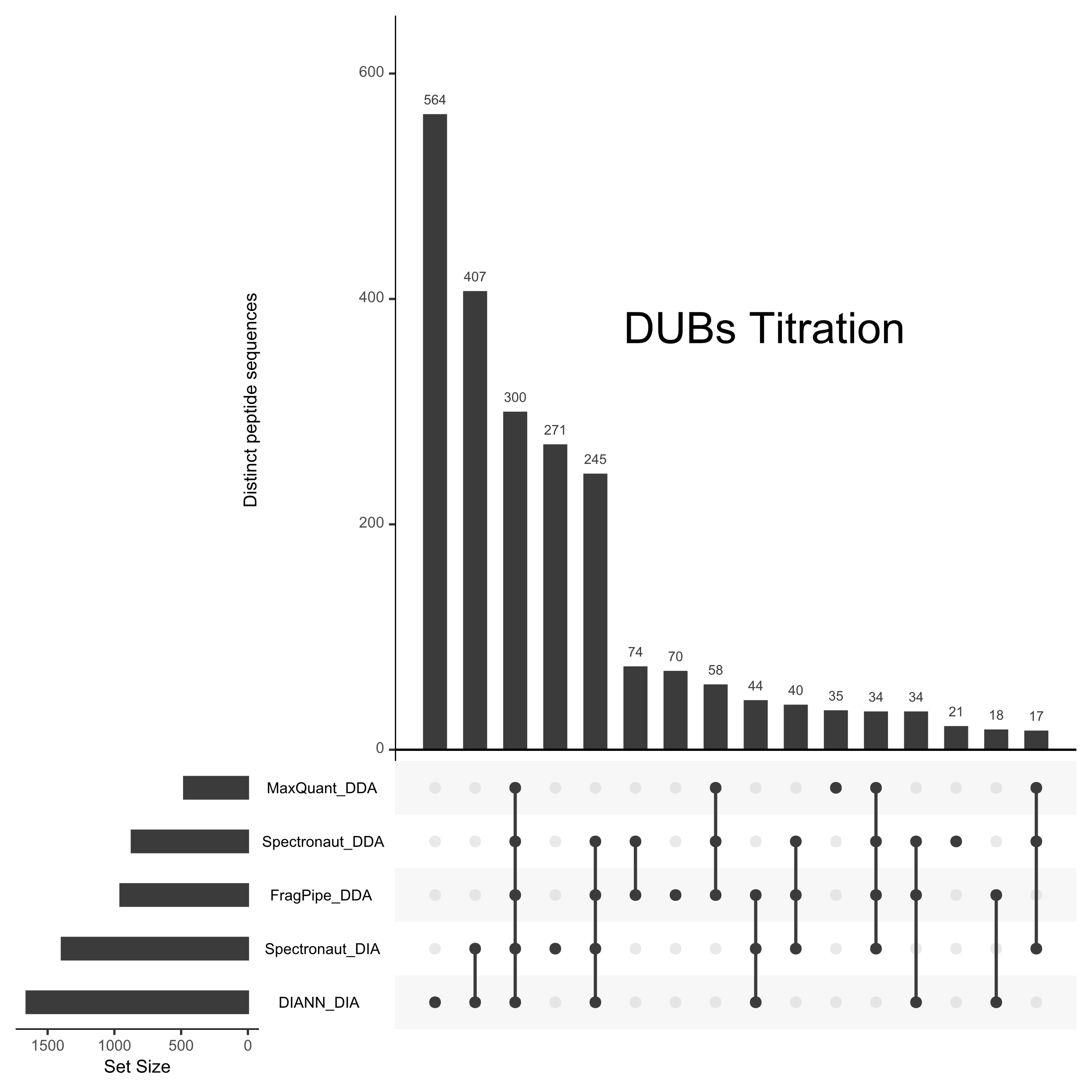


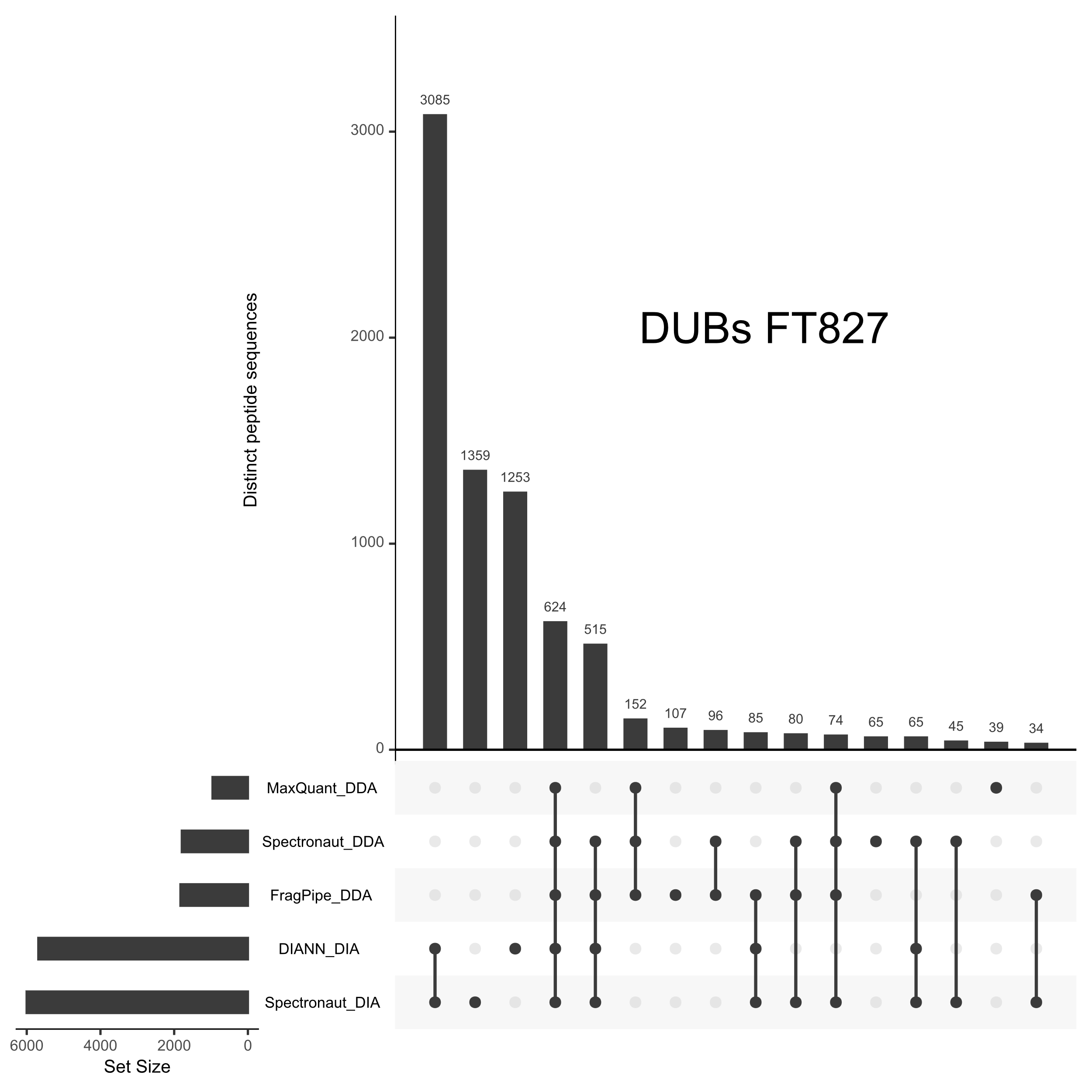


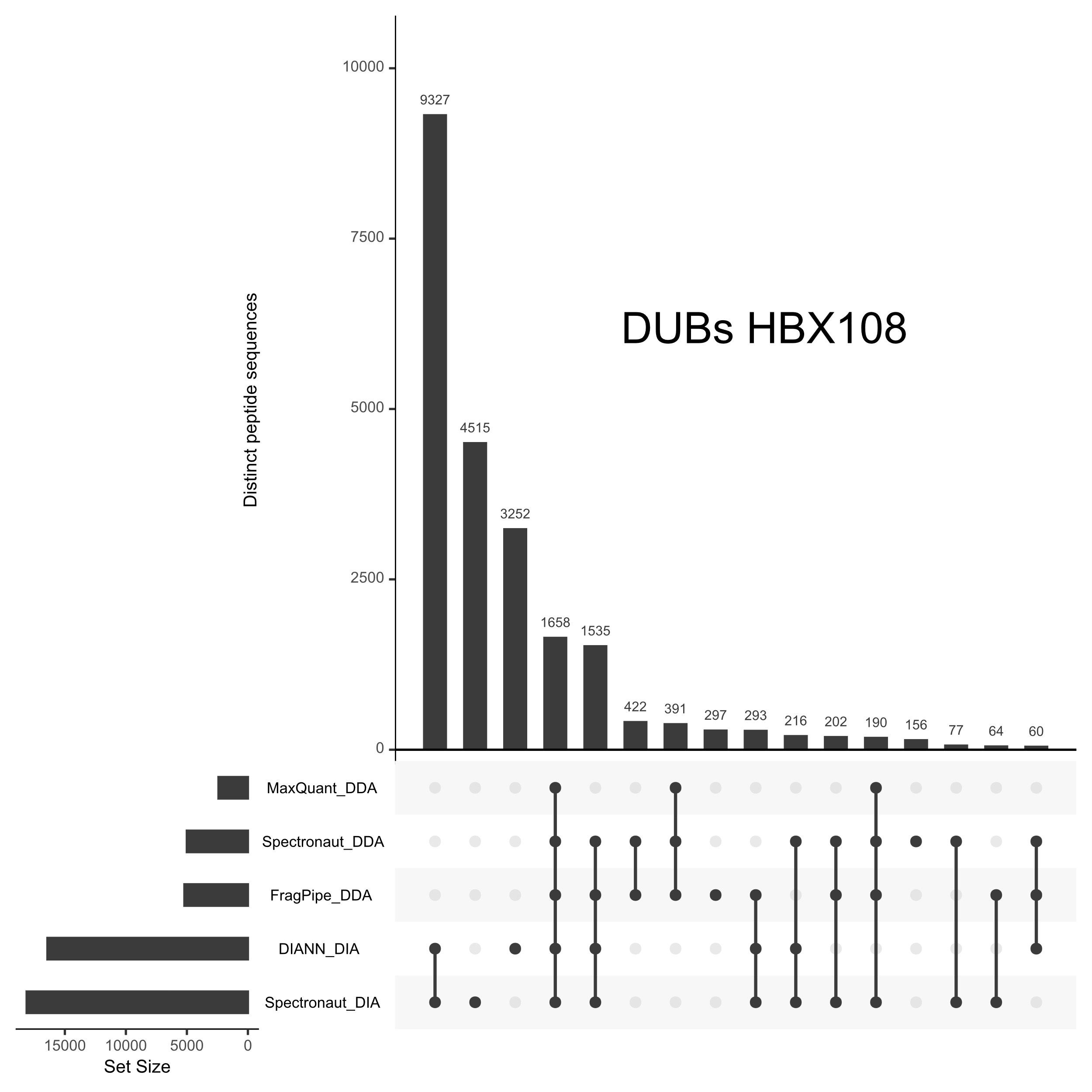


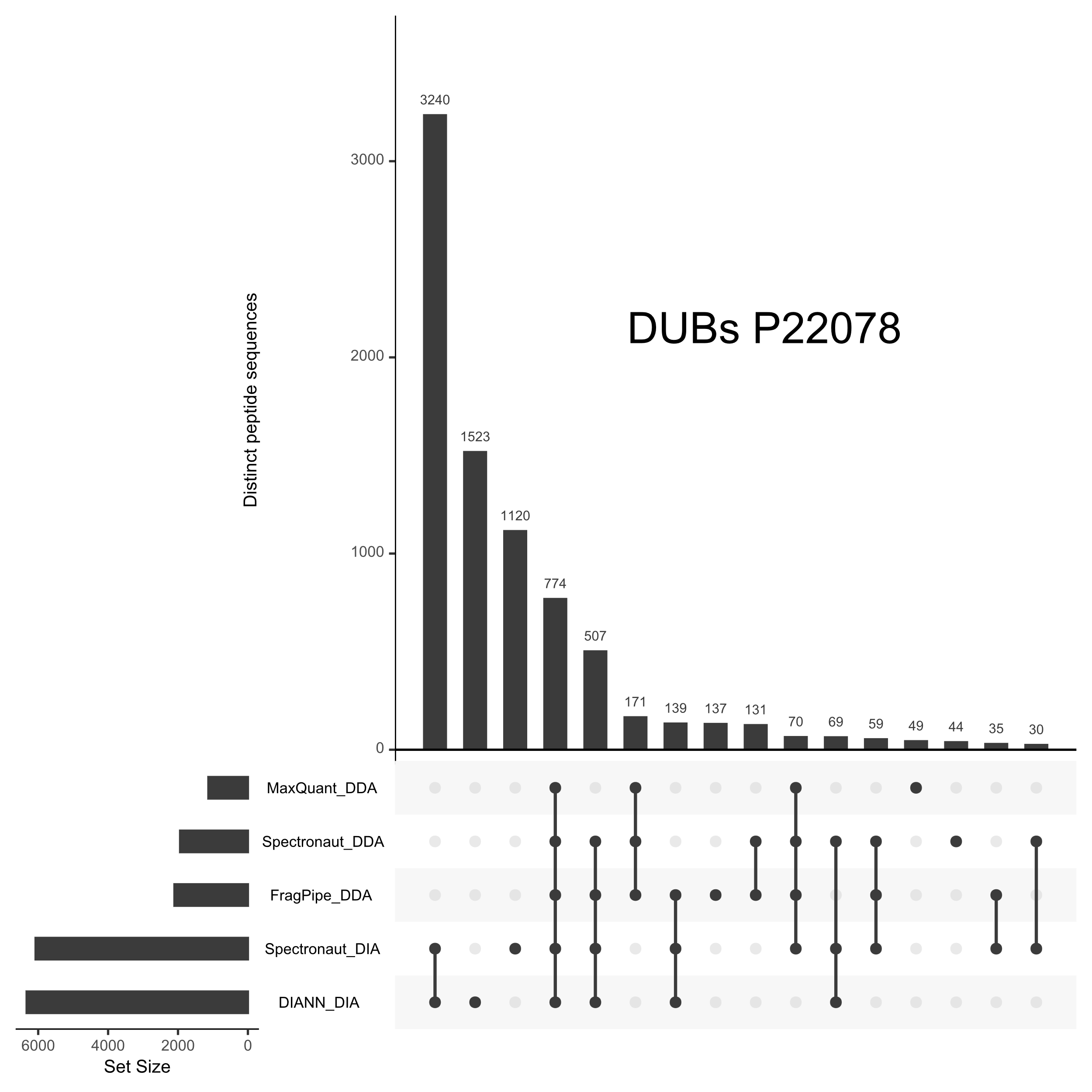


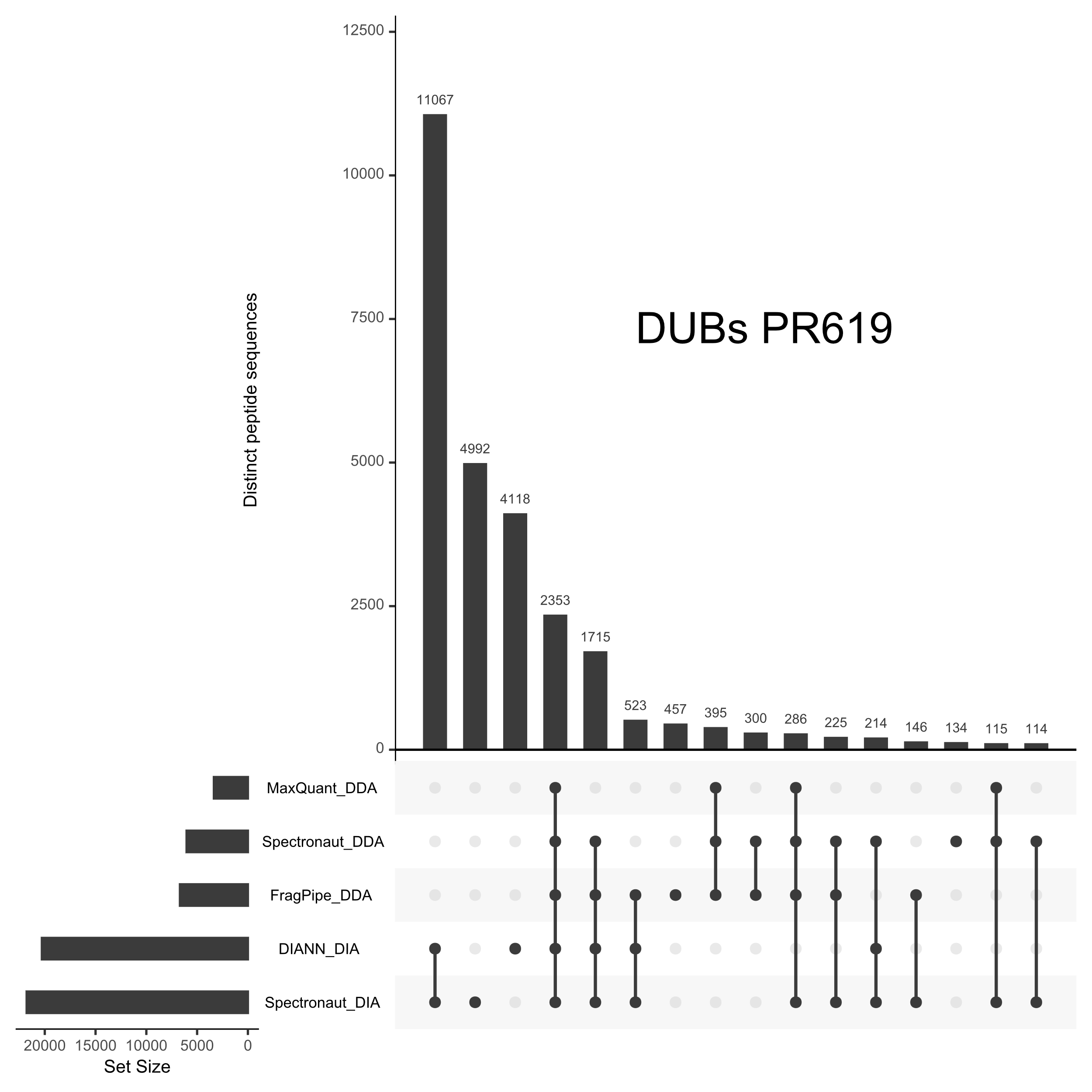


# Figure 2: Protein diversity for spectral libraries, any quantitation, and unanimous quantitation

A protein may be identified, with peptides added to a spectral library, and yet not quantified when that same library is used to quantify DIA experiments. These figures compare the number of proteins comprising a spectral library, the number of proteins for which any quantitative value was reported, and the number of proteins for which all quantitative values were reported.


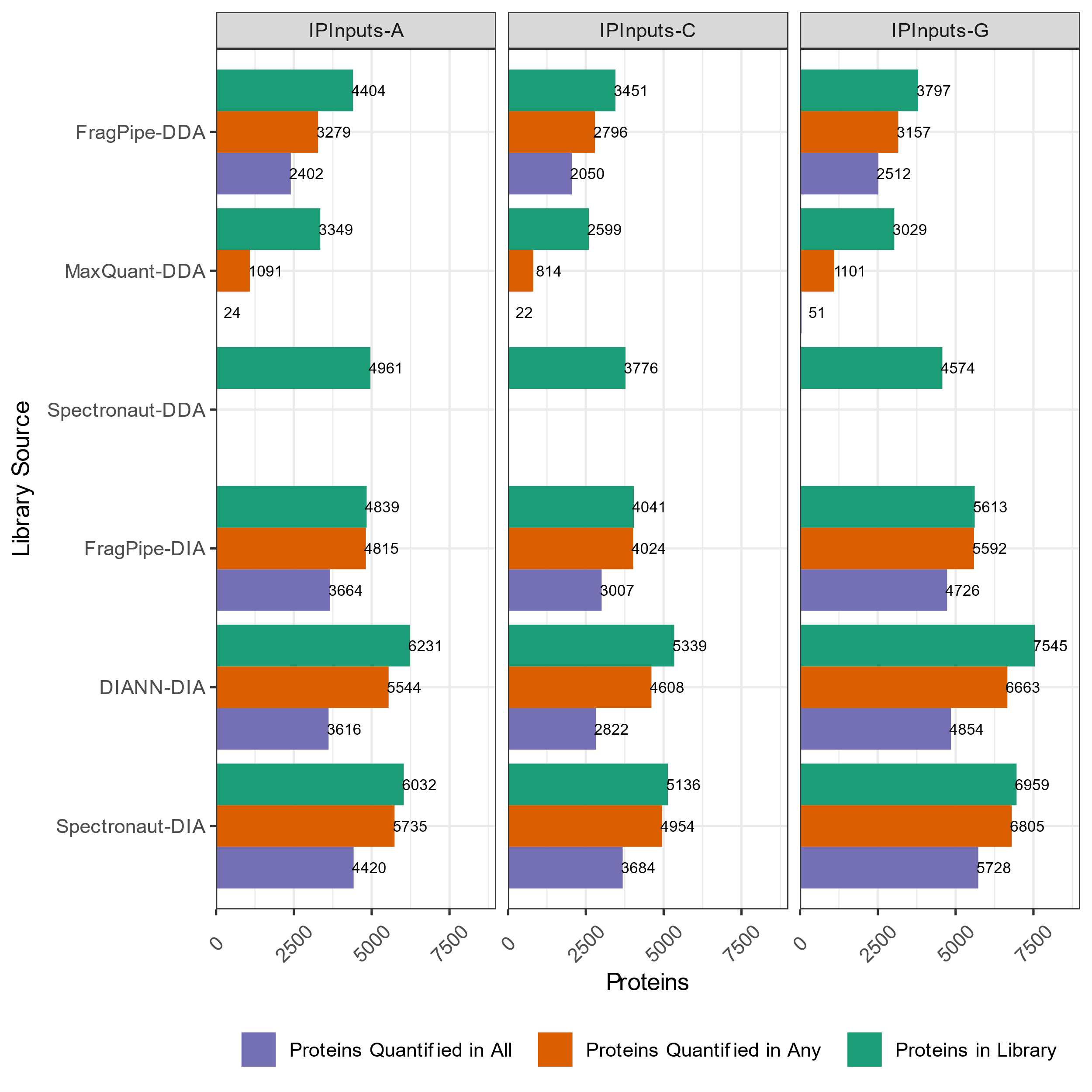


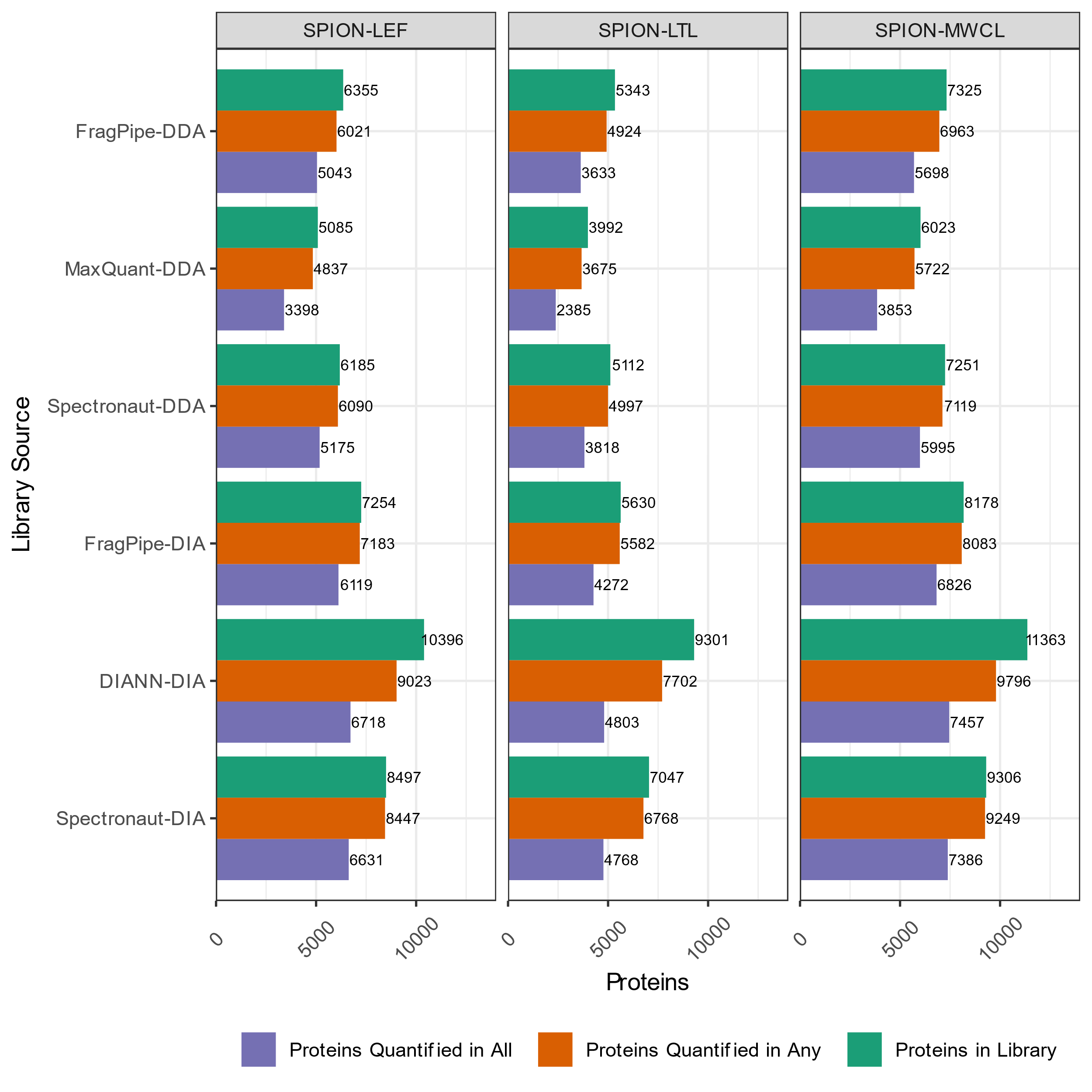


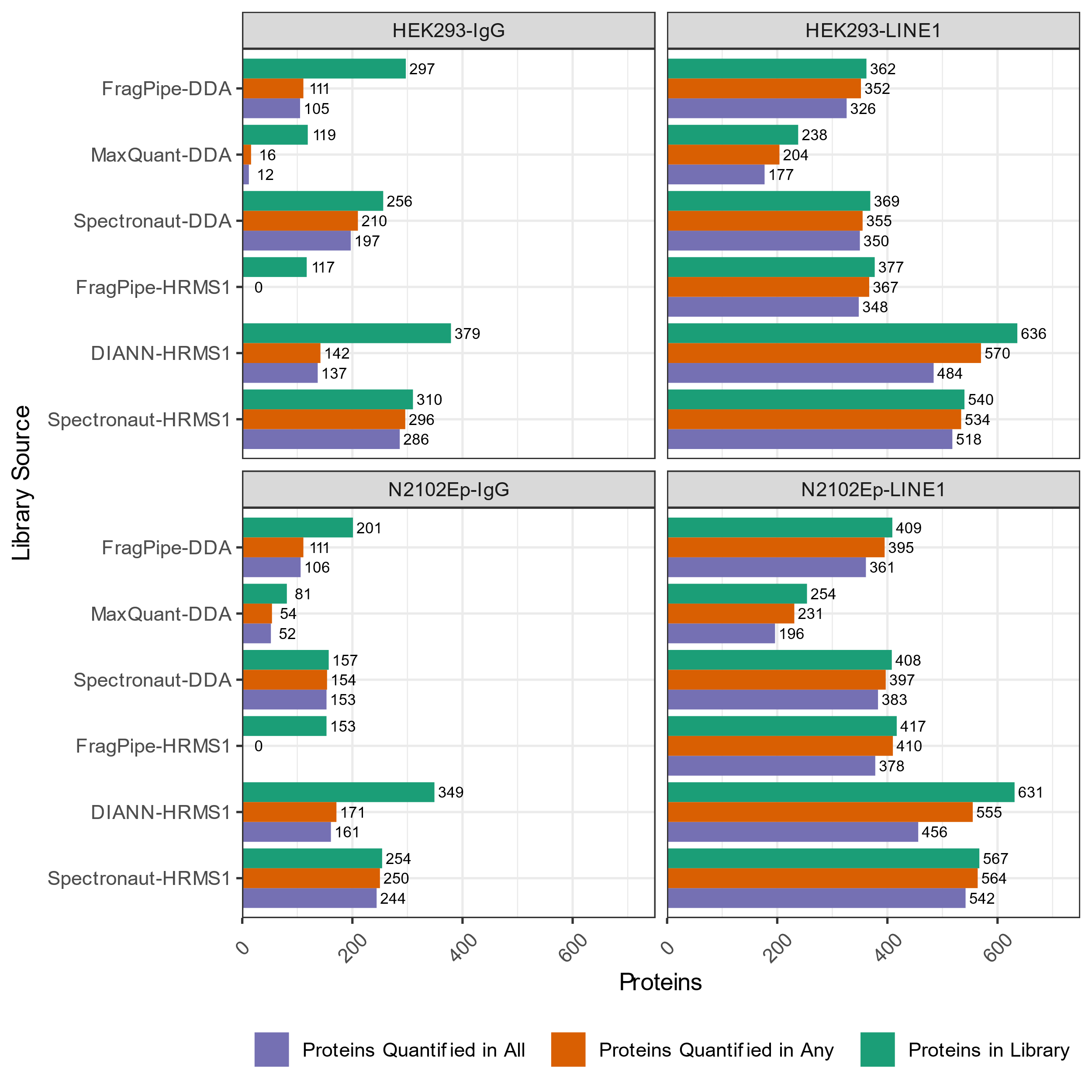


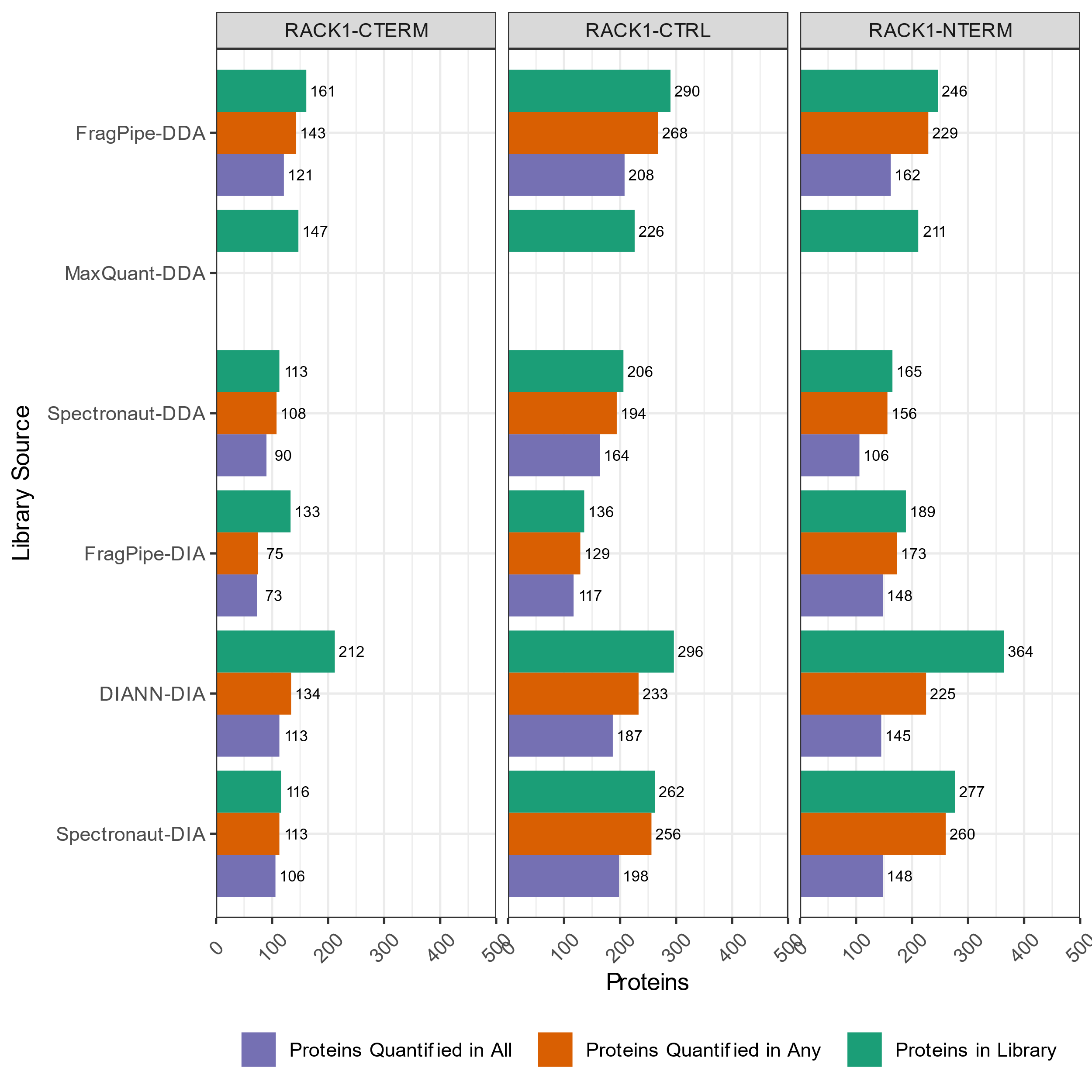


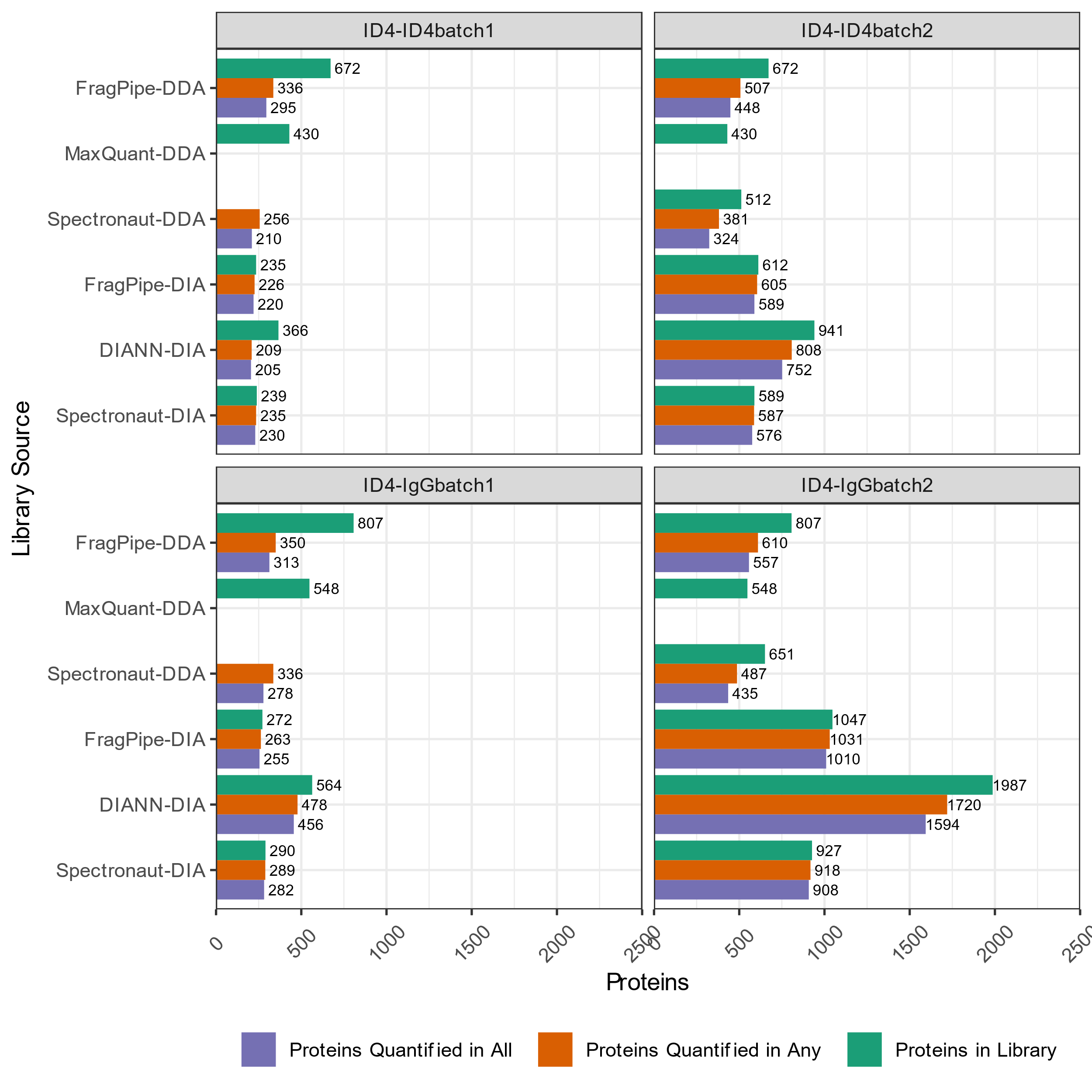


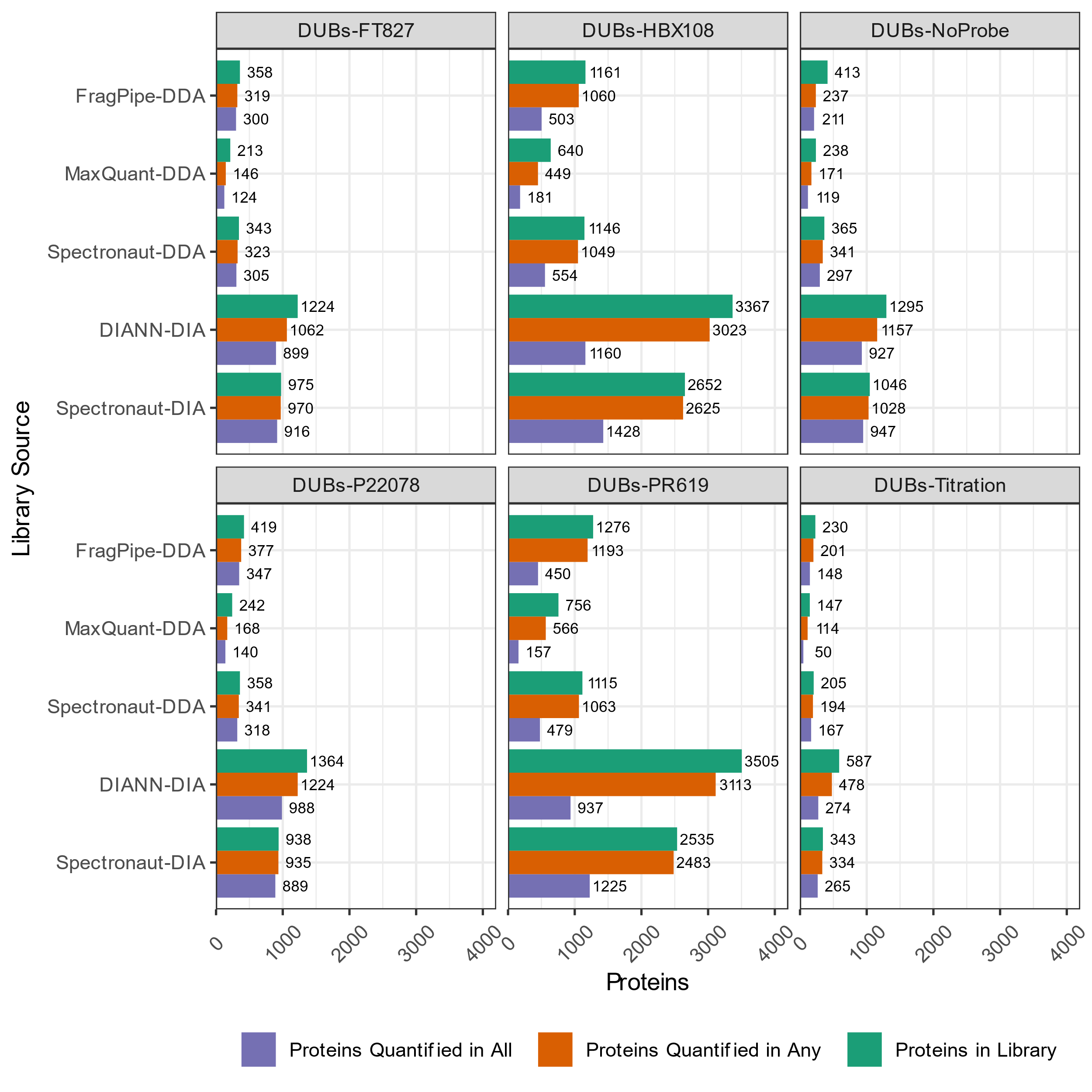


# Figure 3: Coefficients of Variation for intense, medium intensity, and low-intensity proteins

We expected to find a relationship between the intensity reported for a protein and the coefficient of variation for its measurements. We expected that variation would be smaller in relation to intensity when the magnitude of intensity is large. We expected variation would be larger in relation to intensity when the signal-to-noise is low. We subdivided the proteins to fifths, with the most intense proteins in the top quintile and the least intense proteins in the bottom quintile. Proteins in the middle set are the middle-of-the-road intensities. We can compute CV values for every protein, and we report the median CV for the top, middle, and bottom quintile of proteins, largely supporting our expectation of intensity and CV’s relationship. The most dominant effect observed in the graphs below, however, is that MaxQuant DDA libraries that are used to quantify DIA experiments in MaxDIA give incomparably larger CV values than the other software workflows.


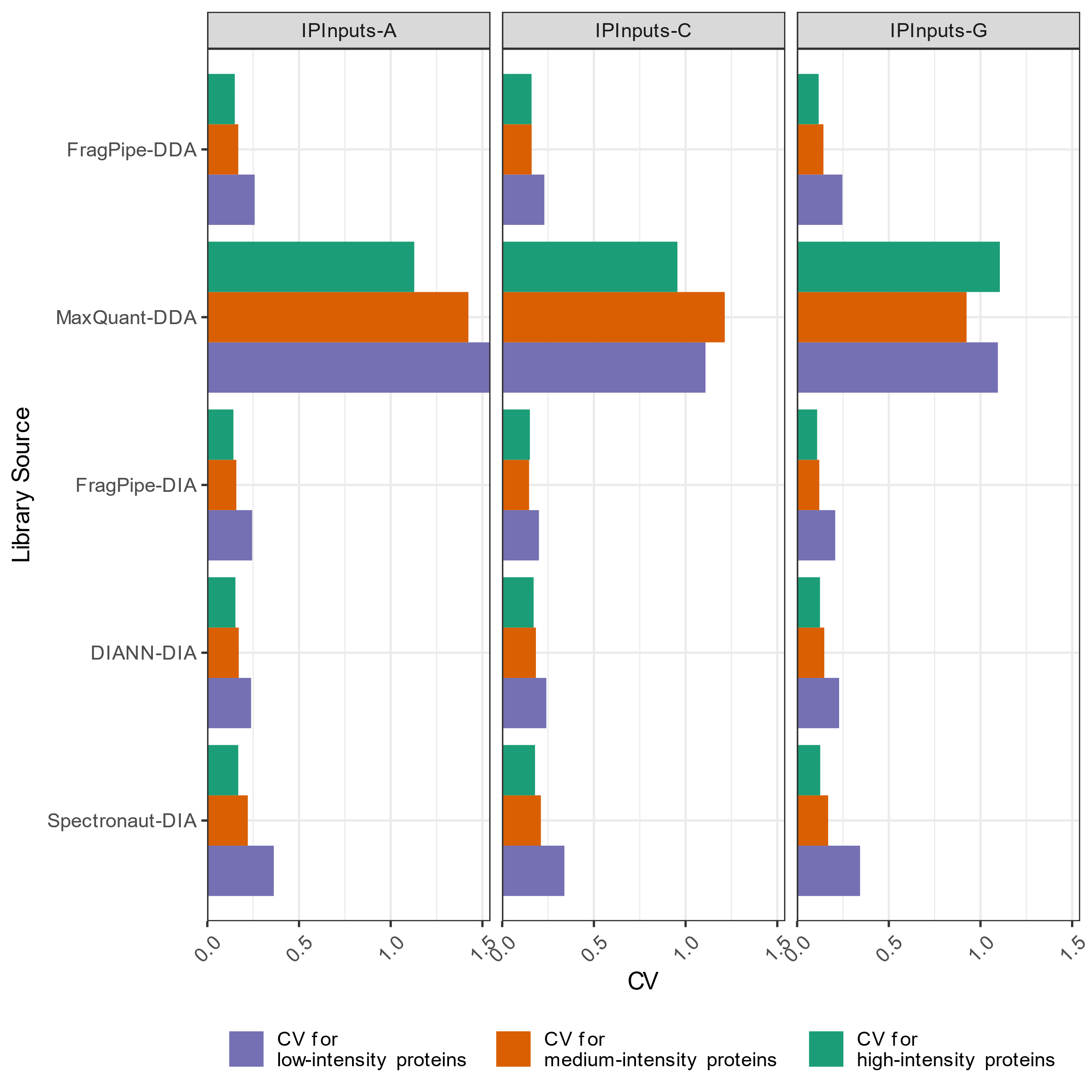


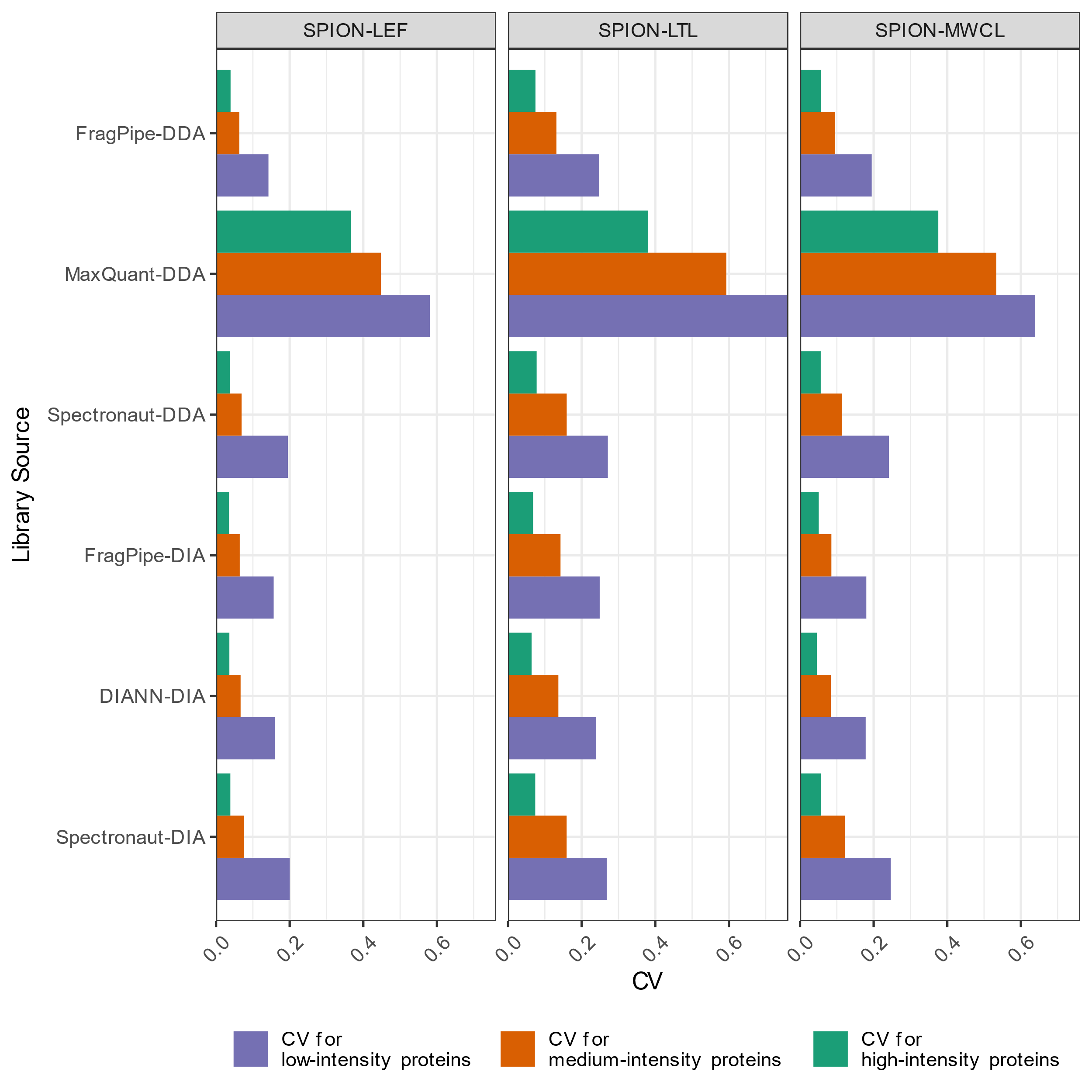


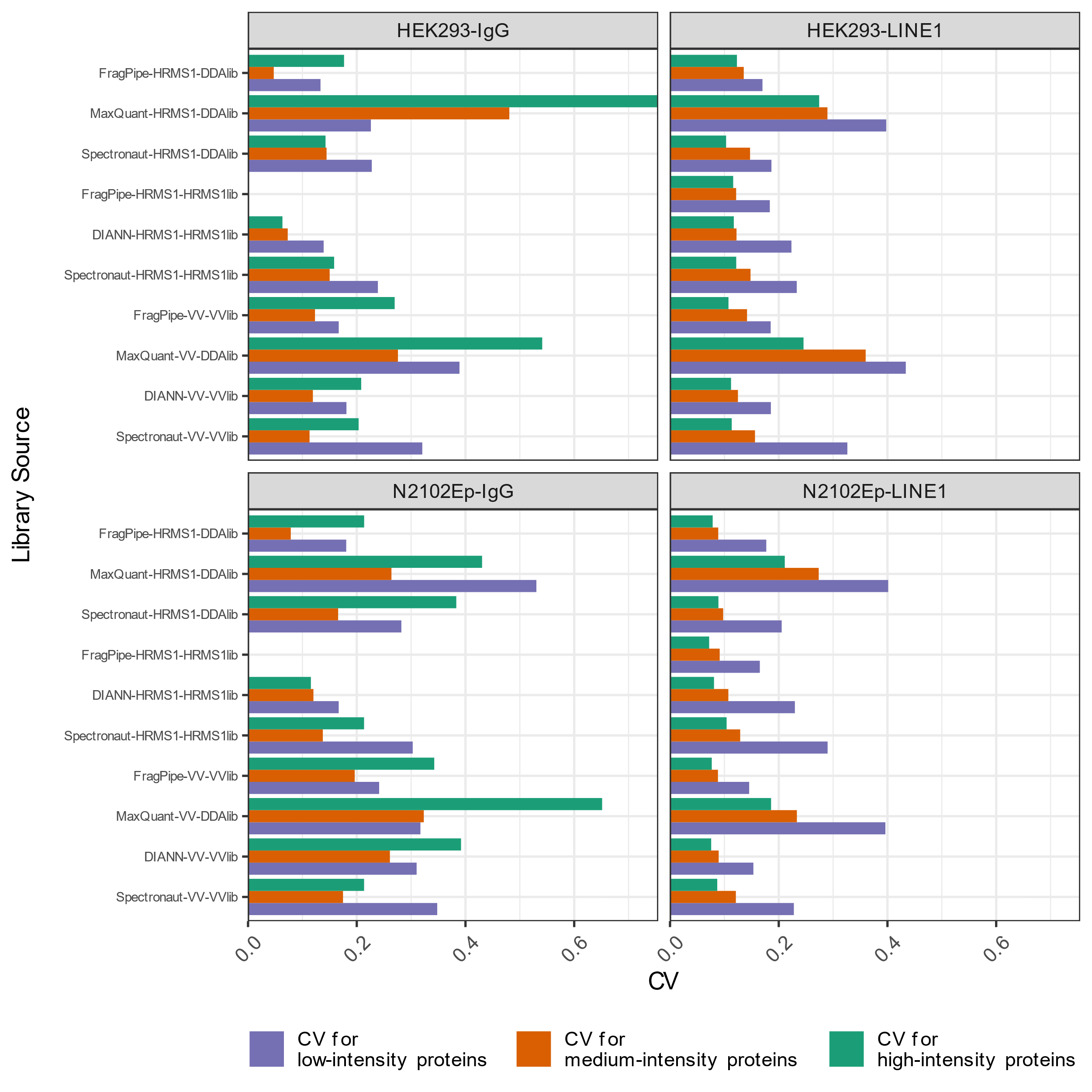


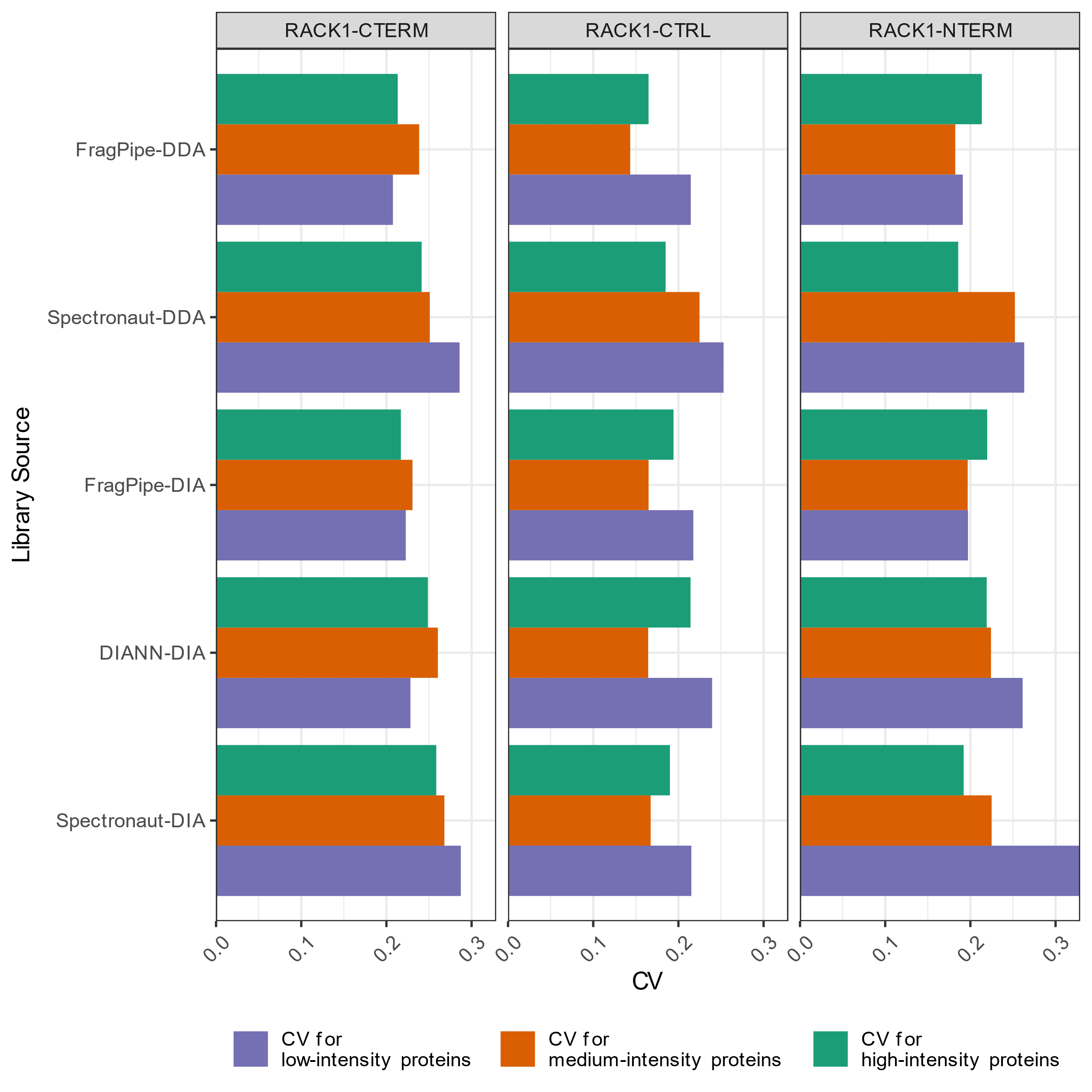


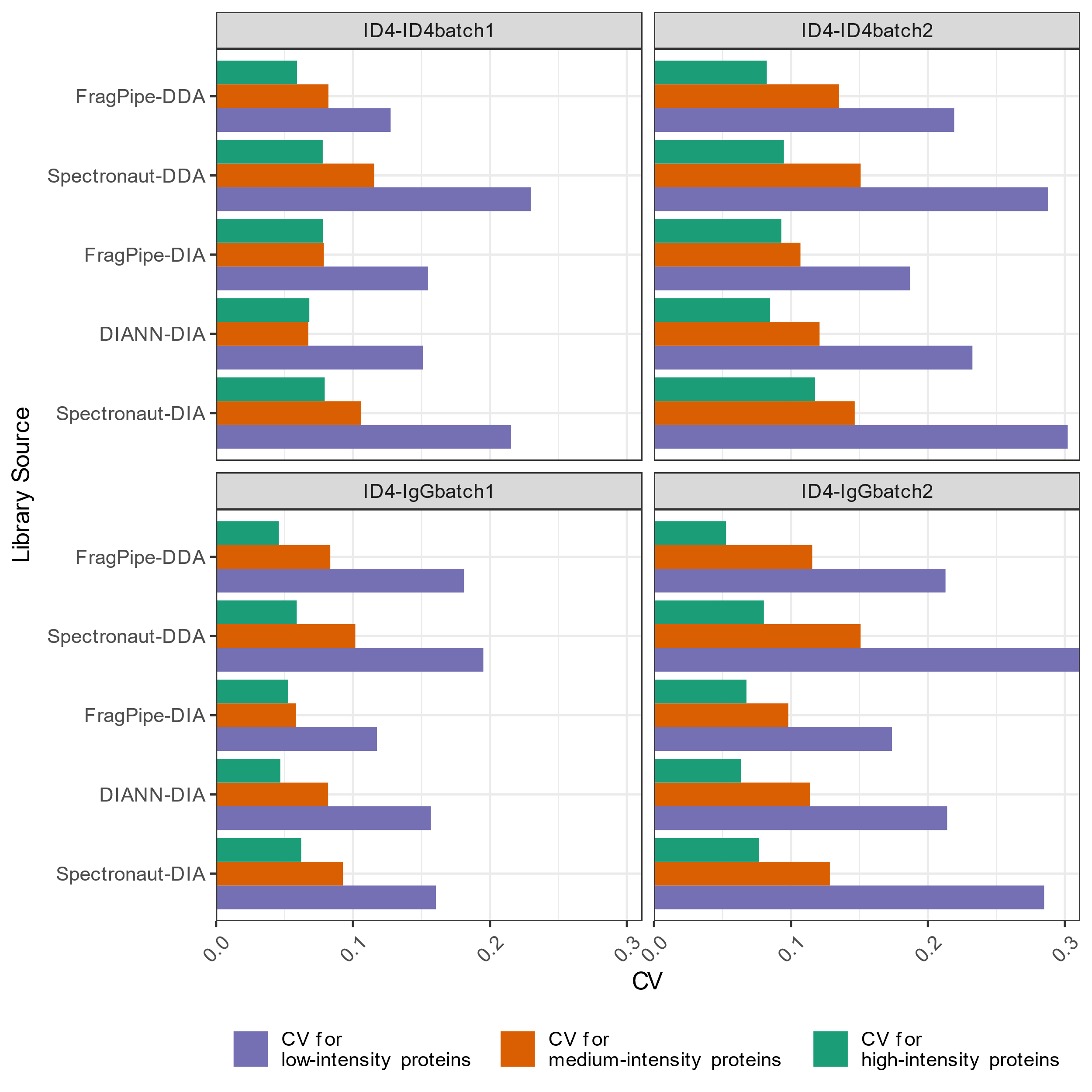


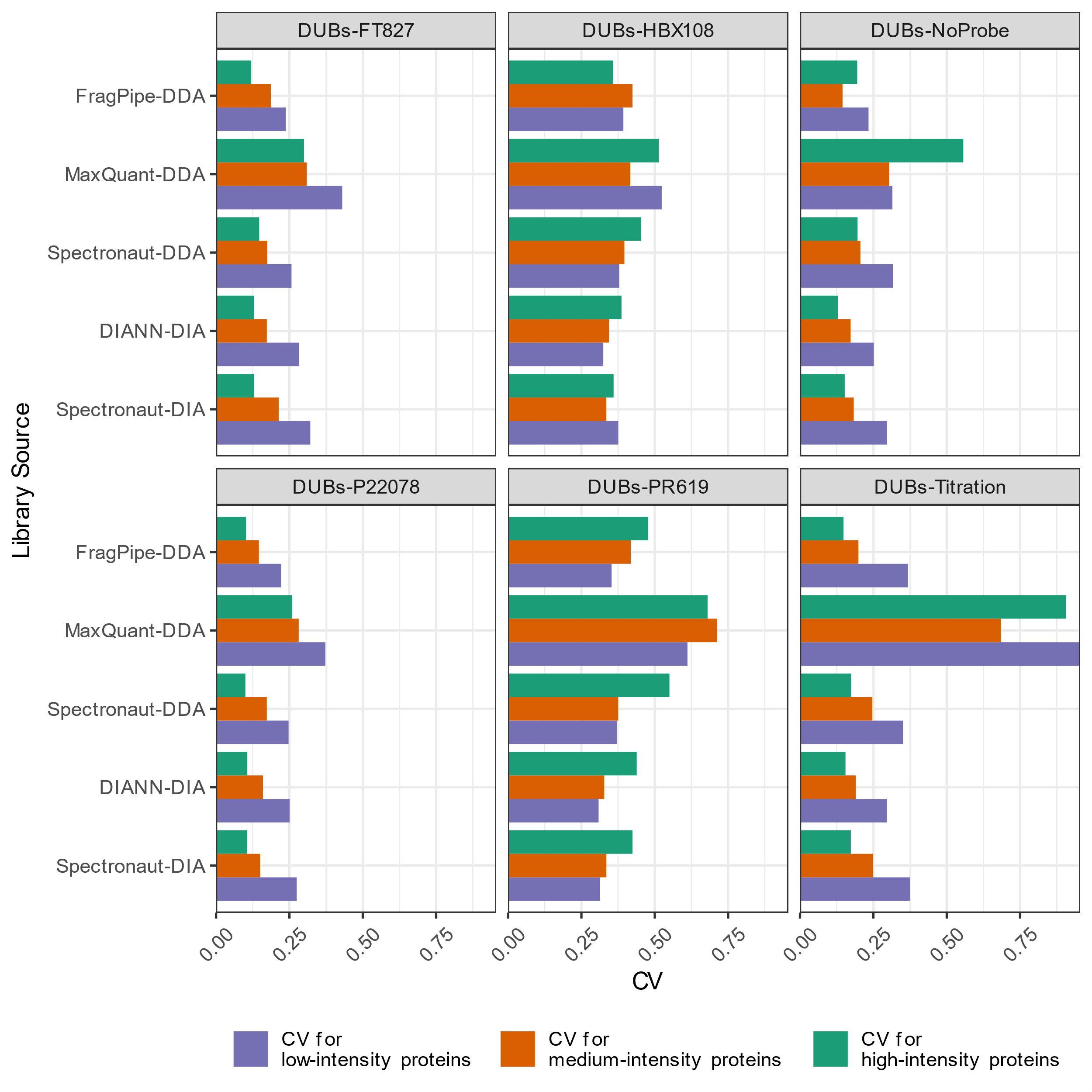


# Text 1: R script for analyzing spectral libraries and protein quantity tables

The script used for producing much of this supplementary material is “20240328-R-Spectral-Libraries.txt”, found in Supplementary File 1 (see below). It contains the following Read functions for importing the spectral libraries created by the various workflows:

- fpRead: Import “library.tsv” for FragPipe and deduplicate it so each row is a peptide rather than a transition.
- sRead: Import “AllPeptides.tsv,” a text export from the Pulsar/Spectronaut KIT format.
- mqRead: Import “evidence.tsv,” the list of peptides identified by MaxQuant (but transitions are enumerated in “msms.tsv”).
- nnRead: Import the “report-lib.tsv” spectral library reported by DIA-NN.

Each of these functions will produce a data.frame that contains these fields: Genes (when the gene symbol to which this peptide is known), Proteins (the set of proteins that could produce this peptide), Peptides (the bare sequence of the peptide), Modified Peptides (the sequence plus the PTMs it bears), and MPZs (which adds precusor charge to the Modified Peptides field).

The protein quantity tables produced by each workflow also feature reader functions:

- sQuant and sQuantComma: Import “Report.tsv” from Spectronaut; if Microsoft Windows localization is set to Europe rather than the US, you may have a comma as a floating point separator rather than a period.
- mqQuant: Import “proteinGroups.txt” from MaxDIA.
- nnQuant: Import “pg_matrix.tsv” from DIA-NN. Because FragPipe incorporates DIA-NN for quantitation, we simply make an alias to the nnQuant reader function called “fpQuant.”
- fpMBRIntensity and fpMBRMaxLFQIntensity: to compare DIA quantity tables to DDA quantity tables, we needed to import “combined_protein.tsv” files created by IonQuant in FragPipe. The software reports intensities in both Intensity and MaxLFQ Intensity columns, so we created separate reader functions for each.

Our goal was to evaluate CVs and missingness in these tables, but we make no effort to compare the intensities seen for a given protein in multiple search / quantitation engines. In fact, the quantity table readers *discard protein accession information*. We did this because trying to untangle situations where two search engines are talking about the same protein but use different isoform numbers, or where two search engines have different protein groups containing a given accession, would create a mess for interpretation.

Three custom reporter functions supplied the protein diversity, CV, and missingness analyses. DiversityStats accepts a table created by one of the reader functions above to count the number of distinct gene symbols, protein accessions (which may be protein groups for some algorithms), distinct peptide sequences, etc for each spectral library. CVbyRow is a helper function, designed to compute the standard deviation and mean quantity for a given protein and then return the ratio (the CV). QuantityStats actually calls the CVbyRow function in its call to “apply” once it has broken out the quintile sets of proteins based on intensity sum. Missingness is evaluated by computing the number of proteins with any intensity reported and the number of proteins with all intensities reported (using a count of “is.na” values on protein quantities).

# Text 2: Low peptide diversity in mock controls


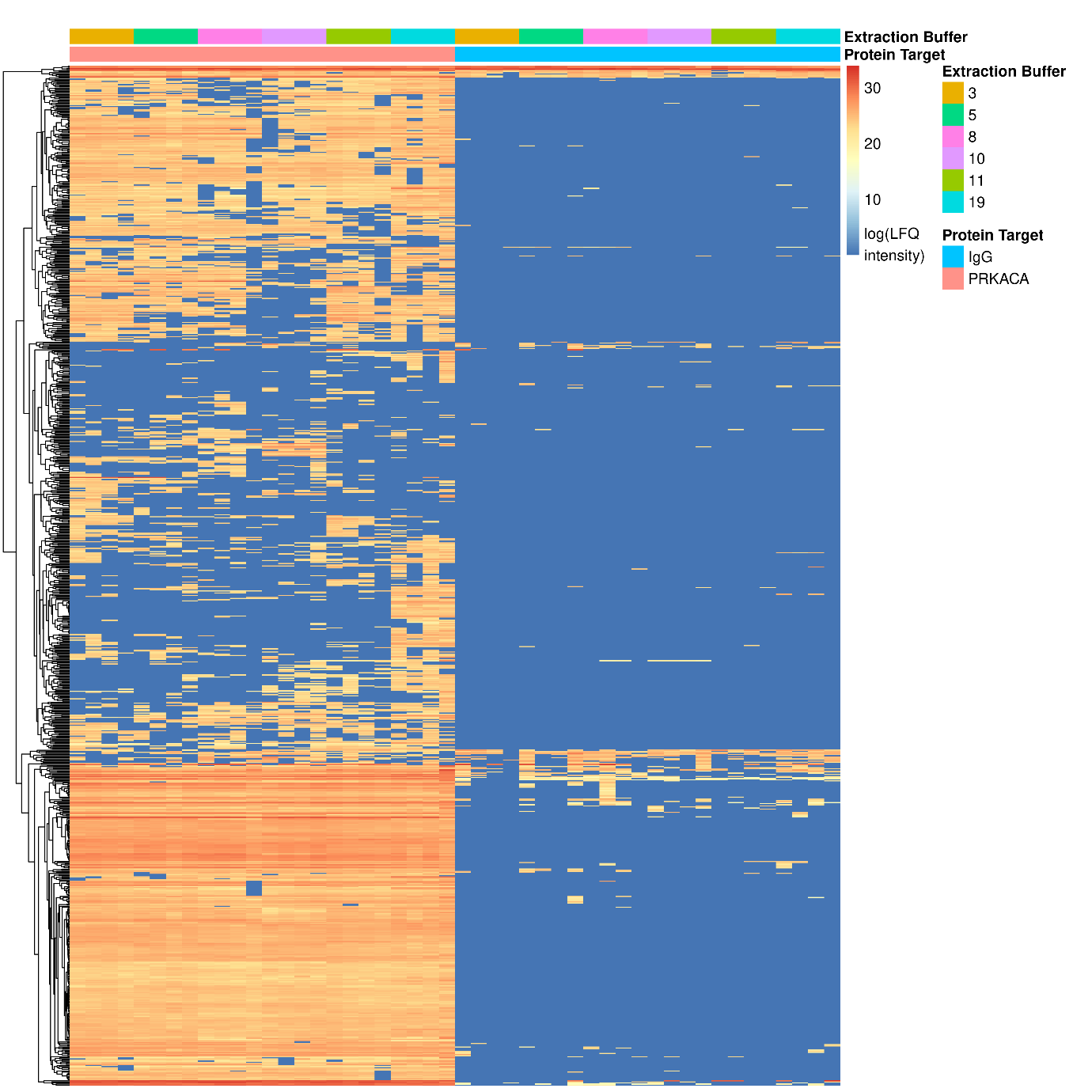


The above image visualizes protein differences between mock co-IPs and genuine co-IPs. Six sets of extraction buffers (four DDA replicates each) were used to explore candidate interactors of a target protein in a series of genuine co-IPs (LEFT). Identical conditions were used in cognate mock co-IPs, carried out using naive polyclonal IgG from the same species as the genuine antibody (RIGHT). Different co-IP conditions were used to explore the effects of the different *in vitro* solution environments on the apparent stabilities of bona fide *in vivo* interactors of the target protein, as well as their ability to mitigate the accumulation of spurious post-lysis interactions (Hakhverdyan et al. 2015; LaCava et al. 2016; Dou et al. 2020).

Notably, very few proteins are present in the mock co-IP controls. When the co-IP workflow is well optimized (relatively inert solid phase medium, high-affinity and high-selectivity antibodies (Johnson and Pan 2014; Bradbury and Plückthun 2015; Baker 2015; Voskuil 2017; Acharya et al. 2017), among other factors, the genuine co-IP and the mock co-IP will have very little in common; they are sampling different populations and the mock co-IP will evaluate (for the most part) only what randomly binds to naive IgGs of the given species-- which is not a good match for the sources of noise appearing in the genuine co-IP.

The noise in the genuine co-IP will emanate from at least two sources: (1) off-target binding to the genuine antibody paratope (i.e., loss of antibody selectivity when the paratope is in excess of the target antigen epitope, which should be its highest affinity binder), which can typically be solved by titrating the affinity medium to the correct quantity for the target protein abundance; and (2) spurious interactions formed *in vitro* with the target itself (directly) and/or with other proteins interacting within the co-IP (indirectly). With that said, neither the genuine paratope in question nor the target and its interactors are present in the mock co-IP, limiting the value of this kind of control. If the mock co-IP and the genuine co-IP detect many proteins in common, the biochemical quality of the co-IP is low and is noisy due to the factors described above (LaCava et al. 2015; LaCava et al. 2016; Ardeljan et al. 2020).

**What is a mock co-IP?**

In the case above, the mock co-IP was a pool of purified polyclonal IgGs from the same species as the genuine antibody (note: these can also be matched for Ig isotype). This kind of control is deficient for the reasons stated above. Additional kinds of mock co-IPs can also be described as follows:

When targeting an affinity tagged protein in an engineered cell line, the genuine antibody can be used in a mock co-IP in the parental cell lines (lacking the tag). This mock will have the same paratopes as the genuine co-IP and can reveal the noise that accumulates from changes in selectivity of the antibody in the absence of its bona fide target (which will greatly depend on the experimental procedures and solution conditions used). If the genuine IP uses a well-titrate quantity of affinity medium that accords with the abundance of the target tagged protein in the tagged cell population, this control may not be very informative.

When targeting an endogenous protein (no affinity tag), the mock co-IP could include the use of the genuine antibody in the cognate cells where the protein has been knocked out (KO, e.g., by CRISPR genome editing) or knocked down (KD, e.g., by RNAi or CRISPRi). The absolute absence of the target protein by KO will result in a situation comparable to the absence of an affinity tag (as above). One must also consider the possible proteomic effects of loss of the target, or the effects of off-target CRISPR edits on the expressed genome. The reduction of the target protein by KD (which is rarely complete) will reduce the absolute power to discern candidate interactors (i.e., reduced effect size) because of residual binding of the target and associated proteins in both genuine and mock co-IPs and will likely increase off-target paratope binding that may not be present in the genuine co-IP, if well titrated for target abundance. This control does, however, allow the determination of proteins whose abundances in the co-IP track closely with the abundance of the target (which KO does not). A related case is comparing, e.g., a tumor tissue (target gene product expressed or over-expressed) and “matched normal” tissue (target gene product not expressed or differentially expressed) where the genuine antibody is used in both tissues (Ardeljan et al. 2020). However, it should be kept in mind that tumor and normal tissue have very different proteomes and will accumulate different types and degrees of post-lysis noise as a result.

In a well optimized workflow, most of the noise will typically emanate from spurious post-lysis binding events that occur with the target protein and associated proteins (although artifacts that also occur *in vivo* through any intervention should not be discounted). Any changes in the proteome in the control cell-type compared to the target cell-type may also change the spurious post-lysis interactors that can decorate the antibody and the target (and will not be easily discernible from those that occurred *in vivo*, e.g., through experimental interventions – whether biologically relevant or artifactual from the intervention). The degree to which these effects manifest is generally tunable through experimental handling and solution conditions which may reduce the absolute burden of false positives but rarely provides unambiguous discrimination of false positives (*in vitro* noise) from true positives (*in vivo* binders that originated in the cells and remained bound for the duration of the co-IP). For this reason, multiple controls, even for all their individual shortcomings, are recommended. And, importantly, orthogonal cross-validation of the biological relevance of putative true positive hits is necessary. To our knowledge the best way to discern *in vivo* binders from post-lysis effects in a co-IP experiment is the I-DIRT method (Tackett et al. 2005; Taylor et al. 2013).

Acharya P, Quinlan A, Neumeister V (2017) The ABCs of finding a good antibody: How to find a good antibody, validate it, and publish meaningful data. F1000Res 6:851. https://doi.org/10.12688/f1000research.11774.1

Ardeljan D, Wang X, Oghbaie M, Taylor MS, Husband D, Deshpande V, Steranka JP, Gorbounov M, Yang WR, Sie B, Larman HB, Jiang H, Molloy KR, Altukhov I, Li Z, McKerrow W, Fenyö D, Burns KH, LaCava J (2020) LINE-1 ORF2p expression is nearly imperceptible in human cancers. Mobile DNA 11:1. https://doi.org/10.1186/s13100-019-0191-2

Baker M (2015) Antibody anarchy: A call to order. Nature 527:545–551. https://doi.org/10.1038/527545a

Bradbury A, Plückthun A (2015) Reproducibility: Standardize antibodies used in research. Nature 518:27–29. https://doi.org/10.1038/518027a

Dou Y, Kalmykova S, Pashkova M, Oghbaie M, Jiang H, Molloy KR, Chait BT, Rout MP, Fenyö D, Jensen TH, Altukhov I, LaCava J (2020) Affinity proteomic dissection of the human nuclear cap-binding complex interactome. Nucleic Acids Research 48:10456–10469. https://doi.org/10.1093/nar/gkaa743

Hakhverdyan Z, Domanski M, Hough LE, Oroskar AA, Oroskar AR, Keegan S, Dilworth DJ, Molloy KR, Sherman V, Aitchison JD, Fenyö D, Chait BT, Jensen TH, Rout MP, LaCava J (2015) Rapid, optimized interactomic screening. Nat Methods 12:553–560. https://doi.org/10.1038/nmeth.3395

Johnson M, Pan H (2014) Antibody Quality. MATER METHODS 4. https://doi.org/10.13070/mm.en.4.572

LaCava J, Jiang H, Rout MP (2016) Protein Complex Affinity Capture from Cryomilled Mammalian Cells. JoVE 54518. https://doi.org/10.3791/54518

LaCava J, Molloy KR, Taylor MS, Domanski M, Chait BT, Rout MP (2015) Affinity Proteomics to Study Endogenous Protein Complexes: Pointers, Pitfalls, Preferences and Perspectives. BioTechniques 58:103–119. https://doi.org/10.2144/000114262

Tackett AJ, DeGrasse JA, Sekedat MD, Oeffinger M, Rout MP, Chait BT (2005) I-DIRT, A General Method for Distinguishing between Specific and Nonspecific Protein Interactions. J Proteome Res 4:1752–1756. https://doi.org/10.1021/pr050225e

Taylor MS, LaCava J, Mita P, Molloy KR, Huang CRL, Li D, Adney EM, Jiang H, Burns KH, Chait BT, Rout MP, Boeke JD, Dai L (2013) Affinity Proteomics Reveals Human Host Factors Implicated in Discrete Stages of LINE-1 Retrotransposition. Cell 155:1034–1048. https://doi.org/10.1016/j.cell.2013.10.021

Voskuil JLA (2017) The challenges with the validation of research antibodies. F1000Res 6:161. https://doi.org/10.12688/f1000research.10851.1

# File 1: Zip of compressed spectral libraries and protein quantity tables

All of the spectral libraries and protein quantity tables for this project have been compressed into a single archive, sorted by the data set in which they were generated. A few files in the main directory could benefit from further description:

- 20240328-R-Spectral-Libraries.txt represents the R code that produced almost all tables and figures from the spectral libraries and protein quantity tables. The bar graph visualizations were handled in a separate codebase that is not included here.
- 20240626-DIANN-MMTS-config.txt and 20240626-FragPipe-MMTS-config.txt illustrate the configuration options that must be included for successful identification of data where MMTS rather than iodoacetamide modified Cys side chains.
- Basic Quality Metrics.xlsx reports some essential details about each LC-MS/MS experiment, including the startTimeStamp that represents the actual time at which data acquisition began for each experiment.
- Tables 1 and 2 from the manuscript have been included in Microsoft Excel form.
