# Supplementary material for: Interrogating data-independent acquisition LC–MS/MS for affinity proteomics
Source: J Proteins Proteom. 2024 Sep 17;15(3):281–98. doi: 10.1007/s42485-024-00166-4 (PMC11452513; doi:10.1007/s42485-024-00166-4)
Supplement: Supplementary file 2 — Supplementary file2 (ZIP 700861 KB) [file 42485_2024_166_MOESM2_ESM.zip › Supplementary-Information/~WRL0003.tmp]

# Supplementary Information for

Interrogating Data-Independent Acquisition LC-MS/MS for affinity proteomics

# Table 1: Cohort definitions for each data set

Each of the six data sets can be subdivided to cohorts that were more uniformly collected and which should have more similar peptide content. These tables report the sets of raw data that correspond to each cohort label within a data set.

## Table 1A: Thermo IP Inputs

## Table 1B: Thermo SPION

## Table 1C: Thermo LINE-1

## Table 1D: SCIEX RACK1

## Table 1E: SCIEX ID4

## Table 1F: Bruker DUBs

# Table 2: Quality metrics for each LC-MS/MS experiment

## Table 2A: Thermo IP Inputs

## Table 2B: Thermo SPION

## Table 2C: Thermo LINE-1

## Table 2D: SCIEX RACK1

## Table 2E: SCIEX ID4

## Table 2F: Bruker DUBs

# Table 3: Spectral library diversity metrics

For each cohort in each data set, we reported the diversity of each spectral library in terms of the number of different gene names reported, the number of distinct protein groups reported, the number of distinct peptides reported, the number of distinct peptides in a given modification state reported, and the number of charge state variants for a given modification state of a given peptide (MPZs) reported.

## Table 3A: Thermo IP Inputs

## Table 3B: Thermo SPION

## Table 3C: Thermo LINE-1

## Table 3D: SCIEX RACK1

## Table 3E: SCIEX ID4

## Table 3F: Bruker DUBs

# Table 4: Protein quantity table protein diversity and CV values

To evaluate protein quantity tables, we first reported the number of proteins in which any quantity was reported and the number of proteins for which all quantities were reported (“unanimous proteins”). We separated unanimous proteins to quintiles based on the sum of intensity values. Then we computed the CV (standard deviation divided by mean intensity) for each protein and reported the median CV within each quintile.

## Table 4A: Thermo IP Inputs

## Table 4B: Thermo SPION

## Table 4C: Thermo LINE-1

## Table 4D: SCIEX RACK1

## Table 4E: SCIEX ID4

## Table 4F: Bruker DUBs

# Figure 1: UpSet plots comparing spectral library peptide content

# Figure 2: Protein diversity for spectral libraries, any quantitation, and unanimous quantitation

# Text 1: R script for analyzing spectral libraries and protein quantity tables

# File 1: Zip of compressed spectral libraries and protein quantity tables
